# Design, Synthesis and Biological Evaluation of Novel Anthraniloyl-AMP Mimics as PQS Biosynthesis Inhibitors Against *Pseudomonas aeruginosa* Resistance

**DOI:** 10.3390/molecules25133103

**Published:** 2020-07-07

**Authors:** Shekh Sabir, Sujatha Subramoni, Theerthankar Das, David StC. Black, Scott A. Rice, Naresh Kumar

**Affiliations:** 1 School of Chemistry, Faculty of Science, The University of New South Wales, Sydney, NSW 2052, Australia; s.sabir@student.unsw.edu.au (S.S.); d.black@unsw.edu.au (D.S.B.); 2 Singapore Centre for Environmental Life Sciences Engineering (SCELSE), Nanyang Technological University, Singapore 637551, Singapore; subramoni@ntu.edu.sg (S.S.); rscott@ntu.edu.sg (S.A.R.); 3 Department of Infectious Diseases and Immunology, School of Medical Sciences, The University of Sydney, Sydney, NSW 2006, Australia; das.ashishkumar@sydney.edu.au

**Keywords:** *Pseudomonas* quinolone system, quorum sensing, *Pseudomonas aeruginosa*, virulence factors, biofilm, anthraniloyl-AMP mimics, pyocyanin production

## Abstract

The *Pseudomonas* quinolone system (PQS) is one of the three major interconnected quorum sensing signaling systems in *Pseudomonas aeruginosa*. The virulence factors PQS and HHQ activate the transcription regulator PqsR (MvfR), which controls several activities in bacteria, including biofilm formation and upregulation of PQS biosynthesis. The enzyme anthraniloyl-CoA synthetase (PqsA) catalyzes the first and critical step in the biosynthesis of quinolones; therefore, it is an attractive target for the development of anti-virulence therapeutics against *Pseudomonas* resistance. Herein, we report the design and synthesis of novel triazole nucleoside-based anthraniloyl- adenosine monophosphate (AMP) mimics. These analogues had a major impact on the morphology of bacterial biofilms and caused significant reduction in bacterial aggregation and population density. However, the compounds showed only limited inhibition of PQS and did not exhibit any effect on pyocyanin production.

## 1. Introduction

Antimicrobial resistance (AMR) is a growing threat to global public health. The Organization for Economic Co-operation and Development (OECD) has predicted that resistant microbial infections would be the leading cause of mortality in the next 30 years, and that around 2.4 million people will die in Europe, North America, and Australia alone. The problem of AMR is even more acute in low and middle-income countries, such as India, Brazil, and Indonesia, where 40–60% of infections are already caused by multidrug-resistant microbes [1,2].

The rapid rise of AMR has created fears that the medical system will soon become overwhelmed with “superbugs”, which are pathogenic bacterial strains that fail to respond to any antibiotic treatment [3]. Recently, the World Health Organization (WHO) has submitted a list of pathogenic bacterial strains that require the most urgent attention for therapeutic intervention [4]. One of the bacteria in the critical priority class is *Pseudomonas aeruginosa*. Multidrug-resistant *P. aeruginosa* strains are a leading cause of acute and chronic infections, particularly in immunocompromised patients such as those suffering from other illnesses including cancer, burns and cystic fibrosis [5]. Furthermore, *P. aeruginosa* can switch from a planktonic (free-living) form to a sessile (community) form by the formation of a biofilm, which greatly contributes to drug resistance. Bacteria in biofilms are about 1000-fold more resistant to antibiotics than planktonic bacteria [6,7].

Only a few classes of antibacterial agents like Oxazolidinones and Lipopeptides with new modes of action have been approved for clinical use since the 1970s. Most of the antibacterial drugs introduced over the last several decades have been obtained by structural modification of existing drugs like Amoxicillin and Azithromycin. As these analogues possess similar mechanisms of action as their predecessors, they are highly susceptible to AMR [8,9]. In this context, there is an urgent need for the development of drugs that inhibit the colonization and virulence of bacteria without exerting selective pressure on bacteria to develop resistance.

Quorum sensing (QS) is an intercellular communication system in bacteria. It is a chemical process which involves the synthesis and release of diffusible quorum sensing signaling molecules (QSSMs), also known as autoinducers (AIs). When the bacterial population grows, the concentration of these AIs in the surrounding environment increases. After a certain threshold is passed, the AIs will activate multiple genes that regulate the collective behavior of bacteria, including activities related to virulence, motility, swarming, biofilm formation and antibiotic resistance [10,11,12].

Inhibition of QS-mediated bacterial communication by small molecules is now considered to be contemporary approach to overcome AMR. The main idea is to inhibit bacterial virulence factors and attenuate biofilm formation without affecting bacterial growth. QS inhibitors (QSIs) do not kill bacteria, and therefore, exert minimum selective pressure on bacteria to develop AMR. Furthermore, QSIs can be used as a combination therapy with conventional antibiotics to give synergistic effects, particularly when the infection is caused by resistant bacteria [13,14,15].

*P. aeruginosa* employs *las*, *rhl* and *pqs* as its major QS systems. Although these QS systems use different kinds of AIs, they are interconnected and modulate the activities of each other. For example, las upregulates the *rhl* and *pqs* systems, while *rhl* downregulates pqs [16,17]. The las and rhl systems use the structurally related chemical class of *N*-acyl homoserine lactones (AHLs) as AIs. These molecules bind and activate their corresponding LuxR-type cognate receptor proteins LasR and RhlR, which leads to the upregulation of the expression of genes associated with virulence and biofilm formation [18].

In contrast, the *Pseudomonas* quinolone signaling (*pqs*) system is based on the chemical class of 2-alkyl-4(1*H*)-quinolones (AQs) as signal molecules. In *P. aeruginosa*, 2-heptyl-3-hydroxy-4(1*H*)-quinolone (PQS) and its immediate biosynthetic precursor, 2-heptyl-4-hydroxyquinoline (HHQ), are the most common QSSMs. However, there are more than 50 other AQs, such as 2-alkyl 4-hydroxy quinoline *N*-oxide and other C-9 congeners of PQS and HHQ. All these molecules activate the protein receptor PqsR (also known as MvfR), leading to the upregulation of the pqsABCDE operon, which further promotes the biosynthesis of PQS through positive feedback as well as increasing the expression of genes associated with bacterial defense mechanisms, including biofilm formation and production of virulence factors [17]. Furthermore, PQS-mediated bacterial aggregation and biofilm formation in *P. aeruginosa* are triggered through different mechanisms, including chemical signaling among bacterial cell population, production of iron-chelating molecules (siderophore), virulence factor pyocyanin biosynthesis and regulation of extracellular DNA (eDNA) release through prophage induction, vesicles production and pyocyanin mediated eDNA release [19,20,21,22].

**Figure 1:**
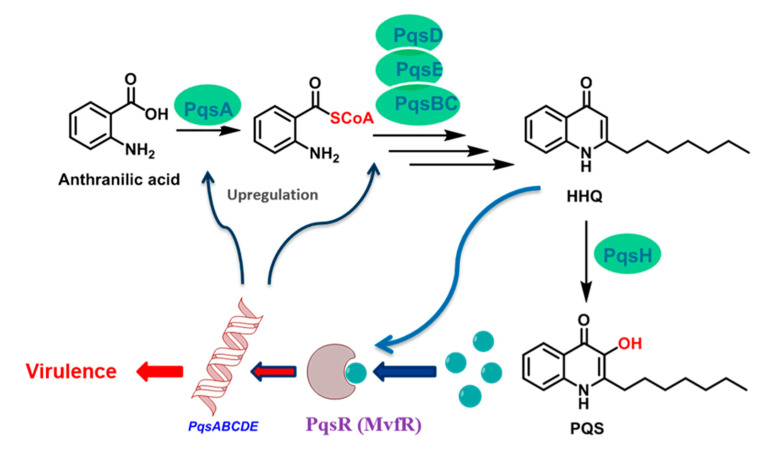
Schematic diagram of PQS biosynthesis, leading to activation of PqsR (MvfR) and expression of the pqsABCDE operon.

Biosynthesis of AQs involves several different enzymatic reactions. The initial substrate is anthranilic acid, which is converted to anthranilate–CoA by the enzyme PqsA (Figure 1). Anthranilate–CoA is transformed through multiple steps into HHQ by enzymes PqsD, PqsE and PqsBC. Finally, the enzyme PqsH catalyzes the hydroxylation of HHQ to form PQS. Hence, the two main ways to block the PQS QS system are to inhibit PQS biosynthesis by targeting its biosynthetic enzymes, and prevention of signal transduction by PqsR receptor antagonists [23,24,25].

**Figure 2:**
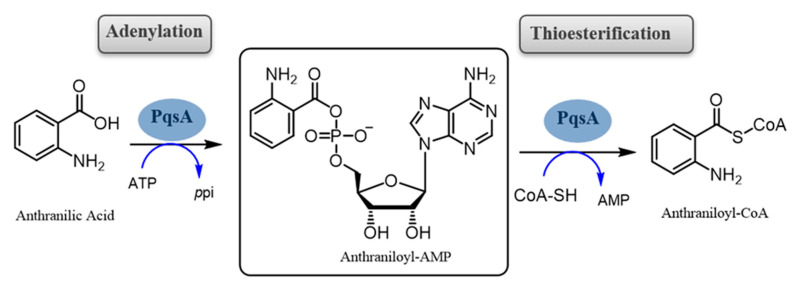
PqsA catalyzes the conversion of anthranilic acid to anthraniloyl-CoA—the first step of PQS biosynthesis.

PqsA is an acyl-CoA synthetase enzyme belonging to the ANL superfamily of structurally related adenylating enzymes. PqsA catalyzes the first and key step in the biosynthesis of PQS, which involves transformation of anthranilic acid to anthranilate–CoA. Mechanistically, PqsA first catalyzes the ATP-dependent adenylation of anthranilic acid to form an anthranilate-AMP intermediate, which subsequently reacts with CoA-SH in a thioesterification reaction to yield anthranilate–CoA (Figure 2). PqsA possesses some attractive features that validates it as a promising target for antimicrobial drug discovery. For example, PqsA-deficient mutant strains show reduced HHQ and PQS biosynthesis and poor biofilm formation in cell culture, as well as highly attenuated virulence in animal infection models. PqsA-deficient mutants also exhibit increased sensitivity towards antimicrobial therapy in vivo. PqsA inhibitors decrease PQS production and reduce mortality in a mice infection model [26]. Importantly, PqsA is exclusively present in bacterial cells, therefore, selective PqsA inhibitors are unlikely to affect host cell viability.

**Figure 3:**
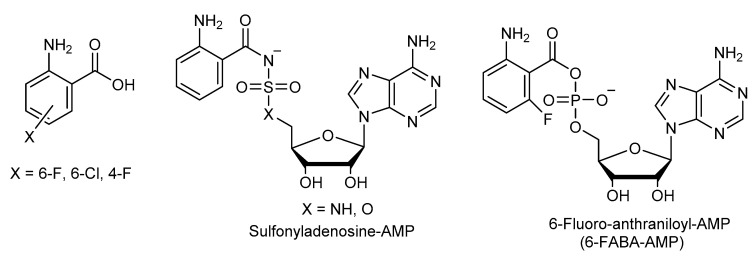
Previously reported inhibitors of PqsA.

Despite its essential role in the biosynthesis of PQS, only few attempts to inhibit the PqsA enzyme have been reported so far (Figure 3). Halogenated anthranilic acids have been reported as competitive inhibitors of PqsA, however, they showed only weak inhibition, as millimolar concentrations are required to show any effect [23]. Recently, Ji et al. reported sulfonyl adenosine-based small molecule inhibitors mimicking the anthraniloyl-AMP intermediate for the competitive inhibition of PqsA. While these inhibitors showed high binding affinity (K_i_ < 0.2 μM) for the PqsA enzyme, they did not exhibit satisfactory antibacterial potency in vitro (IC_50_ > 300 μM). Compound accumulation studies showed poor bacterial cell permeability, which possibly limited their cellular activity [26]. Recently, the crystal structures of PqsA complexed with the ligand anthraniloyl-AMP with the inhibitor 6-fluoroanthraniloyl-AMP (6-FABA-AMP) were reported by Witzgall et al. [27]. The structures revealed that the N-terminal domain of PqsA contains the active site that recognizes and binds the natural ligand anthraniloyl-AMP as well as the competitor inhibitor, 6-FABA-AMP.

## 2. Design and Synthesis of Novel PqsA Inhibitors

Previously reported PqsA inhibitors based on anthranilate-AMP have shown high binding affinity and promising activity in enzymatic assays. However, due to their highly polar nature, these inhibitors cannot penetrate the bacterial cell membrane, and are therefore, devoid of any cellular activity in *P. aeruginosa*, which limits their further development as novel antimicrobial agents.

**Figure 4:**
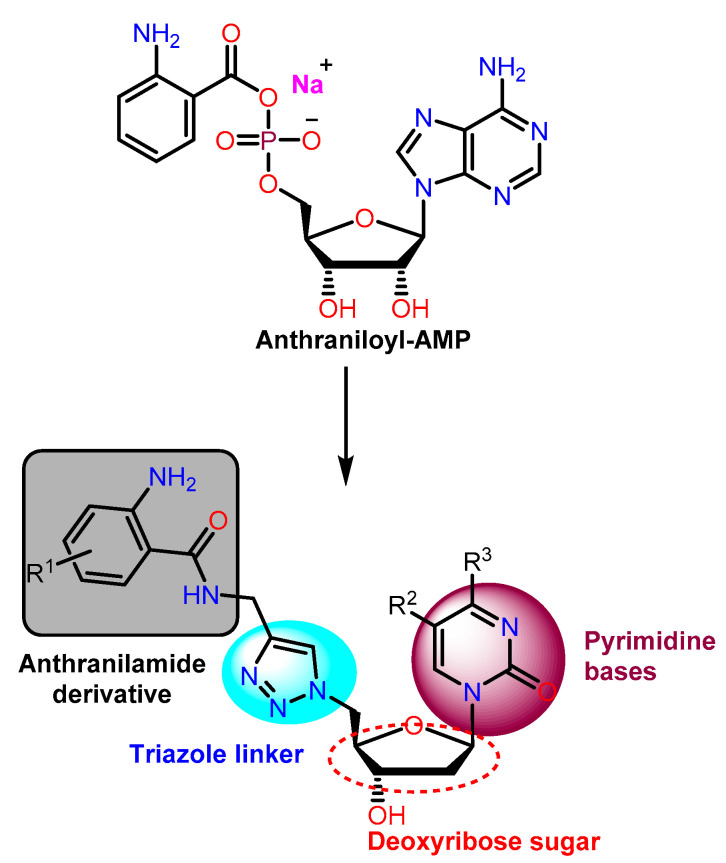
Ligand-based design of novel PqsA inhibitors.

Nucleotide analogues can be generated by modifications of the phosphate backbone, ribose sugar or base [28]. To develop nonpolar nucleotide analogues of anthraniloyl-AMP, we first used the neutral triazole linker as an isostere for the charged phosphodiester linkage. One advantage of using triazole is that it is metabolically more stable than the phosphodiester linkage, because of its resistance to degradation by nuclease. The second modification was to replace the adenine base of anthraniloyl-AMP with pyrimidine bases, such as thymidine, 2′-deoxyuridine and 2′-deoxycytidine [29]. Finally, the ribose sugar of the natural ligand was replaced with 2′-deoxyribose (Figure 4).

**Scheme 1:**
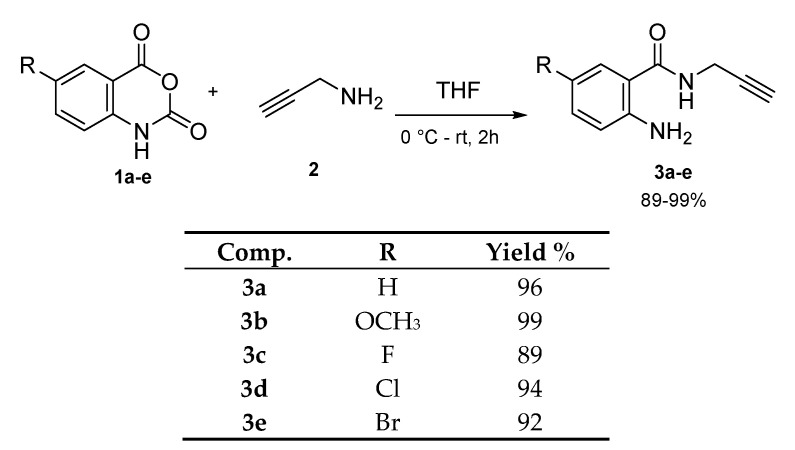
Synthesis of anthranilamide intermediates.

The synthetic route for the synthesis of the novel triazole-linked anthranilamide deoxynucleoside compounds began with the ring-opening of 5-substituted isatoic anhydrides (**1a–e**) using propargylamine (**2**) under mild reaction conditions. Different terminal alkyne-containing anthranilamide intermediate compounds (**3a–e**) were synthesized in excellent yields (Scheme 1).

**Scheme 2:**
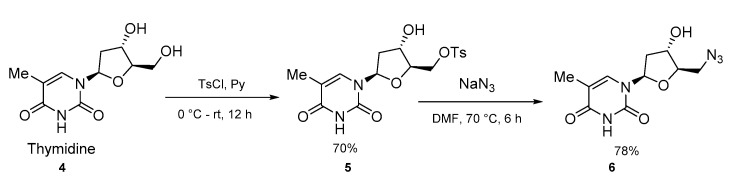
Synthesis of thymidine azide intermediate **6**.

Next, we synthesized nucleoside azides following a reported protocol [30]. Selective tosylation of the primary hydroxy group of thymidine nucleoside (**4**) using pyridine and tosyl chloride at 0 °C generated tosylate (**5**), which was further converted to the corresponding azide (**6**) by a nucleophilic substitution reaction with sodium azide in DMF at 70 °C (Scheme 2).

**Scheme 3:**
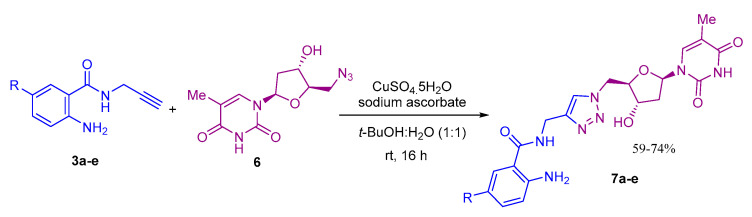
Synthesis of thymidine nucleotide analogues **7a–e**.

The final step involved an azide–alkyne cycloaddition (click chemistry) reaction between alkynes (**3a–e**) and thymidine azide (**6**). The reaction was performed in the presence of CuSO_4_ catalyst and sodium ascorbate as reducing agents in *t*-BuOH:H_2_O (1:1) medium [31]. The desired triazolo nucleosides (**7a–e**) were synthesized between 59–74% yields (Scheme 3).

**Scheme 4:**
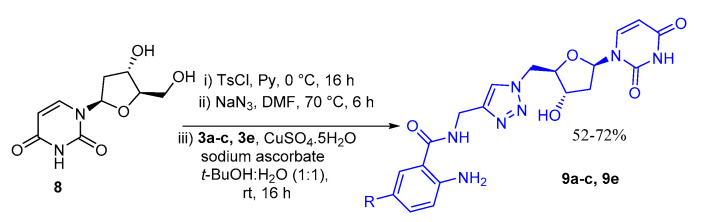
Synthesis of 2′-deoxyuridine nucleotide analogues **9a–c** and **9e**.

Furthermore, 2′-deoxyuridine (**8**) was converted to the corresponding triazole nucleoside analogues (**9a–c**) and (**9e**) by using the same three-step reaction sequence in moderate to good isolated yields (Scheme 4). 

**Scheme 5:**
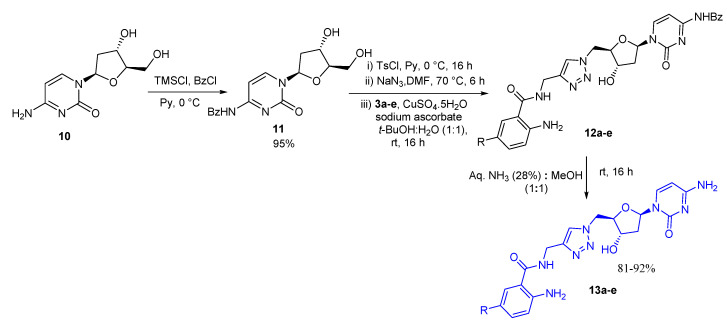
Synthesis of 2′-deoxycytidine nucleotide analogues **13a–e**.

In the case of deoxycytidine (**10**), selective tosylation of the primary alcohol did not give the desired product, most likely due to presence of the free amino group. We attempted to protect the amino group via acetyl and trifluoroacetyl protecting groups, however, both were found to be unstable and did not survive the subsequent tosylation reaction. After conducting a literature search, we found that a benzoyl group could be used for the protection of the amino group of deoxycytidine [32]. Therefore, selective benzoyl protection of deoxycytidine (**10**) was achieved by using TMSCl and benzoyl chloride in pyridine at 0 °C, giving *N*-benzoylated deoxycytidine (**11**) in excellent yields (Scheme 5). Next, compound **(11)** was converted to the benzoyl-protected deoxycytidine triazole analogues (**12a–e**) following the same reaction pathway as above. Finally, the benzoyl group was deprotected by mild hydrolysis using aqueous NH_3_(28%):MeOH (1:1) at room temperature to give the desired deoxycytidine analogues (**13a–e**) in excellent yields, from 81–92%.

**Figure 5:**
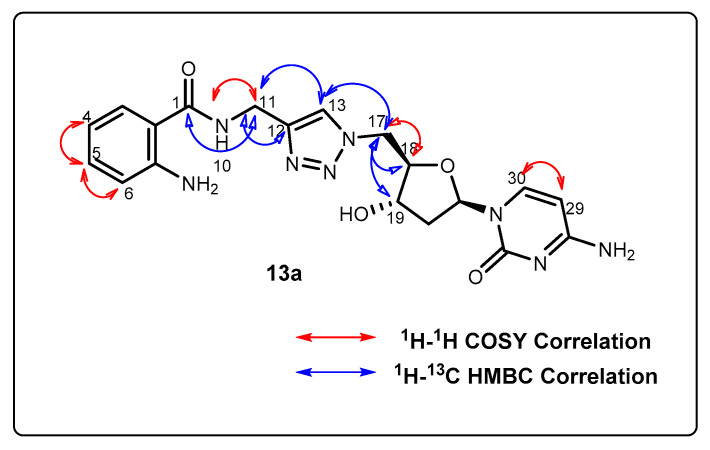
Key COSY and HMBC correlations in compound **13a.**

2D NMR experiments including COSY, HSQC and HMBC were performed to confirm the regioselectivity of the compounds, particularly the connectivity between the anthranilamide unit and the pyrimidine nucleoside via the 1,4-disubstituted triazole ring. In the COSY spectrum of compound **13**a, the CH_2_ protons at C-17 (5′) of the sugar showed correlation with the proton at C-18 (4′) of the sugar (Figure 5). Meanwhile, in the HMBC spectrum of 13a, the C-17 protons showed correlation with C-18 and C-19 (3′) carbons of the sugar, and with C-13 of the triazole ring. Similarly, the CH_2_ protons at C-11 showed a COSY correlation with the N-H proton of the anthranilamide and HMBC correlations with C-1, C-12, and C-13 carbons (Figure 5).

## 3. Results and Discussion

### Biological Activity 

**Figure 6:**
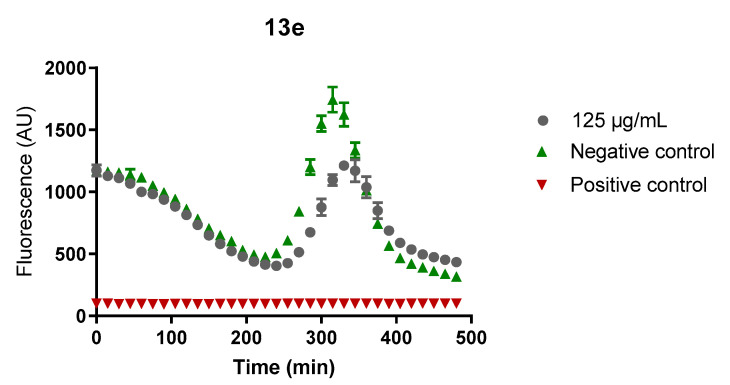
PQS inhibition by compound **13e** using PAO1 *pqsA:gfp* reporter-based assay.

For PQS inhibition assay, all the new triazole nucleotide analogues were screened for their ability to inhibit the pqs QS system using the PAO1 (a *P. aeruginosa* reporter strain) *pqsA:gfp* reporter assay. This assay measures the PqsR (MvfR) regulated expression of the pqsABCDE operon. It is expected that the PqsA inhibitors will block the biosynthesis of PQS and HHQ, which leads to lower activation of the PqsR (MvfR) receptor and ultimately, reduction in the expression of the pqsABCDE reporter. The expression of the reporter in the untreated control reached its maximum level after 6–8 h which then fell to basal expression levels at later time points. A known inhibitor of PQS was employed as a positive control which prevented this expression [33]. Preliminary assays revealed that concentrations of DMSO above 1.25% inhibited GFP expression of the reporter, even without the presence of any of the test compounds, although growth was unaffected. Therefore, the final DMSO concentration was kept at 1.25%. Based on the concentration of our compound stock solutions, this corresponded to 125 µg/mL as the highest concentration tested for each compound. Then, two-fold lower serial dilutions of the compound were tested whilst keeping the DMSO concentration constant. All compounds except one showed no or very slight inhibition of the pqsA reporter at all concentrations tested. Only **13e** at 125 µg/mL (400 µM approx.) showed approximately 30% inhibition of the reporter when compared to the negative control (Figure 6, Table 1). The LogP value of the compound plays a critical role in bacterial cell penetration and the cLogP value of compound **13e** (−0.55) is highest among cytidine triazoles, which indicates that the compound **13e** could be more permeable to bacterial cells compared to all other derivatives.

**Figure 7:**
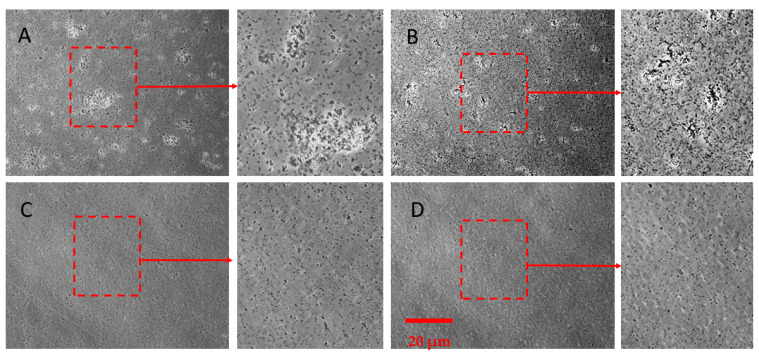
Phase-contrast microscopy images showing morphology of *P. aeruginosa* PAO1 grown in the presence of (**A**) control/untreated; (**B**) Dimethyl sulfoxide (DMSO) only; (**C**) 400 µM 13e; (**D**) 400 µM13d. Scale bar = 20 µm

For inhibition of biofilm formation, some of the representative compounds (**7a**, **7e**, **9a**, **9e**, **13d** and **13e**) were tested for their potential ability to inhibit *P. aeruginosa* biofilm formation. PAO1 planktonic cultures were incubated with 400 μM of compounds and added to microtiter wells to initiate bacterial adhesion and biofilm growth. After 24 h, biofilms were visualized using phase contrast microscopy [34]. The compounds exerted a major impact on the morphology of bacterial biofilms. A drastic reduction in bacterial aggregation and population density was observed in the presence of compounds **13e** and **13d** as compared to both untreated and DMSO controls (Figure 7). Since the PQS system mediates bacterial aggregation and biofilm formation in *P. aeruginosa*, this result could indicate that the compounds interfere with PQS biosynthesis to reduce bacterial aggregation. However, given the weak PQS inhibitory activity of the compounds in the in vitro reporter assay, we do not exclude the possibility that the compounds might be acting through alternative pathways to inhibit biofilm formation.

For the pyocyanin inhibition assay, to investigate the activity of these compounds against bacterial virulence factors, some of the derivatives (**7a**, **7e**, **9a**, **9e**, **13d** and **13e**) were evaluated for their ability to inhibit the production of pyocyanin from *P. aeruginosa* PA01 at 400 µM concentration. However, out of six compounds tested, none of them showed any significant decrease in the production of pyocyanin. The findings in this study match with previously published data showing compounds were effective in inhibiting PQS biosynthesis without any impact on pyocyanin production [26]. This is an indication of the complex relationship between the PQS biosynthesis and pyocyanin production.

Regarding effect on growth, none of the compounds had any effect on growth at concentrations up to 256 µg/mL except for **7a**. For **7a**, even the 32 µg/mL concentration was enough to affect growth and increase the lag phase. For all compounds, growth was inhibited at 512 µg/mL, although this could be due to the amount of DMSO present, since 5.12% DMSO alone affected growth (for details, see Supporting Information).

## 4. Materials and Methods 

### 4.1. Synthesis

All the reagents and solvents were purchased from commercial sources (Combi-Blocks, Oakwood Chemicals, Sigma-Aldrich, Sydney, Australia) and used without further purification. Reactions were performed using oven-dried glassware under an atmosphere of nitrogen (if required). Room temperature refers to the ambient temperature (25 °C). Yields refer to compounds isolated after flash column chromatography unless otherwise stated. Progress of the reactions were monitored by thin layer chromatography (TLC) precoated with Merck silica gel 60 F254 plates and visualization using UV light (254 nm). Flash column chromatography was carried out using Grace Davison LC60A 40–63 µm silica gel as the stationary phase and solvent gradient (methanol in ethyl acetate) used as the mobile phase. High-resolution mass spectrometry (HRMS) was performed at Bioanalytical Mass Spectrometry Facility, UNSW by electrospray (ESI) ionization using a Thermo LTQ Orbitrap XL instrument (Thermo Scientific, Waltham, MA, USA). ^1^H- and ^13^C-NMR was recorded in deuterated solvent (DMSO-*d6*) using Bruker Avance 300, 400 or 600 MHz instruments (Bruker Pty Ltd., Preston, NSW, Australia) at 24 °C. Chemical shifts (δ) are reported as relative to the corresponding solvent peak, with tetramethylsilane as the internal standard and quoted in parts per million (ppm). Multiplicities in the NMR spectra are described as: s—singlet; d—doublet; t—triplet; q—quartet; m—multiplet; b—broad; coupling constants (*J*) are reported in hertz (Hz). 

General procedure (**A**) for the ring opening of isatoic anhydrides (Synthesis of compounds **3a–3e**): To a solution of isatoic anhydride (3.0 mmol) in THF (20 mL), propargyl amine (4.5 mmol) was added at 0 °C, and reaction stirred at rt for 2 h. After completion of the reaction as indicated by TLC, the solvent was evaporated and residue was dissolved in minimum DCM, then, excess hexane was added, and precipitate was collected and dried, which gives desired ring opened terminal alkyne products from 89–99% yields.

2-Amino-5-methoxy-*N*-(prop-2-yn-1-yl)benzamide (**3b**): Following the general procedure A, the title product was obtained as brown solid (607mg, 99%); ^1^H-NMR (400 MHz, DMSO-*d*_6_): δ 8.65 (t, *J* = 5.6 Hz, 1H, NH), 7.06 (d, *J* = 2.9 Hz, 1H, Ar-H), 6.85 (dd, *J* = 8.9, 2.9 Hz, 1H, Ar-H), 6.66 (d, *J* = 8.9 Hz, 1H, Ar-H), 6.03 (s, 2H, NH_2_), 4.00 (dd, *J* = 5.6, 2.5 Hz, 2H, CH_2_), 3.68 (s, 3H, CH_3_), 3.08 (t, *J* = 2.5 Hz, 1H, CH); ^13^C-NMR (101 MHz, DMSO-*d*_6_): δ 168.32, 149.31, 144.17, 120.20, 117.92, 113.80, 111.61, 81.62, 72.60, 55.58, 28.12; HRMS (ESI) *m/z* calcd. for C_11_H_12_N_2_O_2_ [M + H]^+^ 205.0972, found 205.0971.

2-Amino-5-fluoro-*N*-(prop-2-yn-1-yl)benzamide **(3c):** Following the general procedure **A**, the title product was obtained as off-white solid (515 mg, 89%); ^1^H-NMR (400 MHz, DMSO-*d*_6_): δ 8.70 (t, *J* = 5.4, 1H, NH), 7.34 (dd, *J* = 10.3, 3.0 Hz, 1H, Ar-H), 7.06 (td, *J* = 9.0, 8.1, 3.0 Hz, 1H, Ar-H), 6.72 (dd, *J* = 9.1, 5.0 Hz, 1H, Ar-H), 6.35 (s, 2H, NH_2_), 3.99 (dd, *J* = 5.5, 2.5 Hz, 2H, CH_2_), 3.09 (t, *J* = 2.5, 1H, CH); ^13^C-NMR (101 MHz, DMSO–*d6*): δ 167.60, 167.57, 153.75, 151.46, 146.66, 119.67, 119.44, 117.78, 117.71, 113.58, 113.36, 113.30, 81.37, 72.71, 28.20; HRMS (ESI) m/z calcd. For C_10_H_9_FN_2_O [M + H]+ 193.0772, found 193.0771.

2-Amino-5-chloro-*N*-(prop-2-yn-1-yl)benzamide **(3d):** Following the general procedure **A,** the title product was obtained as white solid (591 mg, 94%); ^1^H-NMR (400 MHz, DMSO-*d*_6_): δ 8.77 (t, *J* = 5.3, 1H, NH), 7.55 (d, *J* = 2.5 Hz, 1H, Ar-H), 7.17 (dd, *J* = 8.8, 2.5 Hz, 1H, Ar-H), 6.73 (d, *J* = 8.8 Hz, 1H, Ar-H), 6.59 (s, 2H, NH_2_), 3.98 (dd, *J* = 5.5, 2.5 Hz, 2H, CH_2_), 3.09 (t, *J* = 2.5 1H, CH); ^13^C-NMR (101 MHz, DMSO-*d*_6_): δ 167.41, 148.78, 131.76, 127.39, 118.17, 117.75, 114.46, 81.32, 72.72, 28.21; HRMS (ESI) *m/z* calcd. for C_9_H_10_ClN_2_O [M + H]^+^ 209.0476, found 209.0476.

General procedure (**B**) for the azide–alkyne cycloaddition reaction: To a mixture of nucleoside azide (0.2 mmol) and corresponding alkyne (10a–e) (0.2 mmol) in (1:1) tert-butanol: water (4 mL), sodium ascorbate (0.04 mmol) and CuSO_4_.5H_2_O (0.02 mmol) were added. The reaction mixture was stirred at rt for 16 h. After completion of the reaction as indicated by TLC, solvent was evaporated in vacuo. The crude was purified by silica gel column chromatography (silica gel was pre-neutralized with 1% triethylamine in ethyl acetate) using 0–20% methanol in ethyl acetate gradient system. The polarity was gradually increased after every 100 mL of eluent. Desired compounds were eluted with 15–20% methanol in ethyl acetate system as off-white to light brown solid between 52–74% yields.

2-Amino-*N*-((1-(((*2R,3S,5R*)-3-hydroxy-5-(5-methyl-2,4-dioxo-3,4-dihydropyrimidin-1(*2H*)-yl)tetrahydrofuran-2-yl)methyl)-1*H*-1,2,3-triazol-4-yl)methyl)benzamide (**7a**): Following the general procedure **B**, the title product was obtained as off-white solid (54 mg, 61%); ^1^H-NMR (400 MHz, DMSO-*d*_6_): δ 11.24 (bs, 1H, NH), 8.72 (t, *J* = 5.7 Hz, 1H, NH), 7.93 (s, 1H, Ar-H), 7.49 (dd, *J* = 8.0, 1.5 Hz, 1H, Ar-H), 7.37 (d, *J* = 1.4 Hz, 2H, NH_2_ ), 7.12 (td, *J* = 8.5, 7.1, 1.5 Hz, 1H, Ar-H), 6.68 (dd, *J* = 8.3, 1.2 Hz, 1H, Ar-H), 6.48 (td, *J* = 8.1, 7.1, 1.2 Hz, 1H, Ar-H), 6.42 (s, 2H, NH_2_), 6.17 (t, *J* = 7.7, 6.3 Hz, 1H, CH), 5.48 (d, *J* = 4.3 Hz, 1H, OH), 4.67–4.51 (m, 2H, CH_2_), 4.44 (d, *J* = 5.7 Hz, 2H, CH_2_), 4.30–4.23 (m, 1H, CH), 4.09–4.05 (m, 1H, CH), 2.20–2.04 (m, 2H, CH_2_), 1.78 (d, *J* = 1.2 Hz, 3H, CH_3_); ^13^C-NMR (101 MHz, DMSO-*d*_6_): δ 168.80, 163.64, 150.41, 149.77, 145.45, 136.01, 131.79, 128.11, 123.59, 116.35, 114.50, 114.15, 109.88, 84.07, 84.02, 70.79, 51.13, 37.86, 34.46, 12.04; HRMS (ESI) *m/z* calcd. for C_20_H_23_N_7_O_5_ [M + H]^+^ 442.1833, found 442.1832.

2-Amino-*N*-((1-(((*2R,3S,5R*)-3-hydroxy-5-(5-methyl-2,4-dioxo-3,4-dihydropyrimidin-1(*2H*)-yl)tetrahydrofuran-2-yl)methyl)-1*H*-1,2,3-triazol-4-yl)methyl)-5-methoxybenzamide (**7b**): Following the general procedure **B**, the title product was obtained as light brown solid (56 mg, 59%) ^1^H-NMR (300 MHz, DMSO-*d*_6_): δ 11.31 (bs, 1H, NH), 8.79 (t, *J* = 5.7 Hz, 1H, NH), 7.95 (s, 1H, Ar-H), 7.38 (d, *J* = 1.5 Hz, 1H, Ar-H), 7.10 (d, *J* = 2.9 Hz, 1H, Ar-H), 6.85 (dd, *J* = 8.8, 2.9 Hz, 1H, Ar-H), 6.67 (d, *J* = 8.9 Hz, 1H, Ar-H), 6.17 (t, *J* = 7.0 Hz, 3H, CH, NH_2_), 5.49 (d, *J* = 4.3 Hz, 1H, OH), 4.65 (dd, *J* = 15.7, 6.0 Hz, 2H, CH_2_), 4.46 (d, *J* = 5.6 Hz, 2H, CH_2_), 4.31–4.22 (m, 1H, CH), 4.13–4.05 (m, 1H, CH), 3.68 (s, 3H, OCH_3_), 2.23–2.04 (m, 2H, CH_2_), 1.79 (s, 3H, CH_3_); ^13^C-NMR (76 MHz, DMSO-*d*_6_): δ 168.47, 163.63, 150.40, 149.43, 145.35, 136.01, 123.64, 119.82, 117.92, 114.58, 111.85, 109.87, 84.06, 84.02, 70.79, 55.57, 51.14, 37.87, 34.47, 12.02; HRMS (ESI) *m/z* calcd. for C_20_H_25_N_7_O_6_ [M + Na]^+^ 494.1759, found 494.1755.

2-Amino-5-fluoro-*N*-((1-(((*2R,3S,5R*)-3-hydroxy-5-(5-methyl-2,4-dioxo-3,4-dihydropyrimidin-1(*2H*)-yl)tetrahydrofuran-2-yl)methyl)-1*H*-1,2,3-triazol-4-yl)methyl)benzamide (**7c**): Following the general procedure **B**, the title product was obtained as off-white solid (60 mg, 65%) ^1^H-NMR (400 MHz, DMSO-*d*_6_): δ 11.29 (bs, 1H, NH), 8.80 (t, *J* = 5.6 Hz, 1H, NH), 7.96 (s, 1H, Ar-H), 7.40–7.30 (m, 2H, Ar-H), 7.04 (td, *J* = 8.5, 2.9 Hz, 1H, Ar-H), 6.70 (dd, *J* = 9.0, 5.0 Hz, 1H, Ar-H), 6.31 (s, 2H, NH_2_), 6.17 (t, *J* = 7.0 Hz, 1H, CH), 5.48 (d, *J* = 4.2 Hz, 1H, OH), 4.72–4.55 (m, 2H, CH_2_), 4.45 (d, *J* = 5.6 Hz, 2H, CH_2_), 4.30–4.22 (m, 1H, CH), 4.11–4.04 (m, 1H, CH), 2.19–1.99 (m, 2H, CH_2_), 1.78 (s, 3H, CH_3_); ^13^C-NMR (101 MHz, DMSO-*d*_6_): δ 166.72, 163.64, 150.41, 146.20, 136.00, 123.85, 119.58, 119.12, 117.56, 113.66, 113.06, 109.88, 84.04, 70.80, 51.16, 37.88, 34.52, 12.02; HRMS (ESI) *m/z* calcd. for C_20_H_22_FN_7_O_5_ [M + Na]^+^ 482.1559, found 482.1556.

2-Amino-5-chloro-*N*-((1-(((*2R,3S,5R*)-3-hydroxy-5-(5-methyl-2,4-dioxo-3,4-dihydropyrimidin-1(*2H*)-yl)tetrahydrofuran-2-yl)methyl)-1*H*-1,2,3-triazol-4-yl)methyl)benzamide (**7d**): Following the general procedure **B**, the title product was obtained as off-white solid (70 mg, 74%) ^1^H-NMR (400 MHz, DMSO-*d*_6_): δ 11.30 (bs, 1H, NH), 8.87 (t, *J* = 5.7 Hz, 1H, NH), 7.97 (s, 1H, Ar-H), 7.57 (d, *J* = 2.4 Hz, 1H, Ar-H), 7.36 (s, 1H, Ar-H), 7.16 (dd, *J* = 8.8, 2.4 Hz, 1H, Ar-H), 6.71 (d, *J* = 8.8 Hz, 1H, Ar-H), 6.57 (s, 2H, NH_2_), 6.17 (t, *J* = 7.0 Hz, 1H, CH), 5.50 (d, *J* = 4.3 Hz, 1H, OH), 4.74–4.53 (m, 2H, CH_2_), 4.45 (d, *J* = 5.5 Hz, 2H, CH_2_), 4.30–4.25 (m, 1H, CH), 4.12–4.04 (m, 1H, CH), 2.22–2.02 (m, 2H, CH_2_), 1.78 (s, 3H, CH_3_); ^13^C-NMR (101 MHz, DMSO-*d*_6_): δ 167.61, 163.65, 150.42, 148.68, 136.01, 131.56, 127.43, 123.66, 118.06, 117.73, 114.96, 109.90, 84.05, 70.81, 51.17, 37.90, 34.52, 31.31, 12.04; HRMS (ESI) *m/z* calcd. for C_20_H_22_ClN_7_O_5_ [M + H]^+^ 476.1444, found 476.1443.

2-Amino-5-bromo-*N*-((1-(((*2R,3S,5R*)-3-hydroxy-5-(5-methyl-2,4-dioxo-3,4-dihydropyrimidin-1(*2H*)-yl)tetrahydrofuran-2-yl)methyl)-1*H*-1,2,3-triazol-4-yl)methyl)benzamide (**7e**): Following the general procedure **B**, the title product was obtained as light orange solid (68 mg, 65%) ^1^H-NMR (400 MHz, DMSO-*d*_6_): δ 11.29 (bs, 1H, NH), 8.87 (t, *J* = 5.5 Hz, 1H, NH), 7.96 (s, 1H, Ar-H), 7.67 (s, 1H, Ar-H), 7.36 (s, 1H, Ar-H), 7.26 (d, *J* = 8.8 Hz, 1H, Ar-H), 6.67 (d, *J* = 8.8 Hz, 1H, Ar-H), 6.58 (s, 2H, NH_2_), 6.17 (t, *J* = 7.0 Hz, 1H, CH), 5.48 (d, *J* = 4.2 Hz, 1H, OH), 4.87–4.52 (m, 2H, CH_2_), 4.44 (d, *J* = 5.5 Hz, 2H, CH_2_), 4.32–4.22 (m, 1H, CH), 4.15–4.04 (m, 1H, CH), 2.32–2.01 (m, 2H, CH_2_), 1.78 (s, 3H, CH_3_); ^13^C-NMR (101 MHz, DMSO-*d*_6_): δ 167.52, 163.65, 150.42, 149.00, 136.01, 134.25, 130.24, 128.36, 126.47, 123.73, 118.47, 115.58, 109.89, 104.90, 84.04, 70.80, 51.16, 37.90, 34.52, 12.05; HRMS (ESI) *m/z* calcd. for C_20_H_22_BrN_7_O_5_ [M + H]^+^ 520.0938, found 520.0939.

2-Amino-*N*-((1-(((*2R,3S,5R*)-5-(2,4-dioxo-3,4-dihydropyrimidin-1(*2H*)-yl)-3-hydroxytetrahydrofuran-2-yl)methyl)-1*H*-1,2,3-triazol-4-yl)methyl)benzamide (**9a**): Following the general procedure **B**, the title product was obtained as off-white solid (60 mg, 70%); ^1^H-NMR (400 MHz, DMSO-*d*_6_): δ 11.32 (bs, *J* = 2.2 Hz, 1H, NH), 8.72 (t, *J* = 5.7 Hz, 1H, NH), 7.92 (s, 1H, Ar-H), 7.56 (d, *J* = 8.1 Hz, 1H, Ar-H), 7.49 (dd, *J* = 8.0, 1.5 Hz, 1H, Ar-H), 7.13 (td, *J* = 8.4, 7.0, 1.5 Hz, 1H, Ar-H), 6.69 (dd, *J* = 8.3, 1.2 Hz, 1H, Ar-H), 6.56–6.44 (m, 1H, Ar-H), 6.41 (s, 2H, NH_2_), 6.15 (t, *J* = 6.9 Hz, 1H, CH), 5.62 (dd, *J* = 8.1, 2.2 Hz, 1H, Ar-H), 5.51 (s, 1H, OH), 4.67 (dd, *J* = 14.2, 4.5 Hz, 1H, CH_2_), 4.57 (dd, *J* = 14.3, 7.7 Hz, 1H, CH_2_), 4.45 (d, *J* = 5.7 Hz, 2H, CH_2_), 4.25–4.22 (m, 1H, CH), 4.10–4.06 (m, 1H, CH), 2.22–2.04 (m, 2H, CH_2_);^13^C-NMR (101 MHz, DMSO-*d*_6_): δ 168.85, 163.02, 150.41, 149.74, 145.46, 140.72, 131.82, 128.15, 123.59, 116.40, 114.59, 114.26, 102.11, 84.42, 84.28, 70.78, 51.20, 38.08, 34.49; HRMS (ESI) *m/z* calcd. for C_19_H_21_N_7_O_5_ [M + H]^+^ 428.1677, found 428.1673.

2-Amino-*N*-((1-(((*2R,3S,5R*)-5-(2,4-dioxo-3,4-dihydropyrimidin-1(*2H*)-yl)-3-hydroxytetrahydrofuran-2-yl)methyl)-1*H*-1,2,3-triazol-4-yl)methyl)-5-methoxybenzamide (**9b**): Following the general procedure **B**, the title product was obtained as off-white solid (55 mg, 60%) ^1^H-NMR (400 MHz, DMSO-*d*_6_): δ 11.34 (bs, *J* = 2.2 Hz, 1H, NH), 8.79 (t, *J* = 5.7 Hz, 1H, NH), 7.94 (s, 1H, Ar-H), 7.57 (d, *J* = 8.1 Hz, 1H, Ar-H), 7.08 (d, *J* = 2.9 Hz, 1H, Ar-H), 6.83 (dd, *J* = 8.9, 2.8 Hz, 1H, Ar-H), 6.65 (d, *J* = 8.9 Hz, 1H, Ar-H), 6.15 (t, *J* = 6.9 Hz, 1H, CH), 6.03 (s, 2H, NH_2_), 5.62 (dd, *J* = 8.1, 2.0 Hz, 1H, Ar-H), 5.51 (d, *J* = 4.3 Hz, 1H, OH), 4.72–4.64 (m, 1H, CH_2_), 4.58 (dd, *J* = 14.2, 7.7 Hz, 1H, CH_2_), 4.45 (d, *J* = 5.7 Hz, 2H, CH_2_), 4.25–4.23 (m, 1H, CH), 4.10–4.05 (m, 1H, CH), 3.67 (s, 3H, OCH_3_) 2.21–2.10 (m, 2H, CH_2_); ^13^C-NMR (101 MHz, DMSO-*d*_6_): δ 168.56, 163.01, 150.41, 149.33, 145.39, 144.00, 140.74, 123.64, 119.90, 117.84, 114.33, 111.82, 102.10, 84.40, 84.29, 70.78, 55.58, 51.22, 38.08, 34.49; HRMS (ESI) *m/z* calcd. for C_20_H_24_N_7_O_6_ [M + H]^+^ 458.1783, found 458.1781.

2-Amino-*N*-((1-(((*2R,3S,5R*)-5-(2,4-dioxo-3,4-dihydropyrimidin-1(*2H*)-yl)-3-hydroxytetrahydrofuran-2-yl)methyl)-1*H*-1,2,3-triazol-4-yl)methyl)-5-fluorobenzamide (**9c**): Following the general procedure **B**, the title product was obtained as off-white solid (64 mg, 72%); ^1^H-NMR (400 MHz, DMSO-*d*_6_): δ 11.32 (bs, 1H, NH), 8.80 (t, *J* = 5.7, 1H, NH), 7.95 (s, 1H, Ar-H), 7.55 (d, *J* = 8.1 Hz, 1H, Ar-H), 7.36 (dd, *J* = 10.3, 3.1 Hz, 1H, Ar-H), 7.03 (dt, *J* = 8.5, 4.3 Hz, 1H, Ar-H), 6.70 (dd, *J* = 9.1, 5.0 Hz, 1H, Ar-H), 6.31 (s, 2H, NH_2_), 6.14 (t, *J* = 6.9 Hz, 1H, CH), 5.62 (d, *J* = 8.1 Hz, 1H, Ar-H), 5.51 (s, 1H, OH), 4.67 (dd, *J* = 14.2, 4.5 Hz, 1H, CH_2_), 4.57 (dd, *J* = 14.3, 7.7 Hz, 1H, CH_2_), 4.67–4.44 (d, *J* = 5.7 Hz, 2H, CH_2_), 4.28–4.19 (m, 1H, CH), 4.12–4.02 (m, 1H, CH), 2.25–2.04 (m, 2H, CH_2_); ^13^C-NMR (101 MHz, DMSO-*d*_6_): δ 167.81, 163.02, 153.79, 151.53, 150.41, 146.52, 145.18, 140.74, 123.64, 119.43, 119.20, 117.68, 117.62, 113.96, 113.71, 113.48, 102.10, 84.44, 84.27, 70.79, 51.23, 38.10, 34.55; HRMS (ESI) *m/z* calcd. for C_19_H_20_FN_7_O_5_ [M + Na]^+^ 468.1402, found 468.1401.

2-Amino-5-bromo-*N*-((1-(((*2R,3S,5R*)-5-(2,4-dioxo-3,4-dihydropyrimidin-1(*2H*)-yl)-3-hydroxytetrahydrofuran-2-yl)methyl)-1*H*-1,2,3-triazol-4-yl)methyl)benzamide (**9e**): Following the general procedure **B**, the title product was obtained as off-white solid (53 mg, 52%); ^1^H-NMR (400 MHz, DMSO-*d*_6_): δ 11.32 (bs, 1H, NH), 8.87 (t, *J* = 5.7, 1H, NH), 7.95 (s, 1H, Ar-H), 7.67 (d, *J* = 2.4 Hz, 1H, Ar-H), 7.56 (d, *J* = 8.1 Hz, 1H, Ar-H), 7.26 (dd, *J* = 8.8, 2.3 Hz, 1H, Ar-H), 6.67 (d, *J* = 8.9 Hz, 1H, Ar-H), 6.58 (s, 2H, NH_2_), 6.14 (t, *J* = 6.9 Hz, 1H, CH), 5.62 (d, *J* = 8.0 Hz, 1H, Ar-H), 5.50 (d, *J* = 4.3 Hz, 1H, OH), 4.61 (d, *J* = 4.5 Hz, 1H, CH_2_), 4.49 (d, *J* = 7.6 Hz, 1H, CH_2_), 4.43 (d, *J* = 5.6 Hz, 2H, CH_2_), 4.26–4.21 (m, 1H, CH), 4.14–4.02 (m, 1H, CH), 2.23–2.04 (m, 2H, CH_2_); ^13^C-NMR (101 MHz, DMSO-*d*_6_): δ 168.00, 163.45, 150.85, 149.44, 145.57, 141.18, 134.74, 134.71, 130.71, 124.05, 118.94, 116.10, 105.38, 102.55, 84.87, 84.72, 71.24, 38.54, 34.96; HRMS (ESI) *m/z* calcd. for C_19_H_20_BrN_7_O_5_ [M + Na]^+^ 528.0602, found 528.0600.

2-Amino-*N*-((1-(((*2R,3S,5R*)-5-(4-benzamido-2-oxopyrimidin-1(*2H*)-yl)-3-hydroxytetrahydrofuran-2-yl)methyl)-1*H*-1,2,3-triazol-4-yl)methyl)benzamide (**12a**): Following the general procedure **B**, the title product was obtained as off-white solid (65 mg, 61%); ^1^H-NMR (400 MHz, DMSO-*d*_6_): δ 11.24 (bs, 1H, NH), 8.73 (t, 1H, NH), 8.12 (d, *J* = 7.5 Hz, 1H, Ar-H), 8.05–7.98 (m, 3H, Ar-H), 7.62 (t, *J* = 7.4 Hz, 1H, Ar-H), 7.56–7.46 (m, 3H, Ar-H), 7.37 (s, 1H, Ar-H), 7.13–7.07 (m, 1H, Ar-H), 6.67 (dd, *J* = 8.2, 1.2 Hz, 1H, Ar-H), 6.48 (t, *J* = 1.0 Hz, 1H, Ar-H), 6.41 (s, 2H, NH_2_), 6.16 (t, *J* = 6.5 Hz, 1H, CH), 5.54 (d, *J* = 4.2 Hz, 1H, OH), 4.76–4.61 (m, 2H, CH_2_), 4.46 (d, *J* = 5.7 Hz, 2H, CH_2_), 4.30–4.22 (m, 1H, CH), 4.22–4.14 (m, 1H, CH), 2.37–2.09 (m, 2H, CH_2_); ^13^C-NMR (151 MHz, DMSO-*d*_6_): δ 168.82, 149.76, 145.52, 132.76, 131.77, 128.46, 128.13, 123.66, 116.35, 114.52, 114.19, 87.18, 84.94, 70.77, 51.22, 34.47; HRMS (ESI) *m/z* calcd. for C_26_H_26_N_8_O_5_ [M + H]^+^ 531.2099, found 531.2098.

2-Amino-*N*-((1-(((*2R,3S,5R*)-5-(4-benzamido-2-oxopyrimidin-1(*2H*)-yl)-3-hydroxytetrahydrofuran-2-yl)methyl)-1H-1,2,3-triazol-4-yl)methyl)-5-methoxybenzamide (**12b**): Following the general procedure **B**, the title product was obtained as light brown solid (70 mg, 62%); ^1^H-NMR (400 MHz, DMSO-*d*_6_): δ 11.25 (bs, 1H, NH), 8.79 (t, *J* = 5.7 Hz, 1H, NH), 8.13 (d, *J* = 7.6 Hz, 1H, Ar-H), 8.06–7.96 (m, 3H, Ar-H), 7.68–7.59 (m, 1H, Ar-H), 7.53 (t, *J* = 8.3, 7.0 Hz, 2H, Ar-H), 7.38 (s, 1H, Ar-H), 7.09 (d, *J* = 2.9 Hz, 1H, Ar-H), 6.82 (dd, *J* = 8.9, 2.8 Hz, 1H, Ar-H), 6.65 (d, *J* = 8.9 Hz, 1H, Ar-H), 6.16 (t, *J* = 6.5 Hz, 1H, CH), 6.00 (s, 2H, NH_2_), 5.56 (d, *J* = 4.3 Hz, 1H, OH), 4.78–4.62 (m, 2H, CH_2_), 4.47 (d, *J* = 5.7 Hz, 2H, CH_2_), 4.34–4.23 (m, 1H, CH), 4.24–4.12 (m, 1H, CH), 3.66 (s, 3H, CH_3_) 2.38–2.09 (m, 2H, CH_2_); ^13^C-NMR (151 MHz, DMSO-*d*_6_): δ 168.56, 149.31, 145.46, 144.04, 132.78, 128.50, 128.47, 123.71, 119.89, 117.81, 114.32, 111.81, 86.53, 84.95, 70.79, 55.56, 51.26, 34.49; HRMS (ESI) *m/z* calcd. for C_27_H_29_N_8_O_6_ [M + H]^+^ 561.2205, found 561.2204.

2-Amino-*N*-((1-(((*2R,3S,5R*)-5-(4-benzamido-2-oxopyrimidin-1(*2H*)-yl)-3-hydroxytetrahydrofuran-2-yl)methyl)-1*H*-1,2,3-triazol-4-yl)methyl)-5-fluorobenzamide (**12c**): Following the general procedure **B**, the title product was obtained as light yellowish solid (80 mg, 73%); ^1^H-NMR (400 MHz, DMSO-*d*_6_): δ 11.24 (bs, 1H, NH), 8.81 (t, *J* = 5.7 Hz, 1H, NH), 8.12 (d, *J* = 7.5 Hz, 1H, Ar-H), 8.05–7.98 (m, 3H, Ar-H), 7.67–7.59 (m, 1H, Ar-H), 7.52 (t, *J* = 7.7 Hz, 2H, Ar-H), 7.36 (dd, *J* = 10.3, 3.0 Hz, 2H, Ar-H), 7.06–6.92 (m, 1H, Ar-H), 6.69 (dd, *J* = 9.0, 5.0 Hz, 1H, Ar-H), 6.31 (s, 2H, NH_2_), 6.16 (t, *J* = 6.5 Hz, 1H, CH), 5.54 (d, *J* = 4.3 Hz, 1H, OH), 4.69 (dd, *J* = 8.9, 6.1 Hz, 2H, CH_2_), 4.46 (d, *J* = 5.6 Hz, 2H, CH_2_), 4.30–4.23 (m, 1H, CH), 4.26–4.15 (m, 1H, CH), 2.38–2.10 (m, 1H, CH_2_); ^13^C-NMR (101 MHz, DMSO-*d*_6_): δ 167.85, 153.71, 151.86, 148.73, 146.50, 145.22, 132.74, 128.43, 123.68, 119.48, 119.14, 117.61, 113.67, 113.45, 86.76, 84.92, 70.77, 50.80, 34.52; HRMS (ESI) *m/z* calcd. for C_25_H_26_FN_8_O_5_ [M + Na]^+^ 571.1824, found 571.1823.

2-Amino-*N*-((1-(((*2R,3S,5R*)-5-(4-benzamido-2-oxopyrimidin-1(*2H*)-yl)-3-hydroxytetrahydrofuran-2-yl)methyl)-1*H*-1,2,3-triazol-4-yl)methyl)-5-chlorobenzamide (**12d**): Following the general procedure **B**, the title product was obtained as off-white solid (70 mg, 62%); ^1^H-NMR (400 MHz, DMSO-*d*_6_): δ 11.22 (bs, 1H, NH), 8.90 (t, 1H, NH), 8.12 (d, *J* = 7.3 Hz, 1H, Ar-H), 8.06–7.94 (m, 4H, Ar-H), 7.62 (t, 1H, Ar-H), 7.57 (d, *J* = 2.5 Hz, 1H, Ar-H), 7.52 (t, *J* = 7.7 Hz, 2H, Ar-H), 7.13 (dd, *J* = 8.8, 2.5 Hz, 1H, Ar-H), 6.71 (d, *J* = 8.8 Hz, 1H, Ar-H), 6.56 (s, 2H, NH_2_,), 6.15 (t, 1H, CH), 5.63 (d, *J* = 4.3 Hz, 1H, OH), 4.78–4.62 (m, 2H, CH_2_), 4.45 (d, *J* = 5.6 Hz, 2H, CH_2_), 4.30–4.22 (m, 1H, CH), 4.26–4.15 (m, 1H, CH), 2.41–2.10 (m, 1H, CH_2_); ^13^C-NMR (151 MHz, DMSO-*d*_6_): δ 167.63, 148.69, 145.20, 132.78, 131.53, 128.50, 128.47, 127.47, 123.72, 118.06, 117.72, 114.97, 96.44, 84.93, 70.73, 51.26, 34.52; HRMS (ESI) *m/z* calcd. for C_26_H_26_ClN_8_O_5_ [M + H]^+^ 565.1709, found 565.1709.

2-Amino-*N*-((1-(((*2R,3S,5R*)-5-(4-benzamido-2-oxopyrimidin-1(*2H*)-yl)-3-hydroxytetrahydrofuran-2-yl)methyl)-1*H*-1,2,3-triazol-4-yl)methyl)-5-bromobenzamide (**12e**): Following the general procedure **B**, the title product was obtained as off-white solid (65 mg, 53%); ^1^H-NMR (600 MHz, DMSO-*d*_6_): δ 11.26 (bs, 1H, NH), 8.89 (t, *J* = 5.9 Hz, 1H, NH), 8.12 (d, *J* = 7.6 Hz, 1H, Ar-H), 8.06–7.98 (m, 3H, Ar-H), 7.68 (d, *J* = 2.4 Hz, 1H, Ar-H), 7.63 (t, *J* = 7.3 Hz, 1H, Ar-H), 7.52 (t, *J* = 7.7 Hz, 2H, Ar-H), 7.40–7.34(m, 1H, Ar-H) 7.24 (dd, *J* = 8.8, 2.4 Hz, 1H, Ar-H), 6.67 (d, *J* = 8.8 Hz, 1H, Ar-H), 6.59 (s, 2H, NH_2_), 6.17 (t, *J* = 6.5 Hz, 1H, CH), 5.56 (d, *J* = 4.3 Hz, 1H, OH), 4.81–4.64 (m, 2H CH_2_), 4.45 (d, *J* = 5.8 Hz, 2H, CH_2_), 4.29–4.24 (m, 1H, CH), 4.24–4.17 (m, 1H, CH), 2.32–2.02 (m, 2H, CH_2_); ^13^C-NMR (151 MHz, DMSO-*d*_6_): δ 167.98, 149.46, 145.65, 134.69, 130.72, 128.95, 128.90, 124.14, 118.92, 116.06, 105.36, 85.39, 79.66, 79.44, 79.22, 71.24, 51.72, 34.97; HRMS (ESI) *m/z* calcd. for C_26_H_25_BrN_8_O_5_ [M + Na]^+^ 631.1023, found 631.1022.

General procedure (**C**) for the deprotection of benzoyl group: To a solution of *N*-benzoyl triazole (0.1 mmol) in methanol (6 mL), Aq. NH_3_ (28%) (6 mL) was added. The resulting mixture was stirred at rt for 16 h. After completion of the reaction, solvent was evaporated in vacuo*,* crude was purified by dissolving in methanol (1 mL), then, excess diethyl ether was added, and solid precipitate was collected and dried, which gives desired compound as off-white to light brown solid between (81–92% yields).

2-Amino-*N*-((1-(((*2R,3S,5R*)-5-(4-amino-2-oxopyrimidin-1(*2H*)-yl)-3-hydroxytetrahydrofuran-2-yl)methyl)-1*H*-1,2,3-triazol-4-yl)methyl)benzamide (**13a**): Following the general procedure **C**, the title product was obtained as off-white solid (38 mg, 88%); ^1^H-NMR (600 MHz, DMSO-*d*_6_): δ 8.73 (t, *J* = 5.8, 1H, NH), 7.94 (s, 1H, Ar-H), 7.52 (d, *J* = 7.5 Hz, 1H, Ar-H), 7.50 (dd, *J* = 8.0, 1.5 Hz, 1H, Ar-H), 7.14 (s, 2H, NH_2_), 7.13 (td, *J* = 8.4, 7.0, 1.5 Hz, 1H, Ar-H), 6.69 (dd, *J* = 8.2, 1.2 Hz, 1H, Ar-H), 6.50 (td, *J* = 8.1, 7.0, 1.2 Hz, 1H, Ar-H), 6.42 (s, 2H, NH_2_), 6.18 (t, *J* = 7.5, 6.2 Hz, 1H, CH), 5.75 (d, *J* = 7.4, 1H, Ar-H), 5.45 (d, *J* = 4.3 Hz, 1H, OH), 4.66 (dd, *J* = 14.3, 4.4 Hz, 1H, CH_2_), 4.58 (dd, *J* = 14.2, 7.7 Hz, 1H, CH_2_), 4.45 (d, *J* = 5.7 Hz, 2H, CH_2_), 4.22–4.21 (m, 3.6 Hz, 1H, CH), 4.09–4.06 (m, 4.2 Hz, 1H, CH), 2.14–2.08 (m, 1H, CH_2_), 2.05–1.98 (m, 1H, CH_2_); ^13^C-NMR (101 MHz, DMSO-*d*_6_): δ 168.84, 165.55, 154.99, 149.76, 145.42, 141.12, 131.83, 128.17, 123.60, 116.39, 114.60, 114.25, 94.41, 85.17, 84.17, 70.98, 51.38, 39.55, 34.48. HRMS (ESI) *m/z* calcd. for C_19_H_22_N_8_O_4_ [M + H]^+^ 449.1656, found 449.1655.

2-Amino-*N*-((1-(((*2R,3S,5R*)-5-(4-amino-2-oxopyrimidin-1(*2H*)-yl)-3-hydroxytetrahydrofuran-2-yl)methyl)-1*H*-1,2,3-triazol-4-yl)methyl)-5-methoxybenzamide (**13b**): Following the general procedure **C**, the title product was obtained as light brown solid (37 mg, 81%); ^1^H-NMR (400 MHz, DMSO-*d*_6_): δ 8.77 (t, *J* = 5.7 Hz, 1H, NH), 7.95 (s, 1H, Ar-H), 7.53 (d, *J* = 7.4 Hz, 1H, Ar-H), 7.28–7.12 (m, 2H, NH_2_), 7.09 (d, *J* = 2.9 Hz, 1H, Ar-H), 6.84 (dd, *J* = 8.9, 2.9 Hz, 1H, Ar-H), 6.66 (d, *J* = 8.9 Hz, 1H), 6.18 (t, *J* = 6.8 Hz, 1H, CH), 6.01 (s, 2H, NH_2_), 5.75 (d, *J* = 7.4 Hz, 1H, Ar-H), 5.47 (d, *J* = 4.2 Hz, 1H, OH), 4.62 (dd, *J* = 21.4, 6.1 Hz, 2H, CH_2_), 4.46 (d, *J* = 5.6 Hz, 2H, CH_2_), 4.23–4.22 (m, 1H, CH), 4.12–4.04 (m, 1H, CH), 3.67 (s, 3H, OCH_3_), 2.23–1.96 (m, 2H, CH_2_); ^13^C-NMR (101 MHz, DMSO-*d*_6_): δ 169.00, 165.99, 155.42, 149.78, 145.78, 144.47, 141.57, 124.08, 120.35, 118.27, 114.83, 112.30, 94.83, 85.62, 84.61, 71.43, 56.04, 51.84, 34.94; HRMS (ESI) *m/z* calcd. for C_20_H_24_N_8_O_5_ [M + Na]^+^ 479.1762, found 479.1760.

2-Amino-*N*-((1-(((*2R,3S,5R*)-5-(4-amino-2-oxopyrimidin-1(*2H*)-yl)-3-hydroxytetrahydrofuran-2-yl)methyl)-1*H*-1,2,3-triazol-4-yl)methyl)-5-fluorobenzamide (**13c**): Following the general procedure **C**, the title product was obtained as off-white solid (41 mg, 92%); ^1^H-NMR (400 MHz, DMSO-*d*_6_): δ 8.82 (t, *J* = 5.6 Hz, 1H, NH), 7.97 (s, 1H, Ar-H), 7.52 (d, *J* = 7.5 Hz, 1H, Ar-H), 7.37 (dd, *J* = 10.3, 3.0 Hz, 1H, Ar-H), 7.17 (d, *J* = 13.1 Hz, 2H, NH_2_), 7.09–7.00 (m, 1H, Ar-H), 6.70 (dd, *J* = 9.0, 5.0 Hz, 1H, Ar-H), 6.33 (s, 2H, NH_2_), 6.17 (t, 1H, *J* = 6.8 Hz, CH), 5.73 (d, *J* = 7.4 Hz, 1H, Ar-H), 5.45 (d, *J* = 4.3 Hz, 1H, OH), 4.75–4.53 (m, 2H, CH_2_), 4.44 (d, *J* = 5.7 Hz, 2H, CH_2_) 4.24–4.16 (m, 1H, CH), 4.11–4.03 (m, 1H, CH), 2.16–1.95 (m, 2H, CH_2_); ^13^C-NMR (101 MHz, DMSO-*d*_6_): δ 167.78, 165.54, 154.98, 153.80, 151.48, 146.51, 145.13, 141.13, 123.62, 119.42, 119.19, 117.66, 117.58, 113.96, 113.90, 113.72, 113.50, 94.39, 85.19, 84.15, 70.98, 51.40, 34.52; HRMS (ESI) *m/z* calcd. for C_19_H_22_FN_8_O_4_ [M + Na]^+^ 567.1562, found 567.1564.

2-Amino-*N*-((1-(((*2R,3S,5R*)-5-(4-amino-2-oxopyrimidin-1(*2H*)-yl)-3-hydroxytetrahydrofuran-2-yl)methyl)-1*H*-1,2,3-triazol-4-yl)methyl)-5-chlorobenzamide (**13d**): Following the general procedure **C**, the title product was obtained as off-white solid (38 mg, 82%); ^1^H-NMR (600 MHz, DMSO-*d*_6_): δ 8.88 (t, *J* = 5.7 Hz, 1H, NH), 7.96 (s, 1H, Ar-H), 7.57 (d, *J* = 2.5 Hz, 1H, Ar-H), 7.52 (d, *J* = 7.4 Hz, 1H, Ar-H), 7.26–6.99 (m, 3H, NH_2_, Ar-H), 6.72 (d, *J* = 8.8 Hz, 1H, Ar-H), 6.57 (s, 2H, NH_2_), 6.17 (t, *J* = 7.5, 6.2 Hz, 1H, CH), 5.74 (d, *J* = 7.4 Hz, 1H, Ar-H), 5.46 (d, *J* = 4.0 Hz, 1H, OH), 4.66 (dd, *J* = 14.2, 4.4 Hz, 1H, CH_2_), 4.57 (dd, *J* = 14.2, 7.7 Hz, 1H, CH_2_), 4.44 (d, *J* = 5.6 Hz, 2H, CH_2_), 4.25–4.19 (m, 1H, CH), 4.08–4.05 (m, 1H, CH), 2.12–2.07 (m, 1H, CH_2_), 2.03–1.99 (m, 1H, CH_2_); ^13^C-NMR (151 MHz, DMSO-*d*_6_): δ 168.06, 165.98, 155.41, 149.12, 145.53, 141.57, 132.02, 127.92, 124.06, 118.51, 118.19, 115.45, 108.11, 94.83, 85.61, 84.60, 71.42, 51.85, 34.96; HRMS (ESI) *m/z* calcd. for C_19_H_21_ClN_8_O_4_ [M + H]^+^ 483.1267, found 483.1264.

2-Amino-*N*-((1-(((*2R,3S,5R*)-5-(4-amino-2-oxopyrimidin-1(*2H*)-yl)-3-hydroxytetrahydrofuran-2-yl)methyl)-1*H*-1,2,3-triazol-4-yl)methyl)-5-bromobenzamide (**13e**): Following the general procedure C, the title product was obtained as off-white solid (43 mg, 85%); ^1^H-NMR (400 MHz, DMSO-*d*_6_): δ 8.87 (t, *J* = 5.5 Hz, 1H, NH), 7.96 (s, 1H, Ar-H), 7.68 (d, *J* = 2.4 Hz, 1H, Ar-H), 7.52 (d, *J* = 7.4 Hz, 1H, Ar-H), 7.26 (dd, *J* = 8.9, 2.3 Hz, 1H, Ar-H), 7.17 (s, 2H, NH_2_), 6.67 (d, *J* = 8.8 Hz, 1H, Ar-H), 6.58 (s, 2H, NH_2_), 6.17 (t, *J* = 6.8 Hz, 1H, CH), 5.76 (s, 1H, Ar-H), 5.45 (d, *J* = 4.3 Hz, 1H, OH), 4.65– 4.58 (m, 2H, CH_2_), 4.43 (d, *J* = 5.5 Hz, 2H, CH_2_), 4.24–4.16 (m, 1H, CH), 4.11–4.03 (m, 1H, CH), 2.10–1.95 (m, 2H, CH_2_); ^13^C-NMR (151 MHz, DMSO-*d*_6_): δ 167.52, 165.54, 148.99, 145.07, 141.12, 134.26, 130.27, 123.61, 118.47, 115.62, 104.92, 85.18, 84.15, 70.97, 69.79, 51.39, 34.50; HRMS (ESI) *m/z* calcd. for C_19_H_21_BrN_8_O_4_ [M + H]^+^ 505.0942, found 505.0942.

### 4.2. Biological Activity

For the *pqs:gfp* reporter assay, the PAO1 *pqsA-gfp P. aeruginosa* strain carries the PqsR-regulated pqsA promoter fused to *gfp* [35]. The compounds were dissolved in DMSO at a concentration of 20 mg/mL (approximately 40 mM based on molecular weights). The assays with pqsA reporters were carried out in MHB (Mueller Hinton Broth) in 96-well plates, at 37 °C with intermittent shaking. Readings were taken every 15 min for both OD600 and fluorescence; the fluorescence values shown in the graph were normalized with respect to OD600. All the reporters tested peak in promoter activity after 6–8 h growth. At intervals of 15 min, both GFP fluorescence and OD600 were recorded. Negative control refers to medium containing the same concentration of DMSO as the highest concentration of the test compound. 

For microscopy imaging to evaluate adhesion and biofilm formation, planktonic cultures of PAO1 were grown for 24 h followed by re-suspension in TSB media when a bacterial density of OD600 = 0.10 ± 0.02 was reached. Then, 600 µL of the re-suspended cells was added to 12-well plates (Corning Corp. USA), and then, incubated at 37 °C and 100 rpm to initiate bacterial adhesion and biofilm growth. Samples contained either compounds at 400 μM, or TSB medium or DMSO as negative controls. After 24 h, wells were washed twice with 1×PBS to remove any loosely adhered bacteria and imaged using phase contrast microscopy (Zeiss, Axio, Germany), for bacterial adhesion, aggregation and biofilm formation as described [36]. 

For pyocyanin quantification, planktonic cultures of PAO1 were grown in 12-wells plate for 24 h in presence of plain TSB, solvent (DMSO) and compounds 400 µM. After growth, the planktonic cultures were transferred into sterile 2 mL Eppendorf tubes and centrifuged at 5000 g for 10 min at 21°C. After centrifugation, the supernatant was removed by pipetting and transferred into new Eppendorf tubes, followed by re-centrifuging at 5000× *g* for 10 min at 21 °C. The supernatants were filtered using 0.4 µM filters and added to 96-well plates, and pyocyanin concentration was determined by measuring absorbance at OD695 using a plate reader (Tecan infinite M1000 pro, Sydney, Australia).

## 5. Conclusions

Inhibition of the PQS quorum sensing system is an attractive target for the development of alternative therapies against multidrug-resistant *P. aeruginosa*. Anthraniloyl-AMP mimics are competitive antagonists of the enzyme PqsA, and block production of virulence factors PQS and HHQ without affecting bacterial growth. In this study, we have designed and synthesized 14 novel anthraniloyl-AMP mimics containing a triazole linker as potential inhibitors of *Pseudomonas* quinolone biosynthesis. However, most of these analogues did not show PQS inhibitory activity against a *P. aeruginosa* reporter chain except for deoxycytidine analogue (**13e**), which inhibited PQS activity by 30% at 125 µM, possibly due to limited cell penetration. Interestingly, these compounds induced significant morphological changes in biofilms as observed by microscopy, suggesting that they could inhibit PQS or other signaling systems responsible for bacterial aggregation. Our future efforts will focus on modifying these analogues to increase bacterial cell penetration.

## Figures and Tables

**Table 1:** PQS inhibition by synthesized compounds using PAO1 *pqsA:gfp* reporter-based assay.

	Compound Name	Biological Activity (Inhibition of *pqsA:gfp*)	Inhibition (%)
1	**7a**	No inhibition	-
2	**7b**	No inhibition	-
3	**7c**	No inhibition	-
4	**7d**	No inhibition	-
5	**7e**	No inhibition	-
6	**9a**	No inhibition	-
7	**9b**	No inhibition	-
8	**9c**	No inhibition	-
9	**9e**	No inhibition	-
10	**13a**	No inhibition	-
11	**13b**	No inhibition	-
12	**13c**	No inhibition	-
13	**13d**	No inhibition	-
14	**13e**	Inhibition	30

## Supplementary Materials

The ^1^H- and ^13^C-NMR spectra for all the new compounds and growth inhibition data are provided in the supplementary file.
