# Supplementary material for: Design, Synthesis and Biological Evaluation of Novel Anthraniloyl-AMP Mimics as PQS Biosynthesis Inhibitors Against Pseudomonas aeruginosa Resistance
Source: Molecules. 2020 Jul 7;25(13):3103. doi: 10.3390/molecules25133103 (PMC7412332; doi:10.3390/molecules25133103)
Supplement: Supplementary file 1 [file molecules-25-03103-s001.pdf]

Design, Synthesis and Biological Evaluation of Novel Anthraniloyl-AMP Mimics as PQS Biosynthesis Inhibitors Against *Pseudomonas aeruginosa* Resistance

Shekh Sabir[1], Sujatha Subramoni[2], Theerthankar Das[2], David StC Black[1], Scott Rice[2], Naresh Kumar[1]*

[1] School of Chemistry, Faculty of Science, The University of New South Wales, Sydney, NSW, Australia

[2] Singapore Centre for Environmental Life Sciences Engineering (SCELSE), Nanyang Technological University, Singapore.

[3] Department of Infectious Diseases and Immunology, School of Medical Sciences, The University of Sydney, Sydney, Australia.

Contents

^{1}H and ^{13}C NMR Spectra of New Compounds S2-S45

2D NMR Spectra of Compound 13a …... ………………………………………….………S46-S49

NMR Signal Assignments of Selected Compounds……………………………………………………………………………S50-S55

PQS Inhibition Activity Data of the Compounds……………………………………………………….………………… S56-S58

Growth Inhibition Data of New Compounds S58-S60

^{1}H NMR spectrum of compound 3b

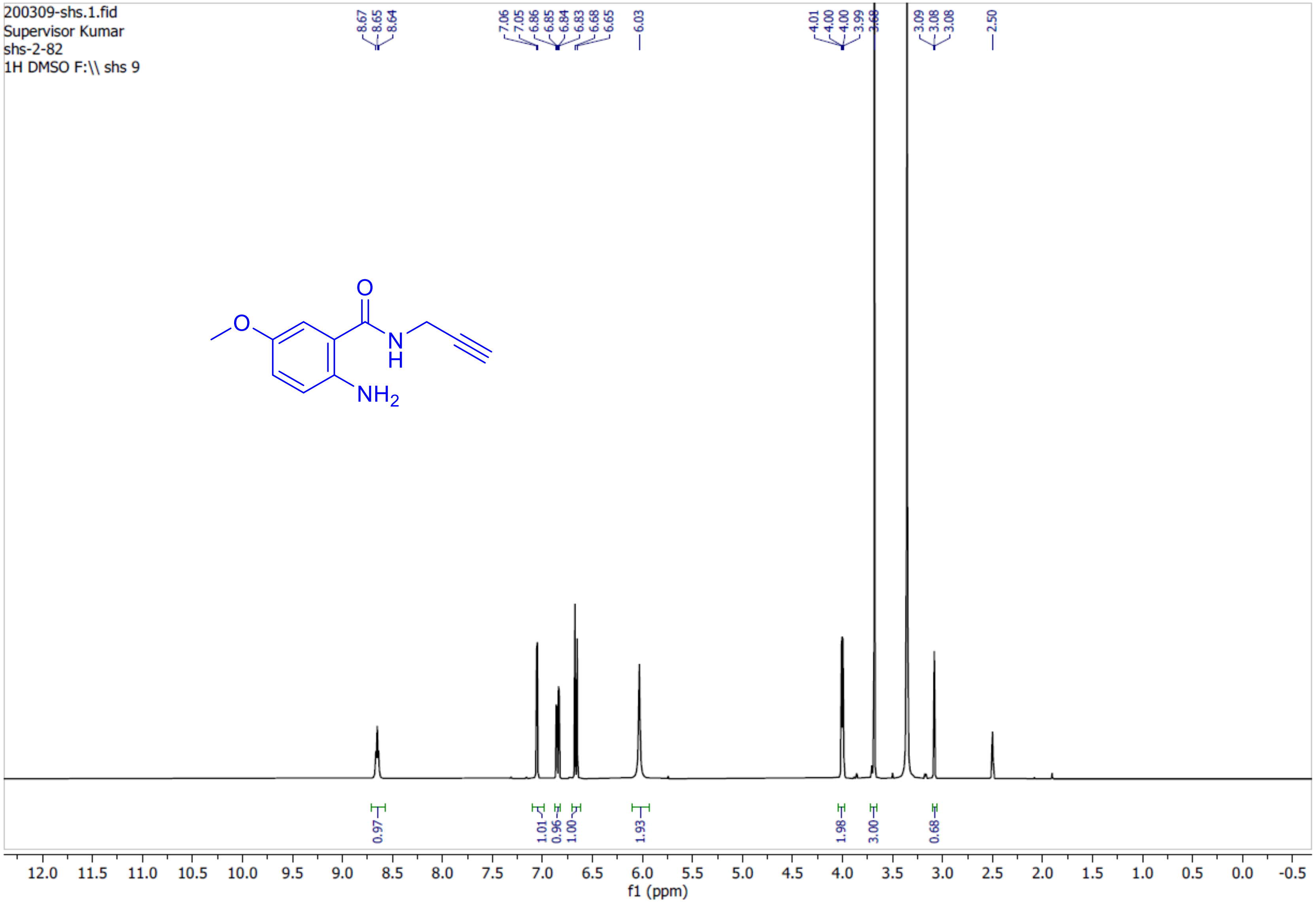

^{13}C NMR spectrum of compound 3b

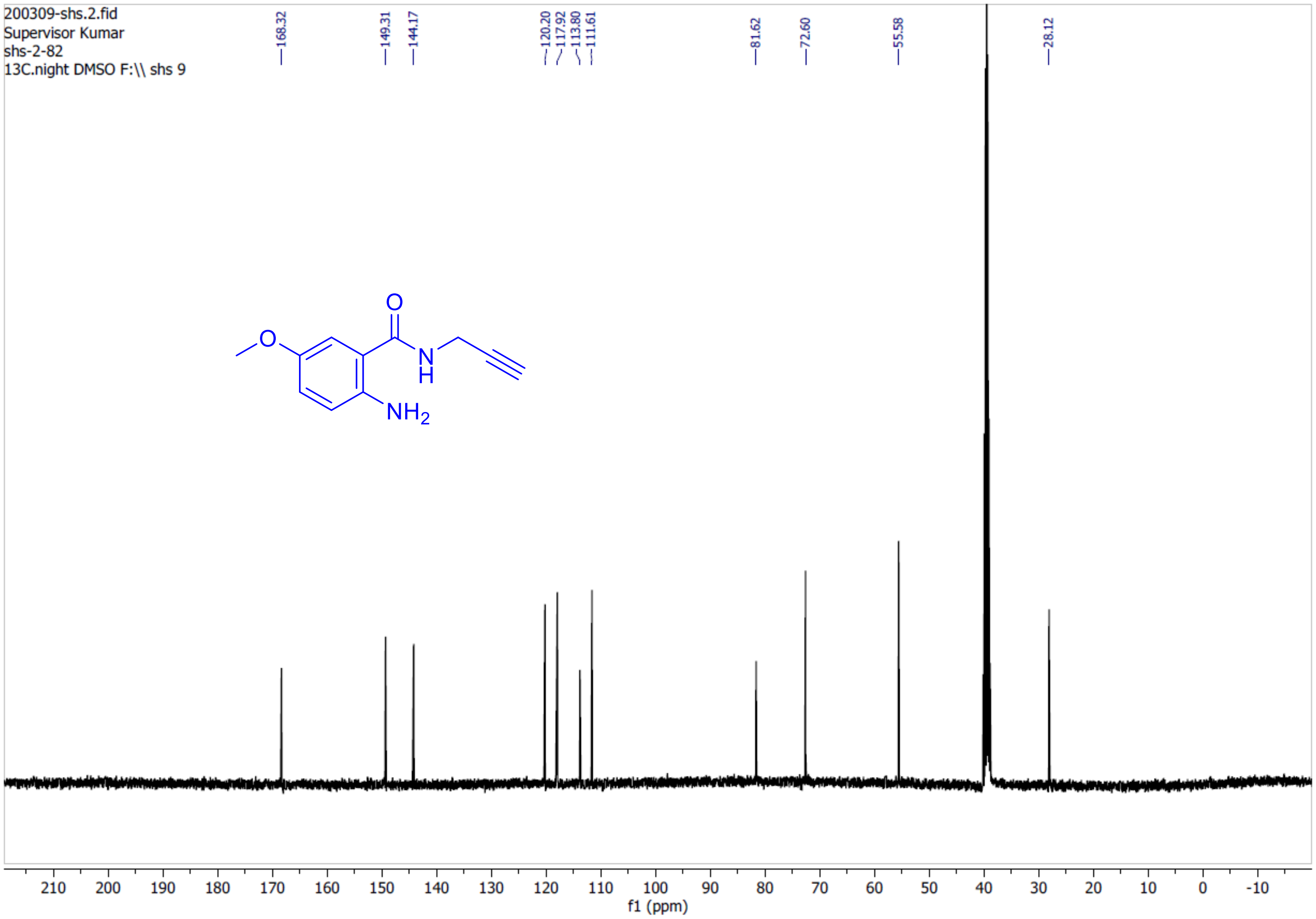

^{1}H NMR spectrum of compound 3c

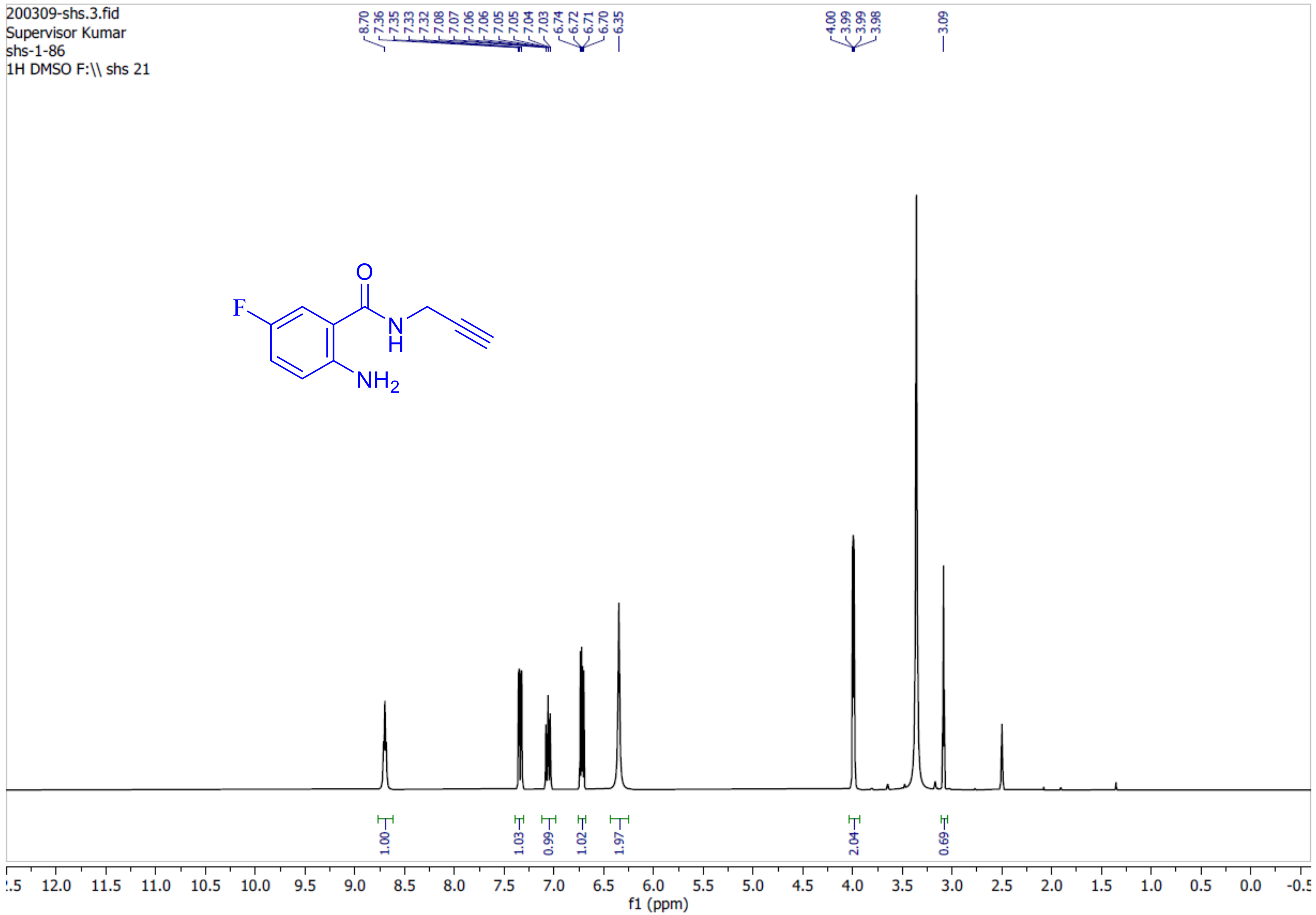

^{13}C NMR spectrum of compound 3c

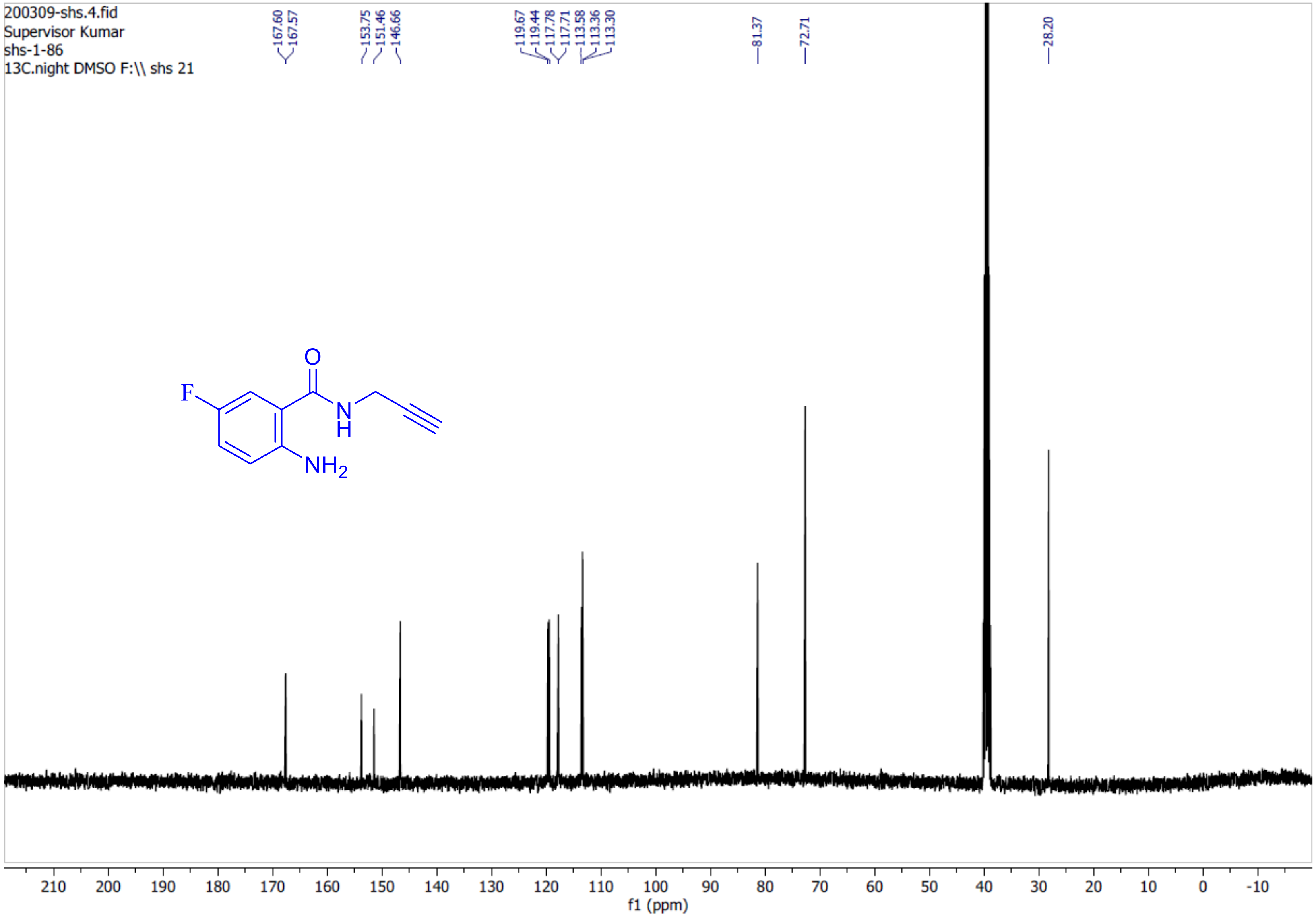

^{1}H NMR spectrum of compound 3d

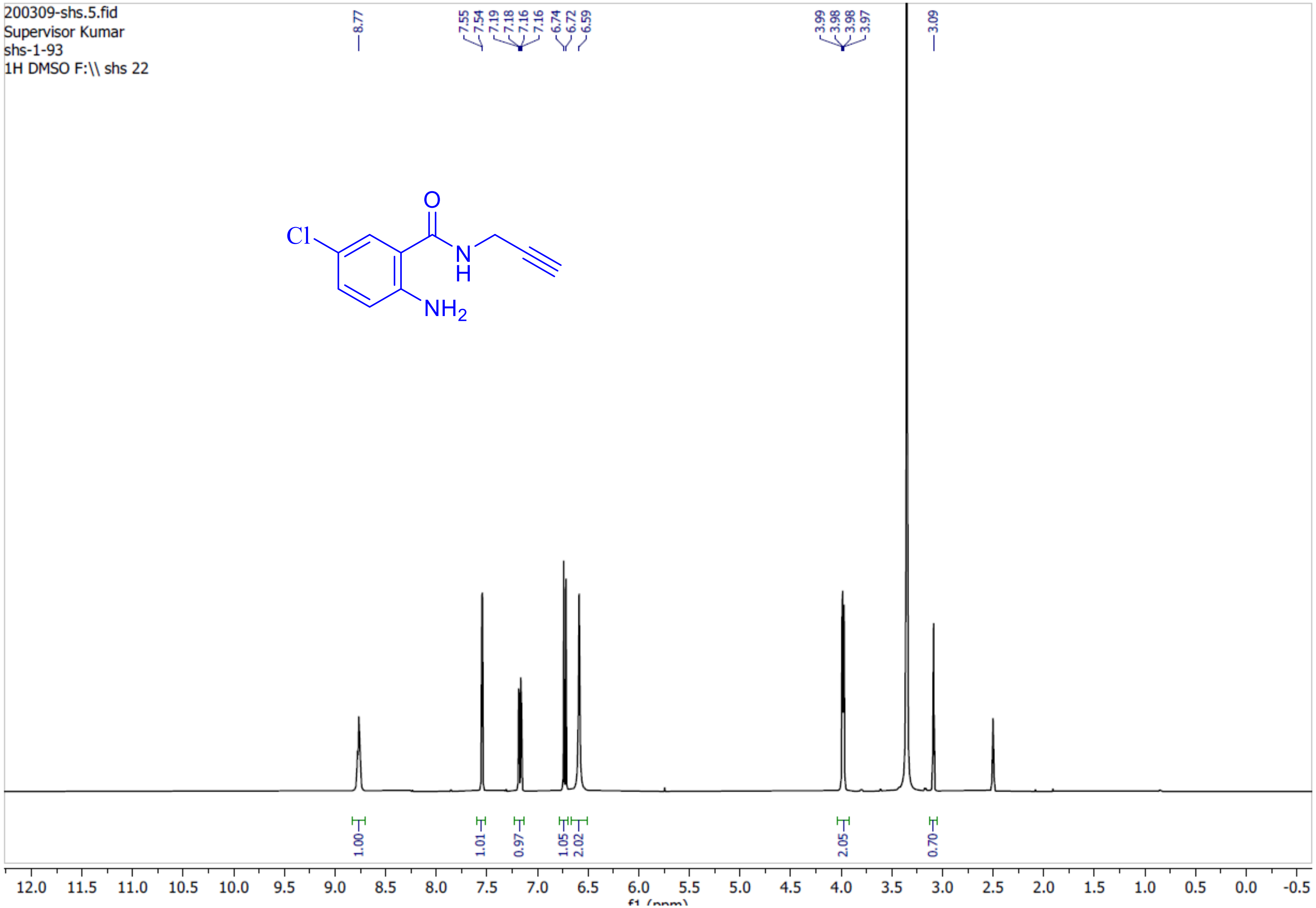

^{13}C NMR spectrum of compound 3d

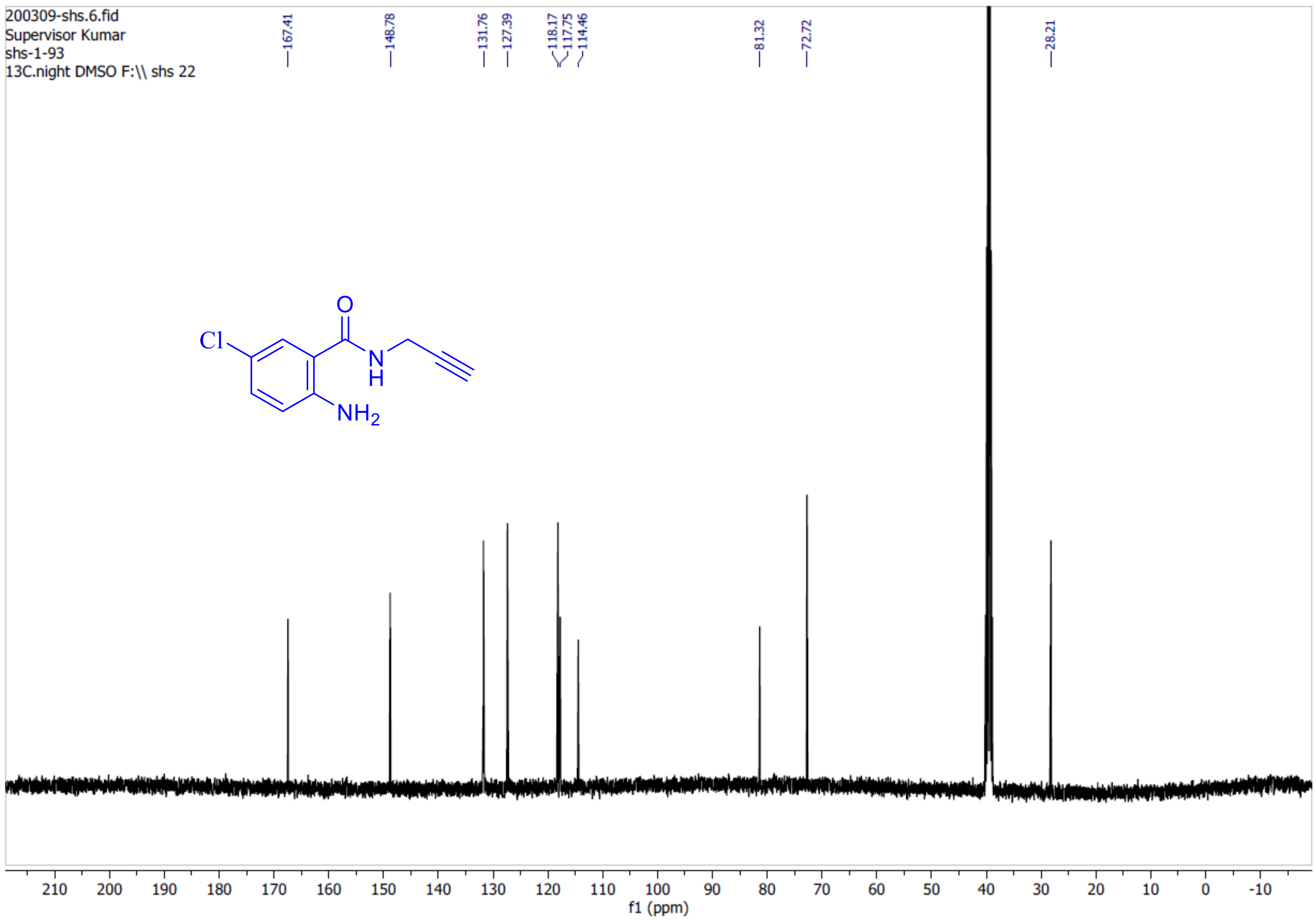

^{1}H NMR spectrum of compound 7a

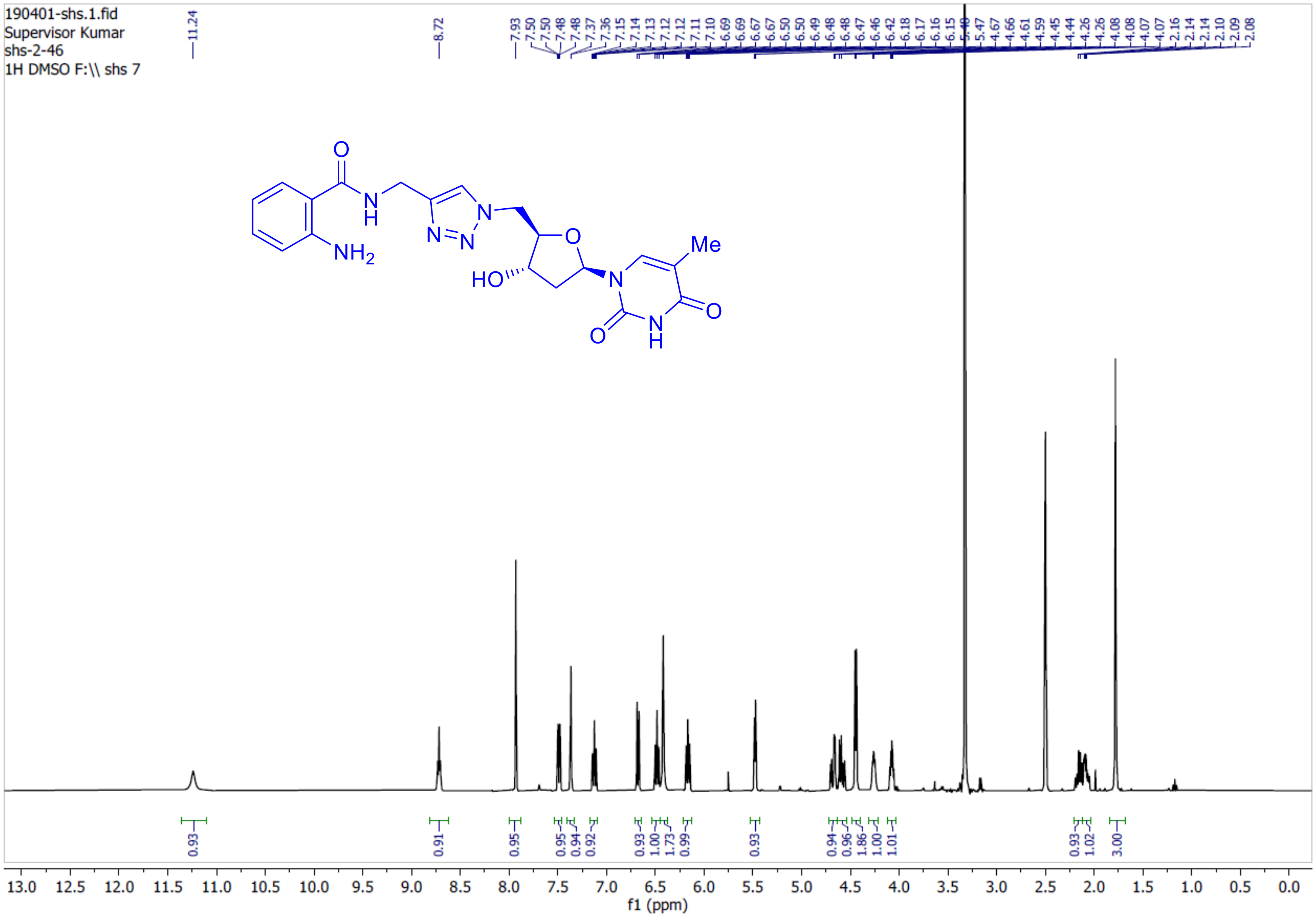

^{13}C NMR spectrum of compound 7a

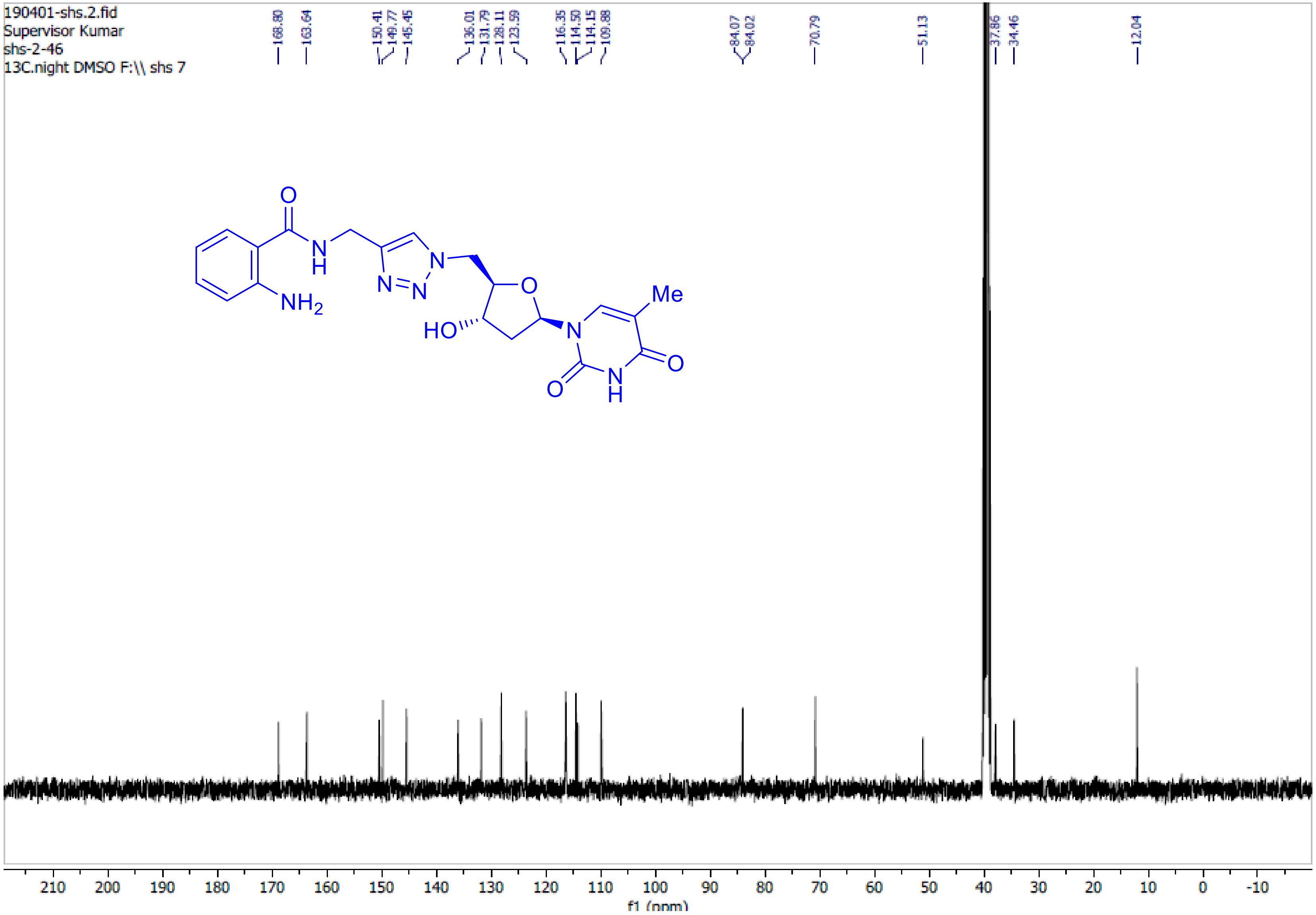

^{1}H NMR spectrum of compound 7b

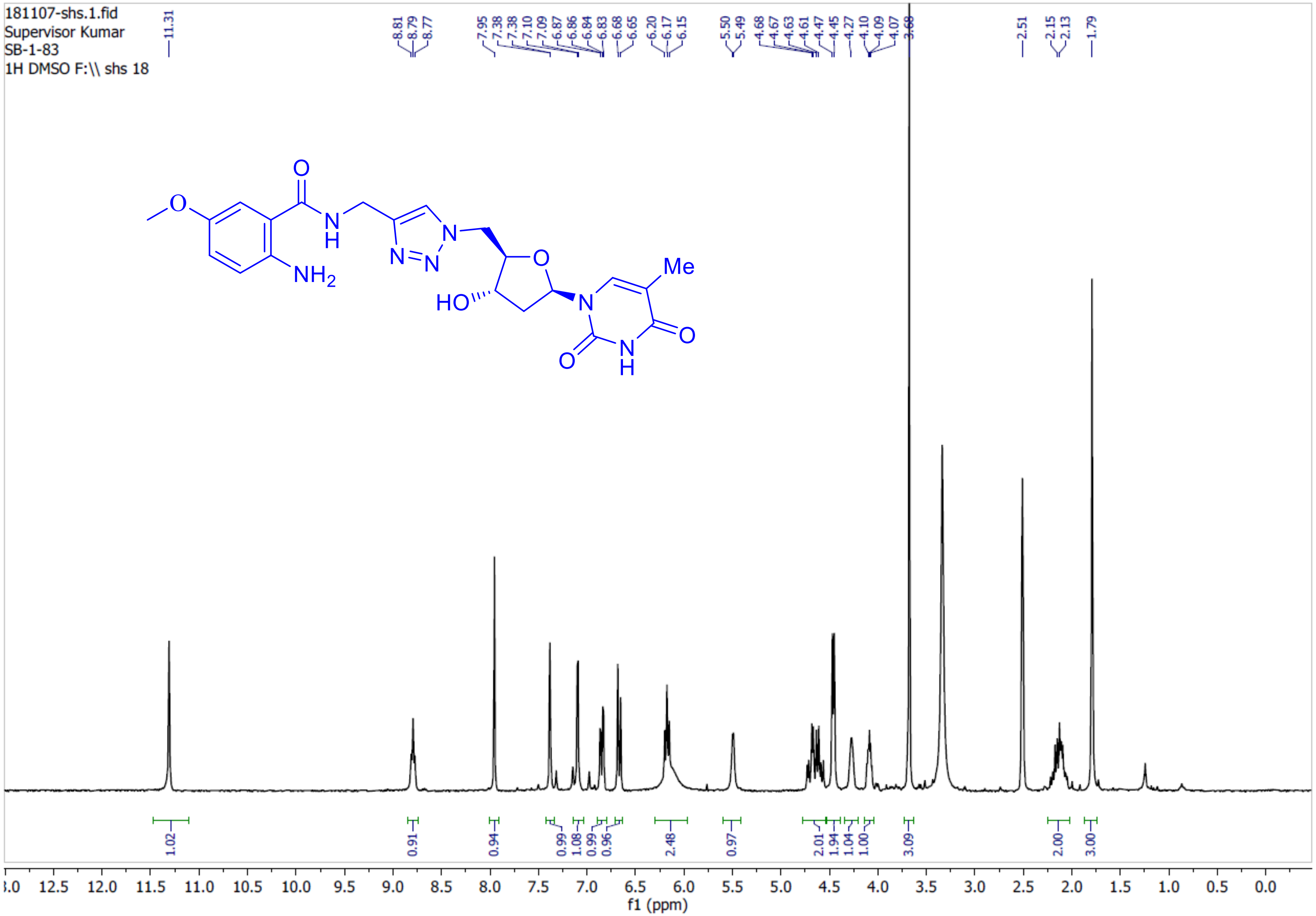

^{13}C NMR spectrum of compound 7b

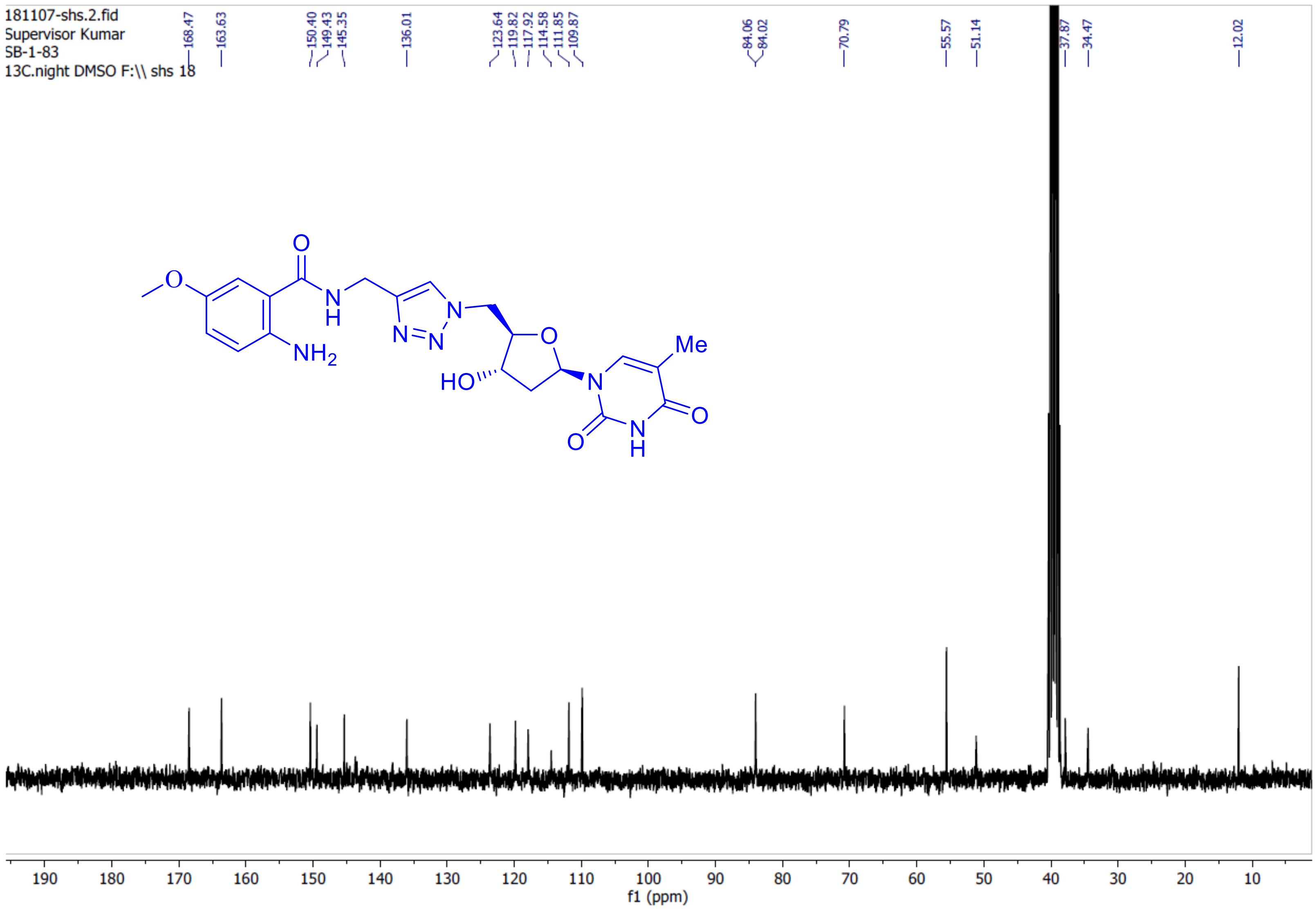

1H NMR spectrum of compound 7c

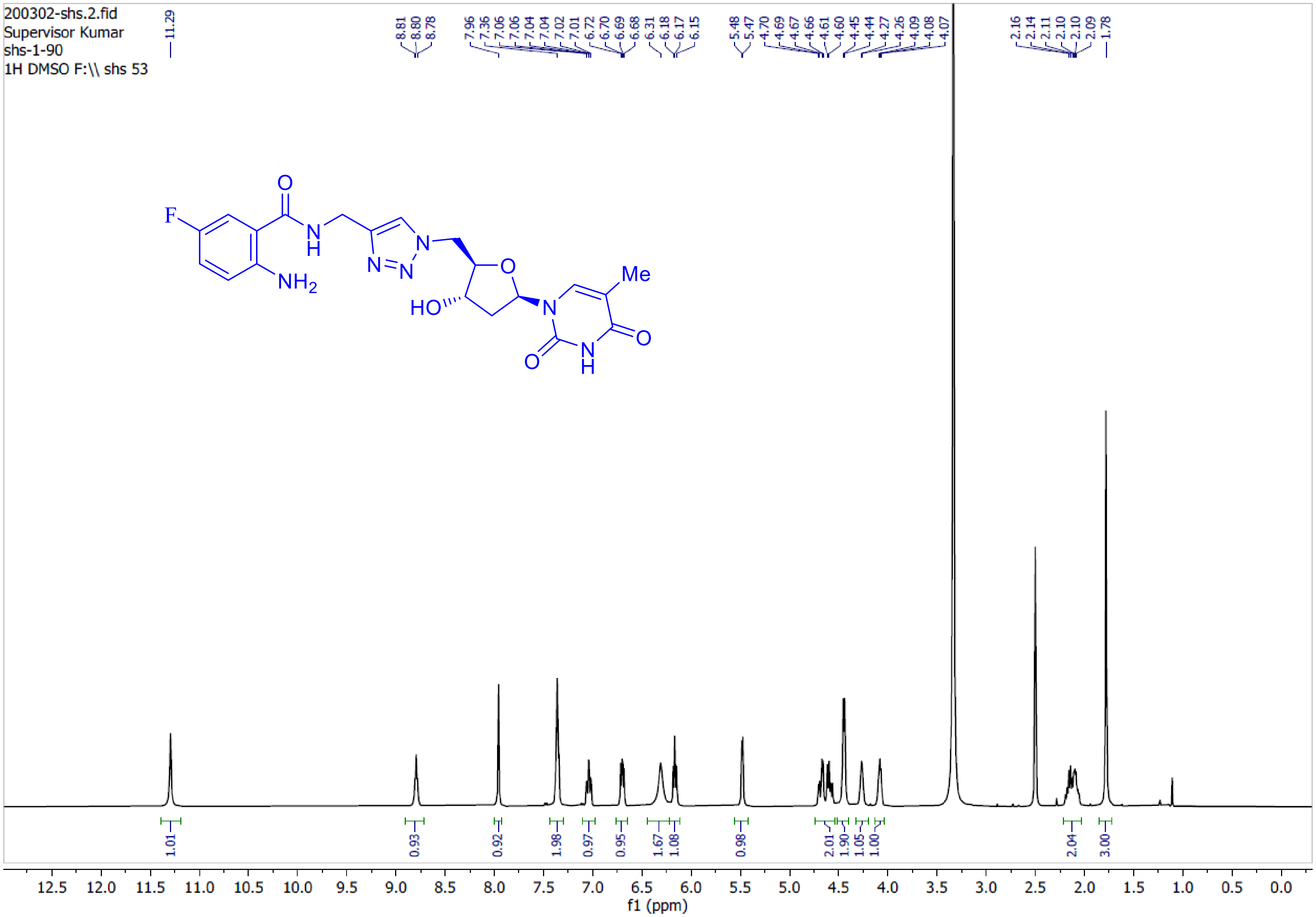

^{13}C NMR spectrum of compound 7c

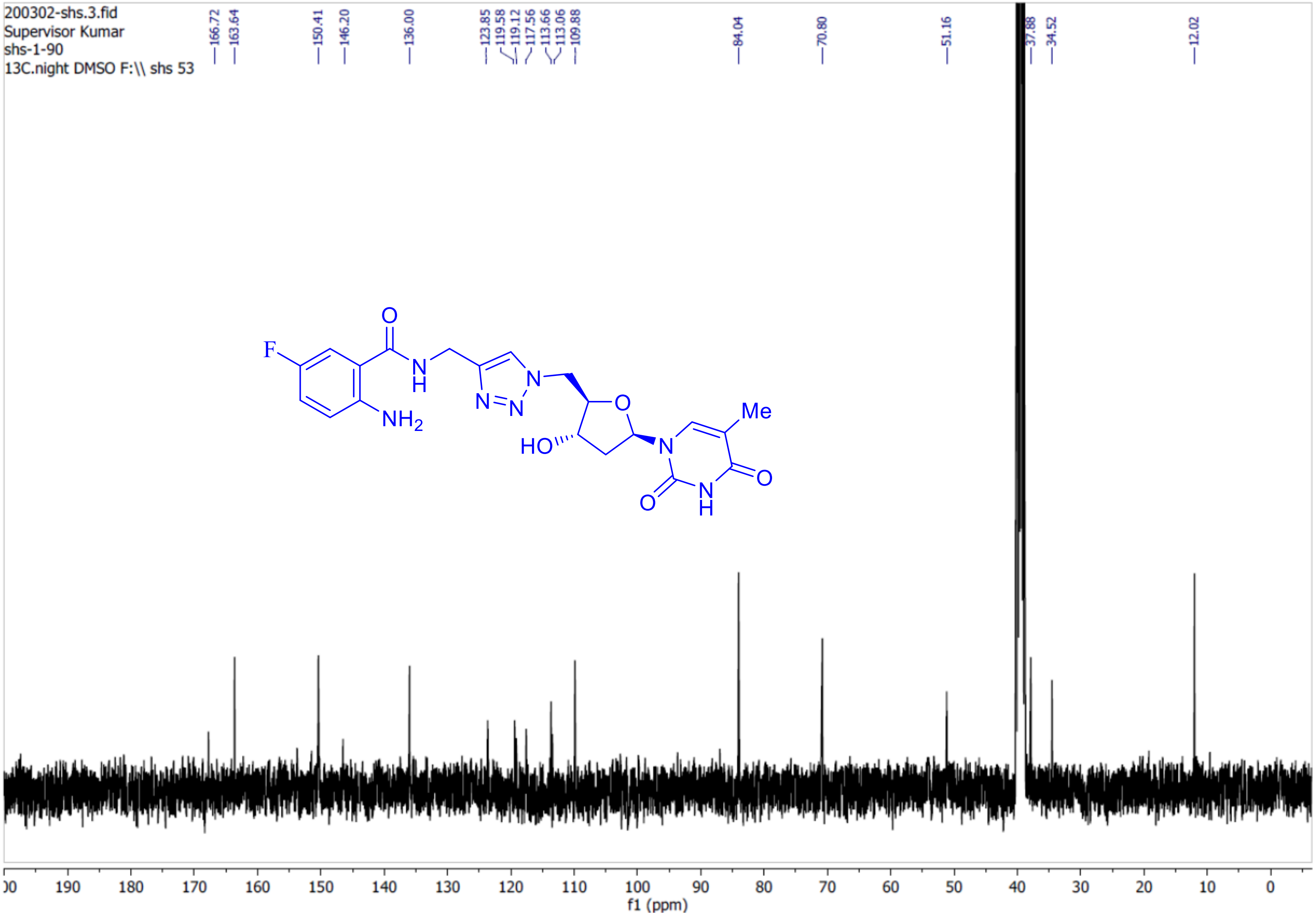

^{1}H NMR spectrum of compound 7d

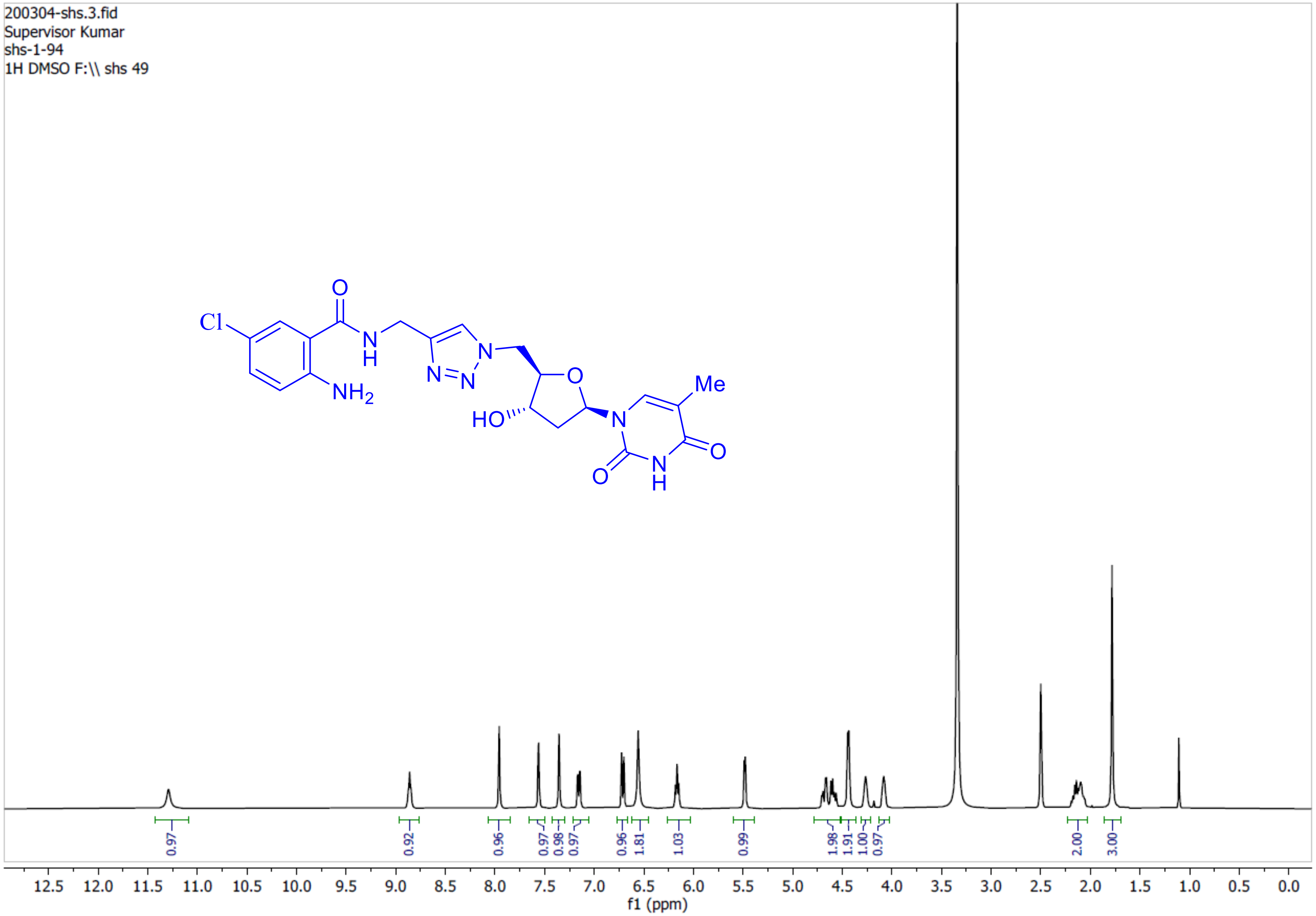

^{13}C NMR spectrum of compound 7d

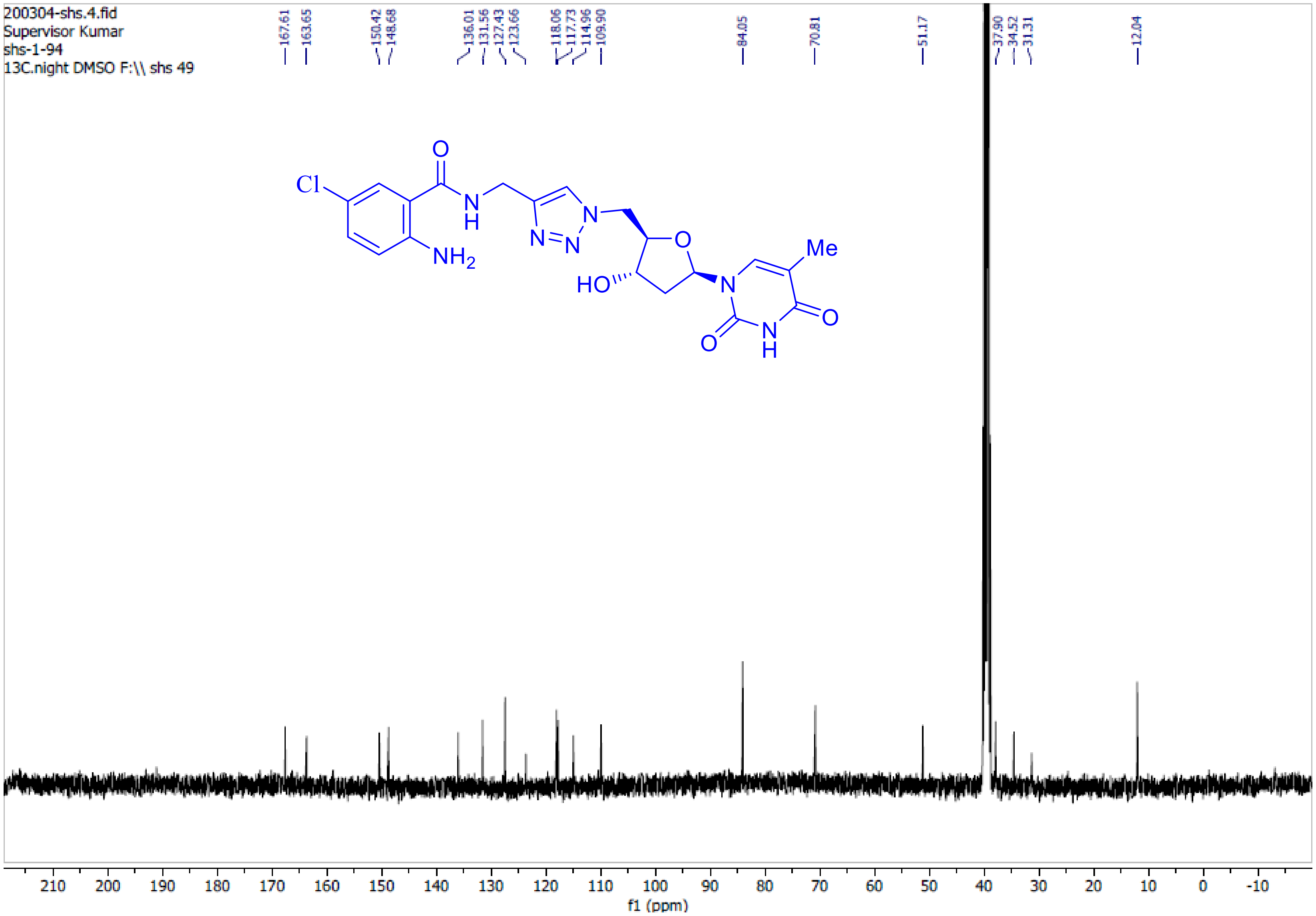

^{1}H NMR spectrum of compound 7e

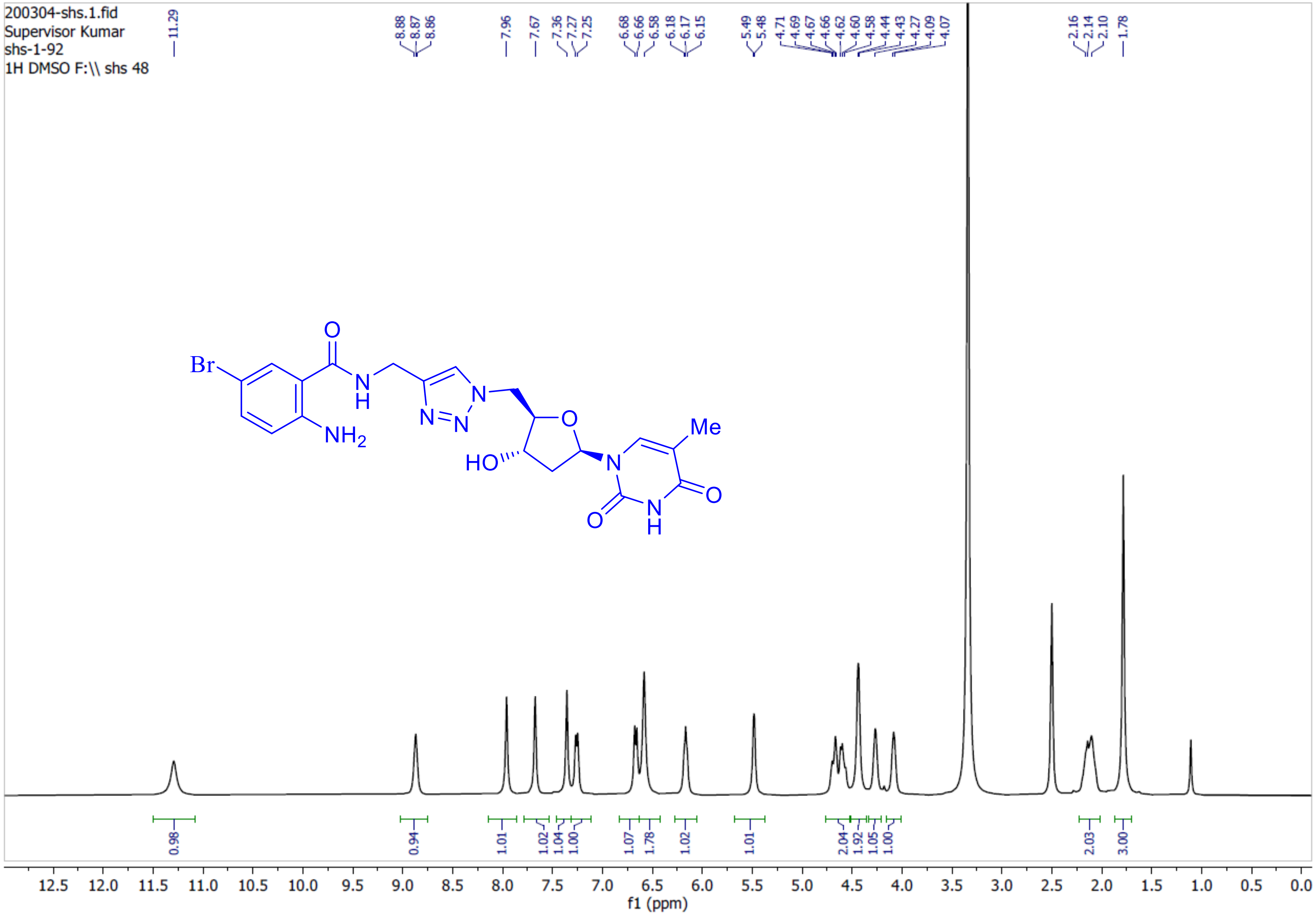

^{13}C NMR spectrum of compound 7e

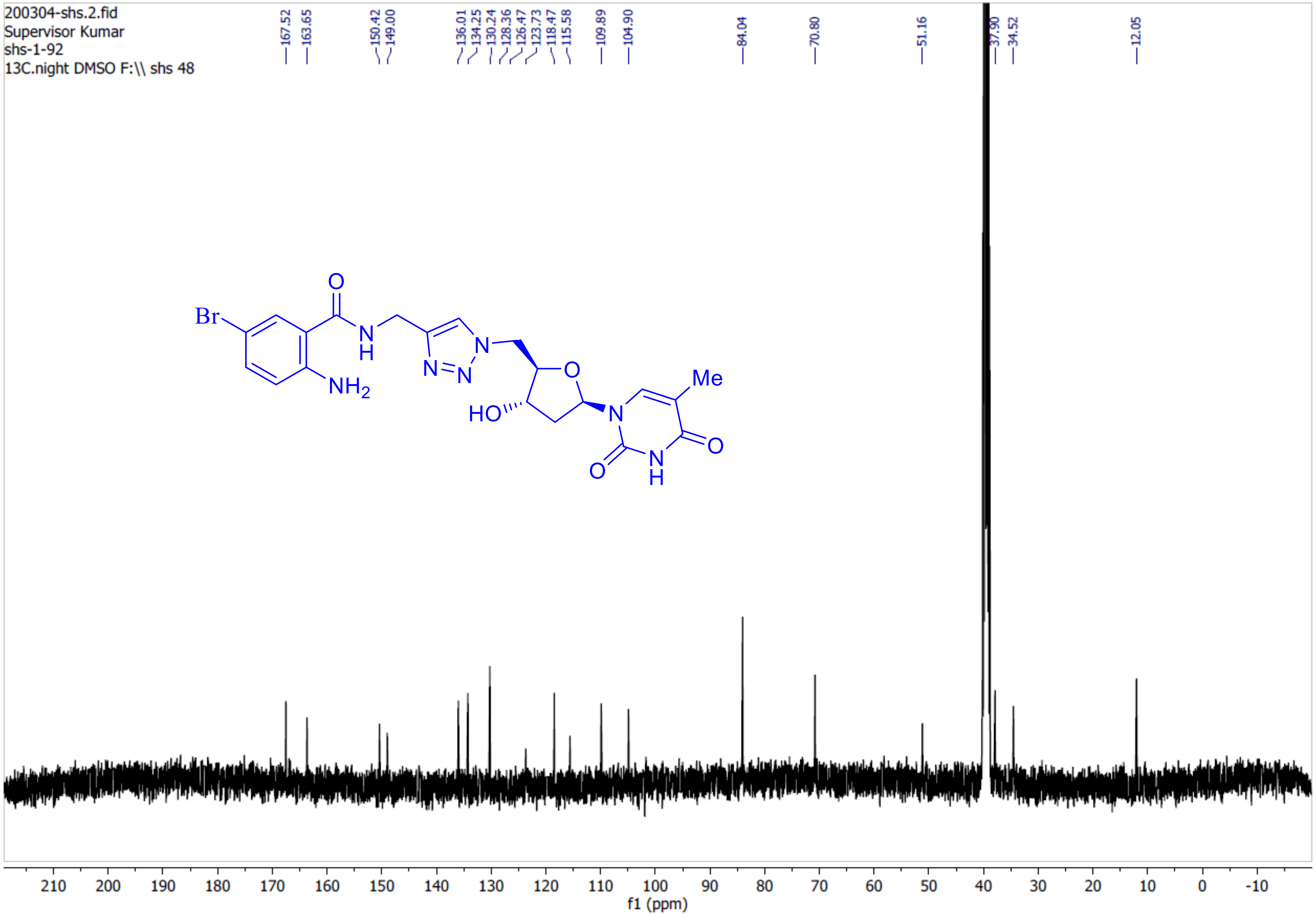

^{1}H NMR spectrum of compound 9a

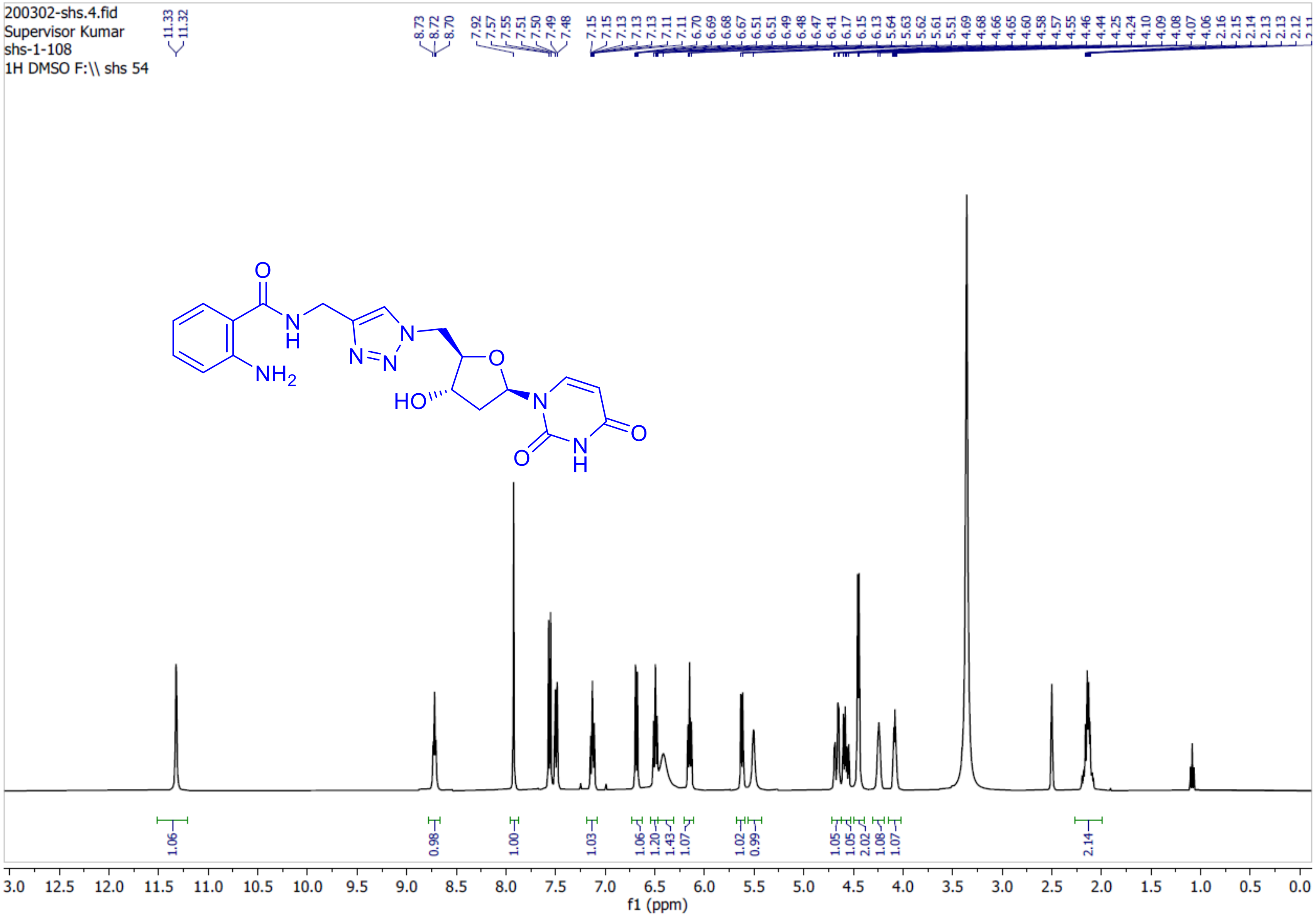

^{13}C NMR spectrum of compound 9a

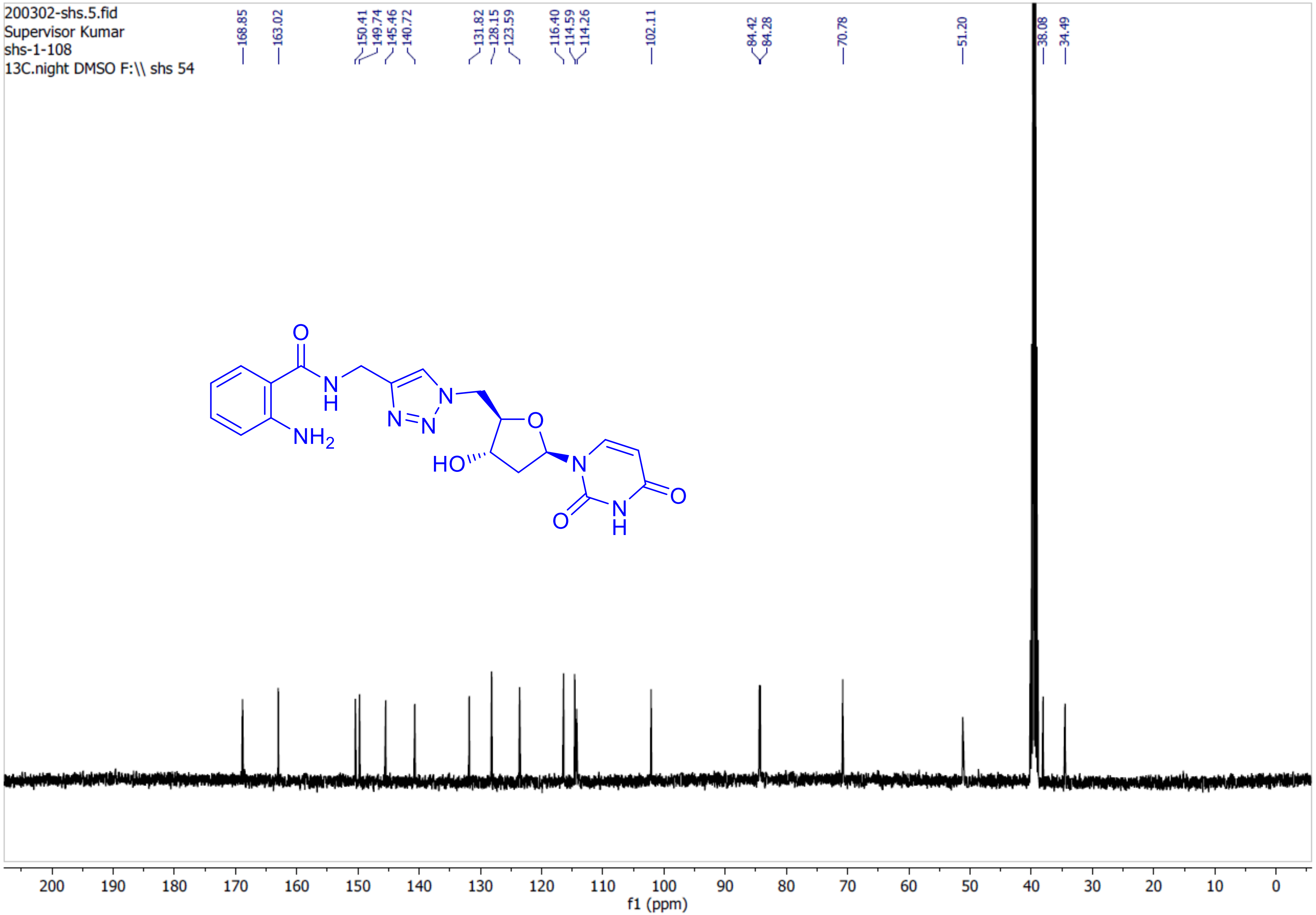

^{1}H NMR spectrum of compound 9b

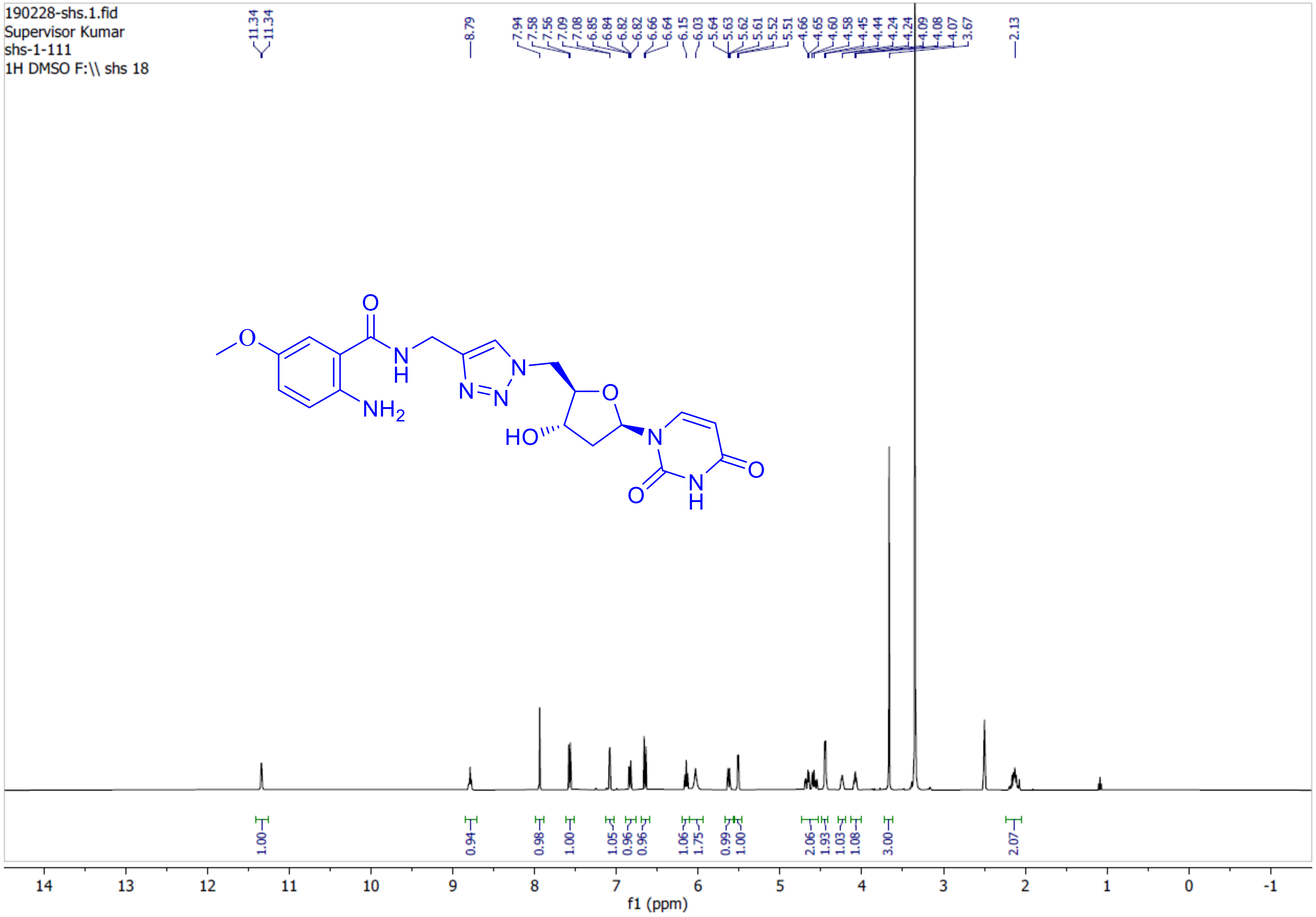

^{13}C NMR spectrum of compound 9b

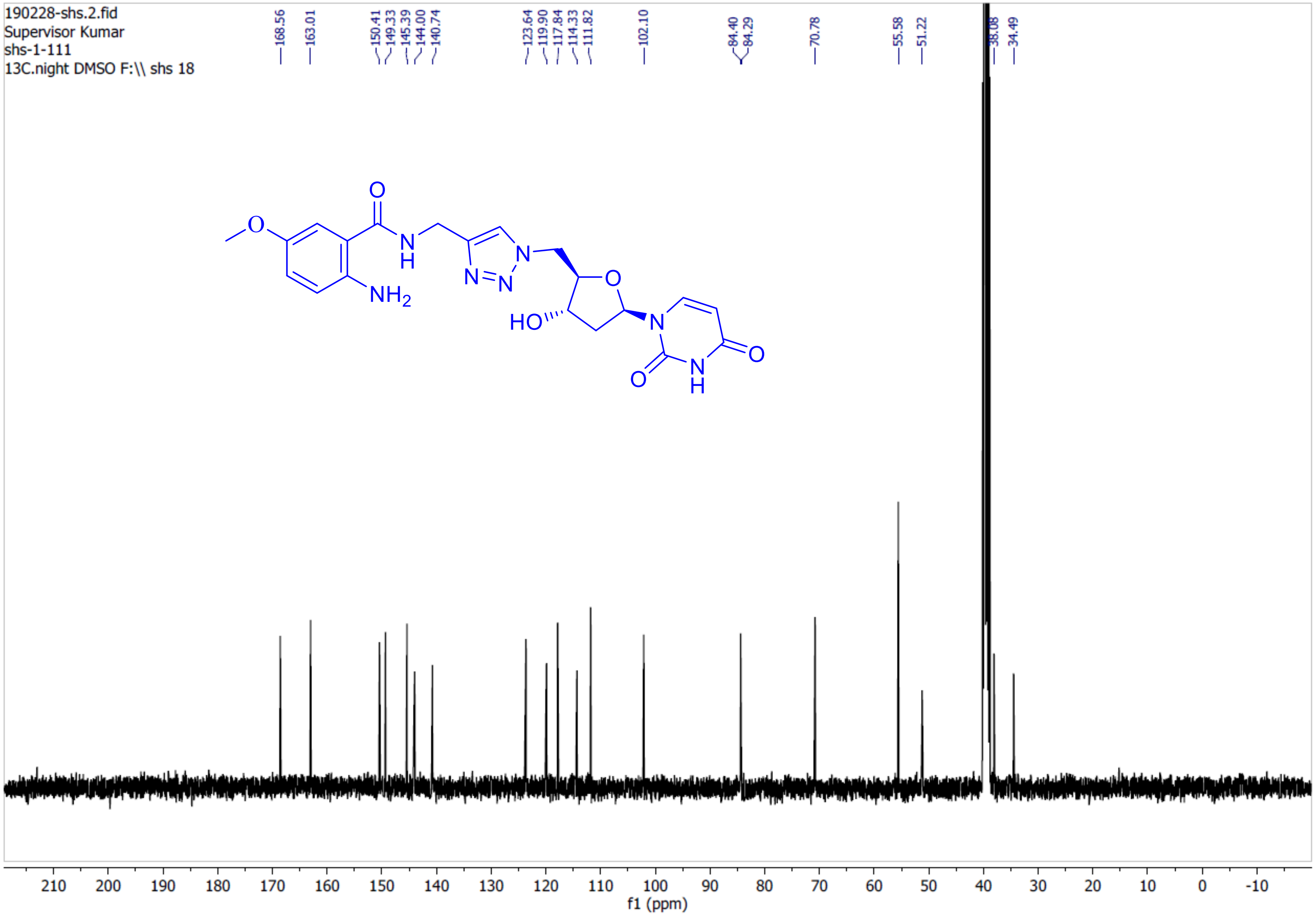

^{1}H NMR spectrum of compound 9c

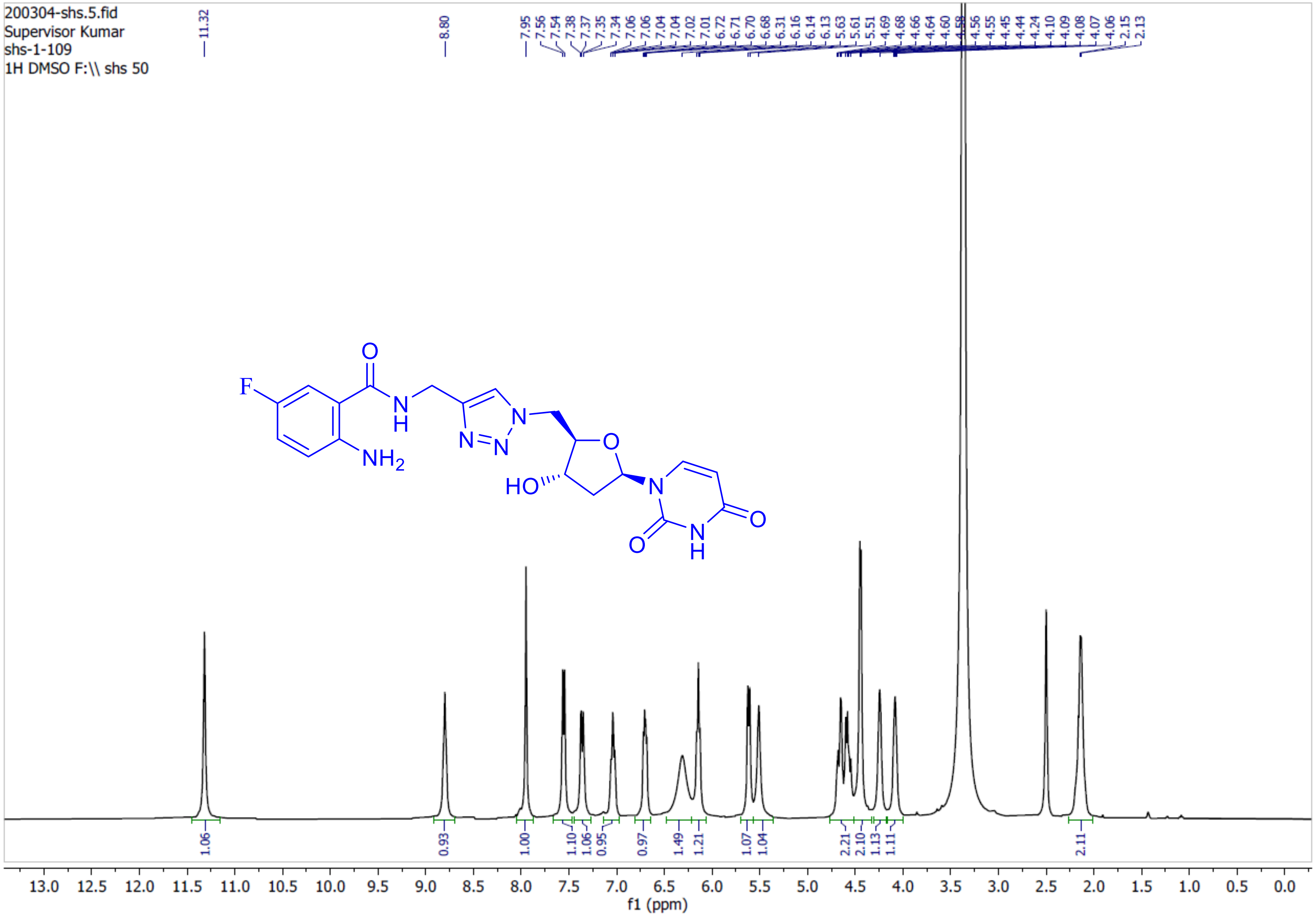

^{13}C NMR spectrum of compound 9c

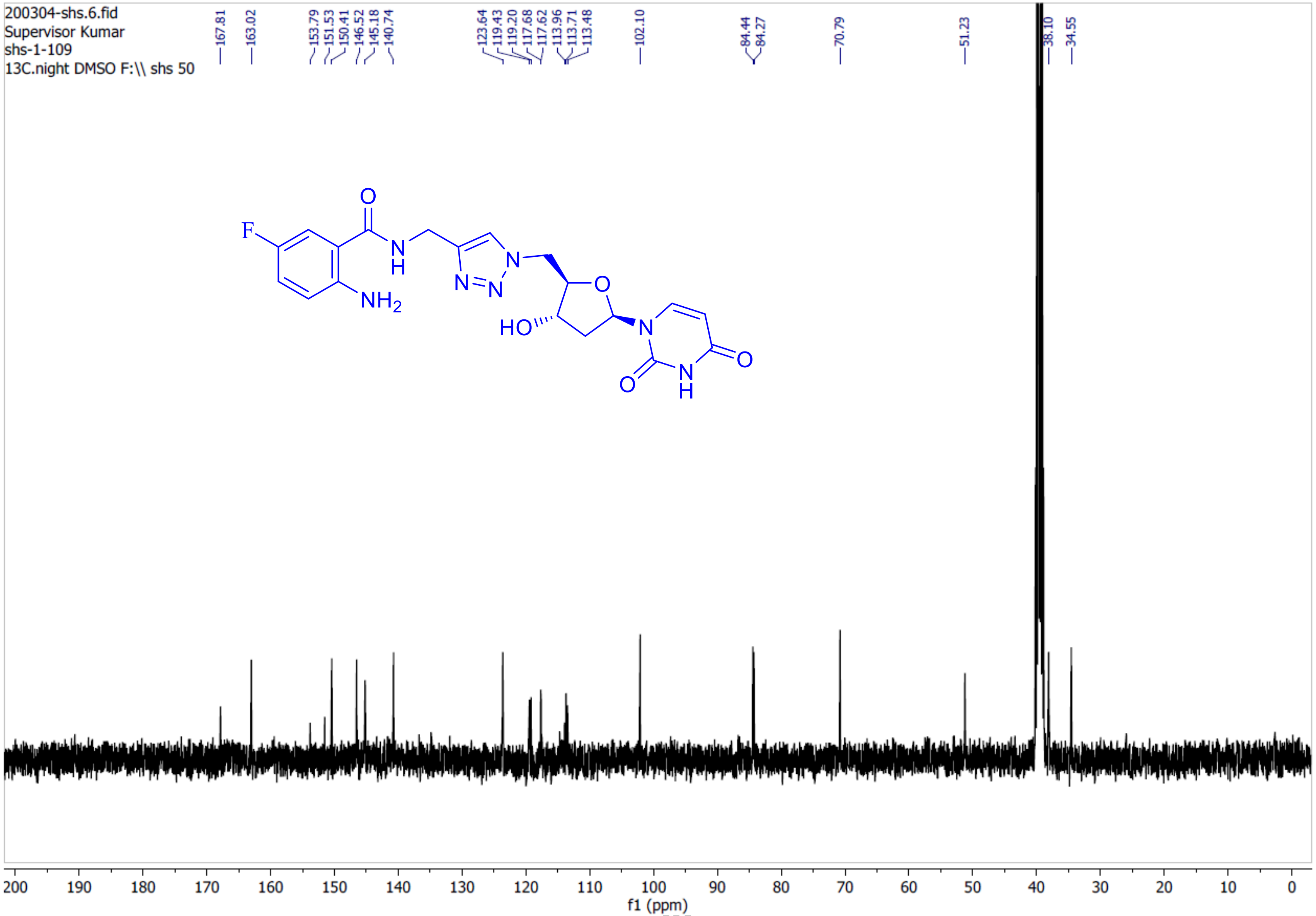

^{1}H NMR spectrum of compound 9e

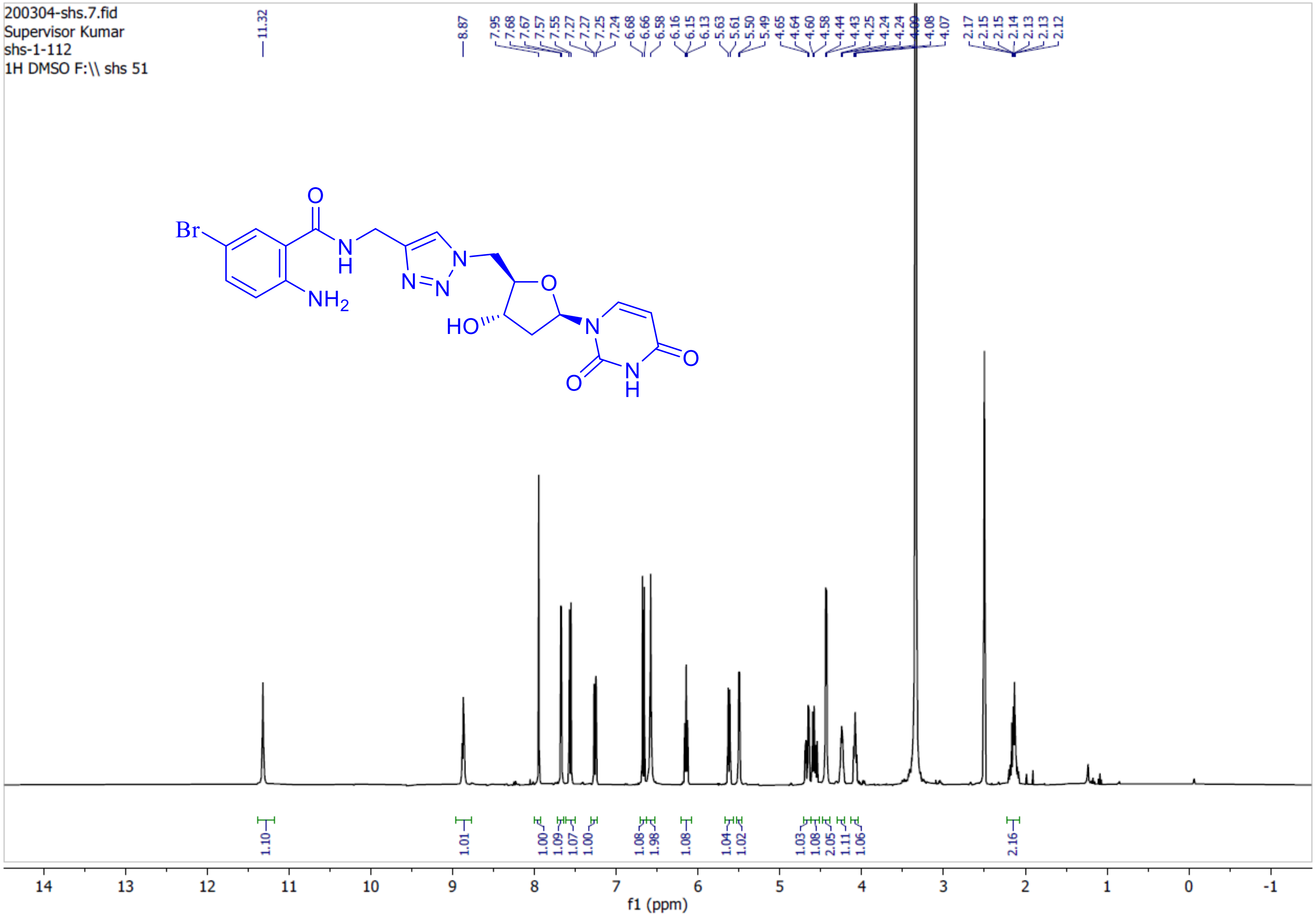

^{13}C NMR spectrum of compound 9e

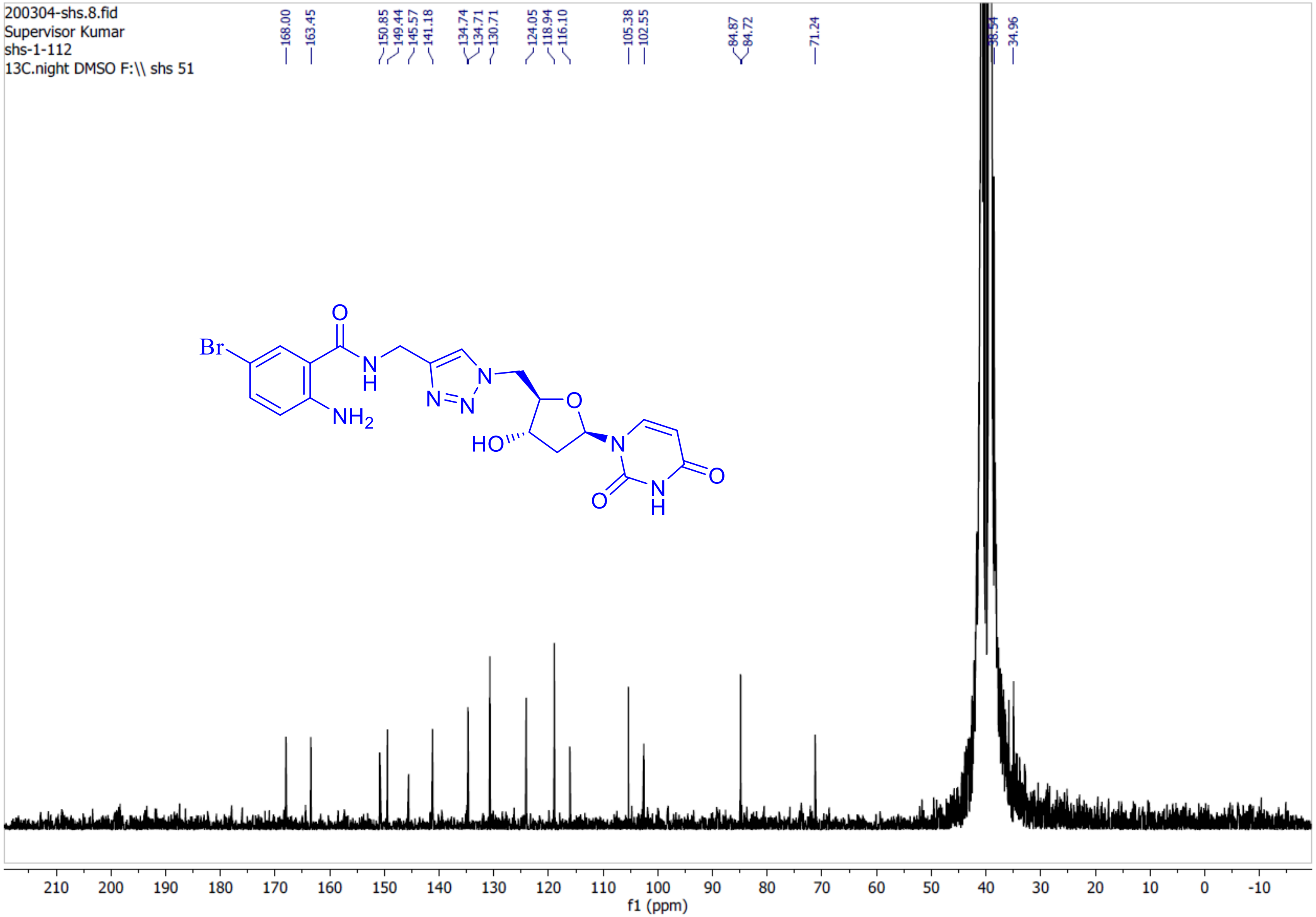

^{1}H NMR spectrum of compound 12a

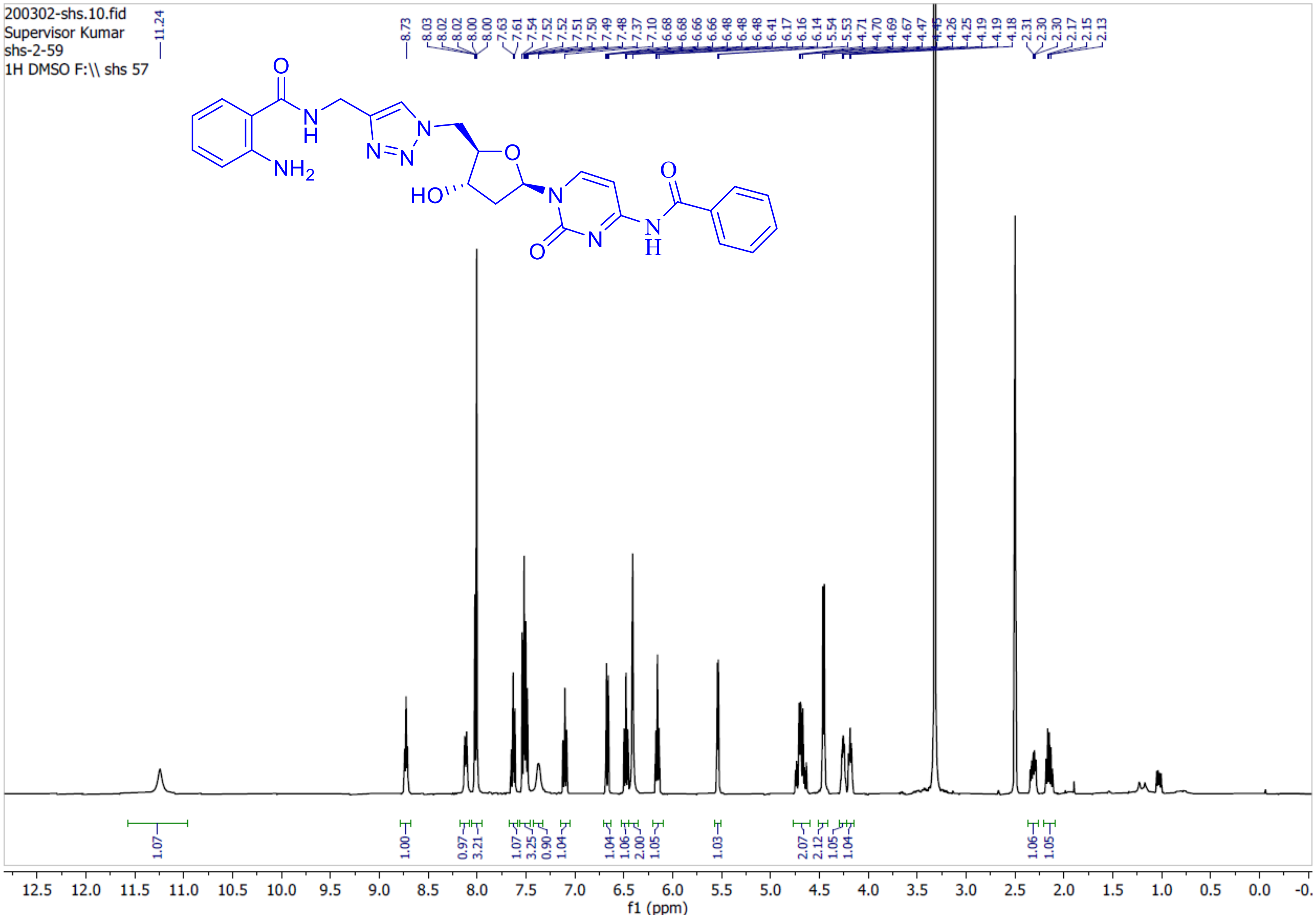

^{13}C NMR spectrum of compound 12a

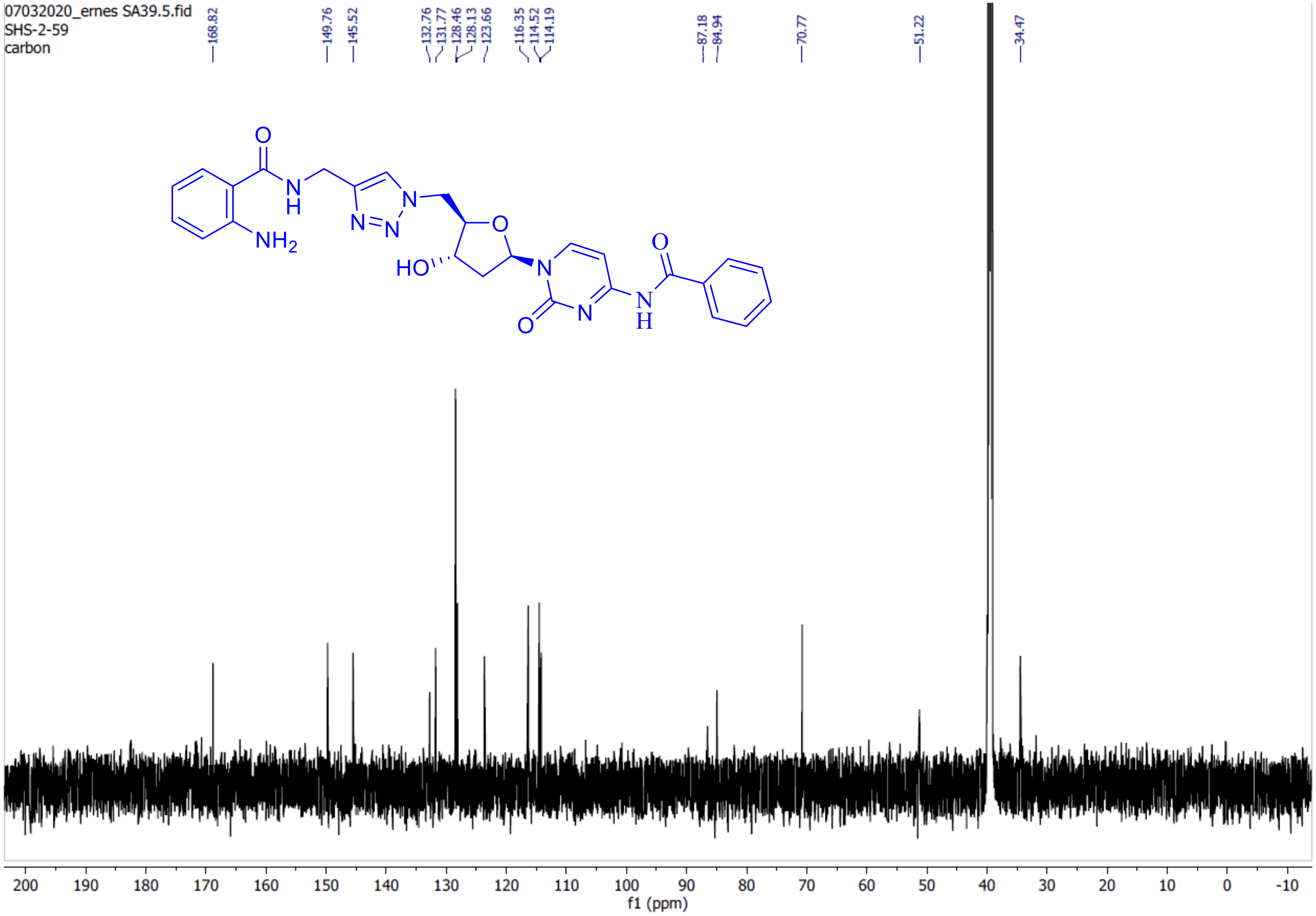

^{1}H NMR spectrum of compound 12b

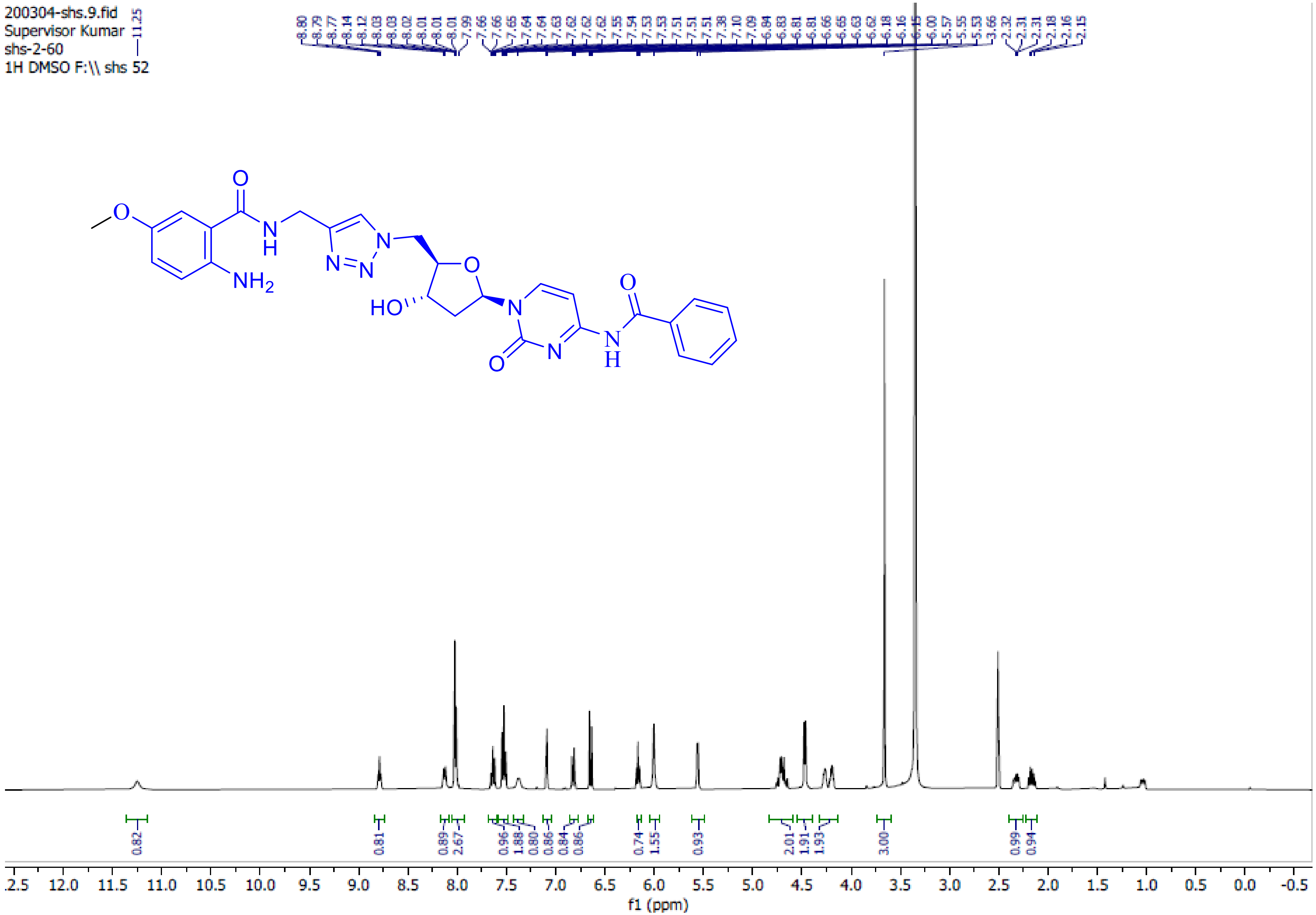

^{13}C NMR spectrum of compound 12b

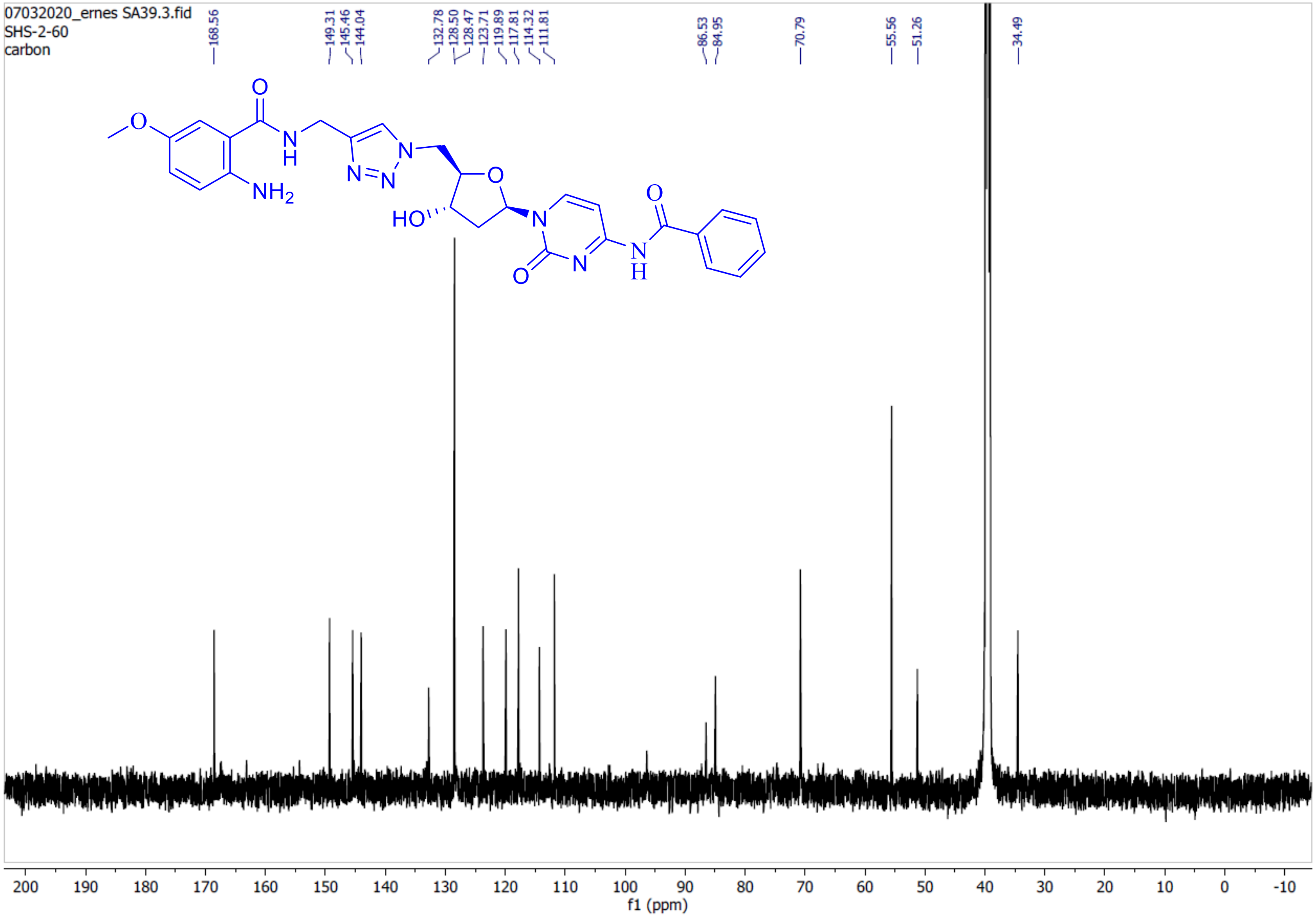

1H NMR spectrum of compound 12c

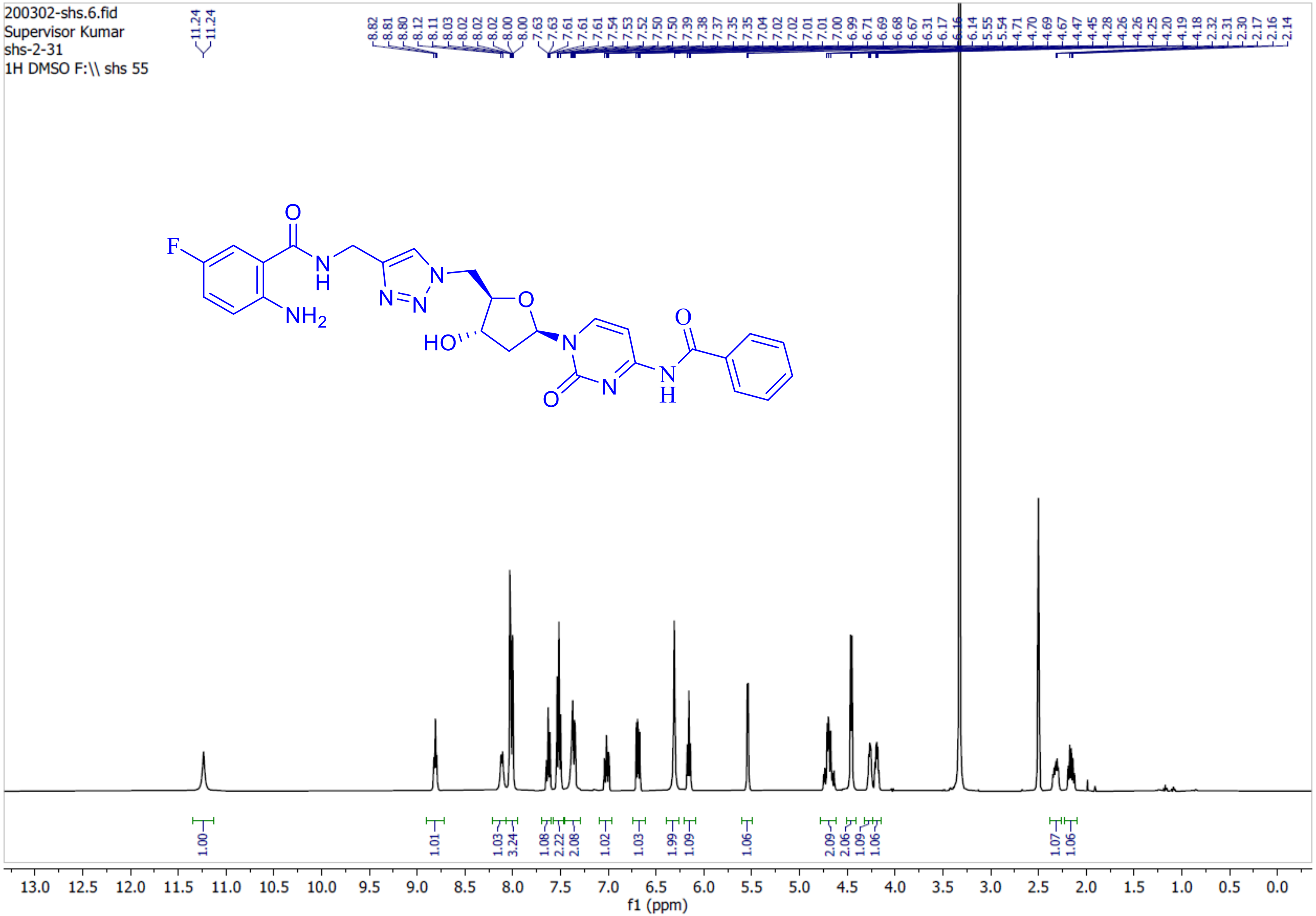

^{13}C NMR spectrum of compound 12c

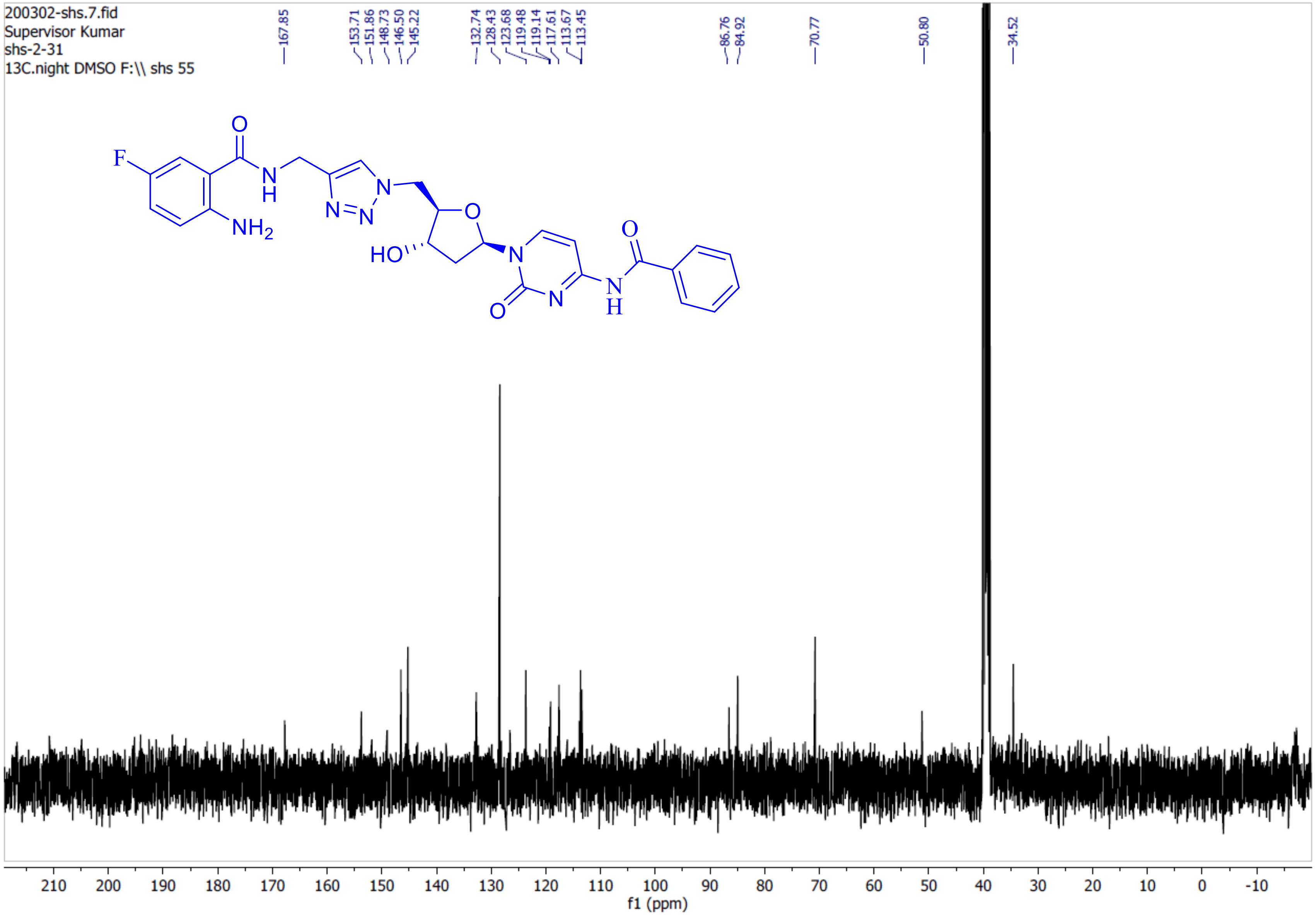

^1H NMR spectrum of compound 12d

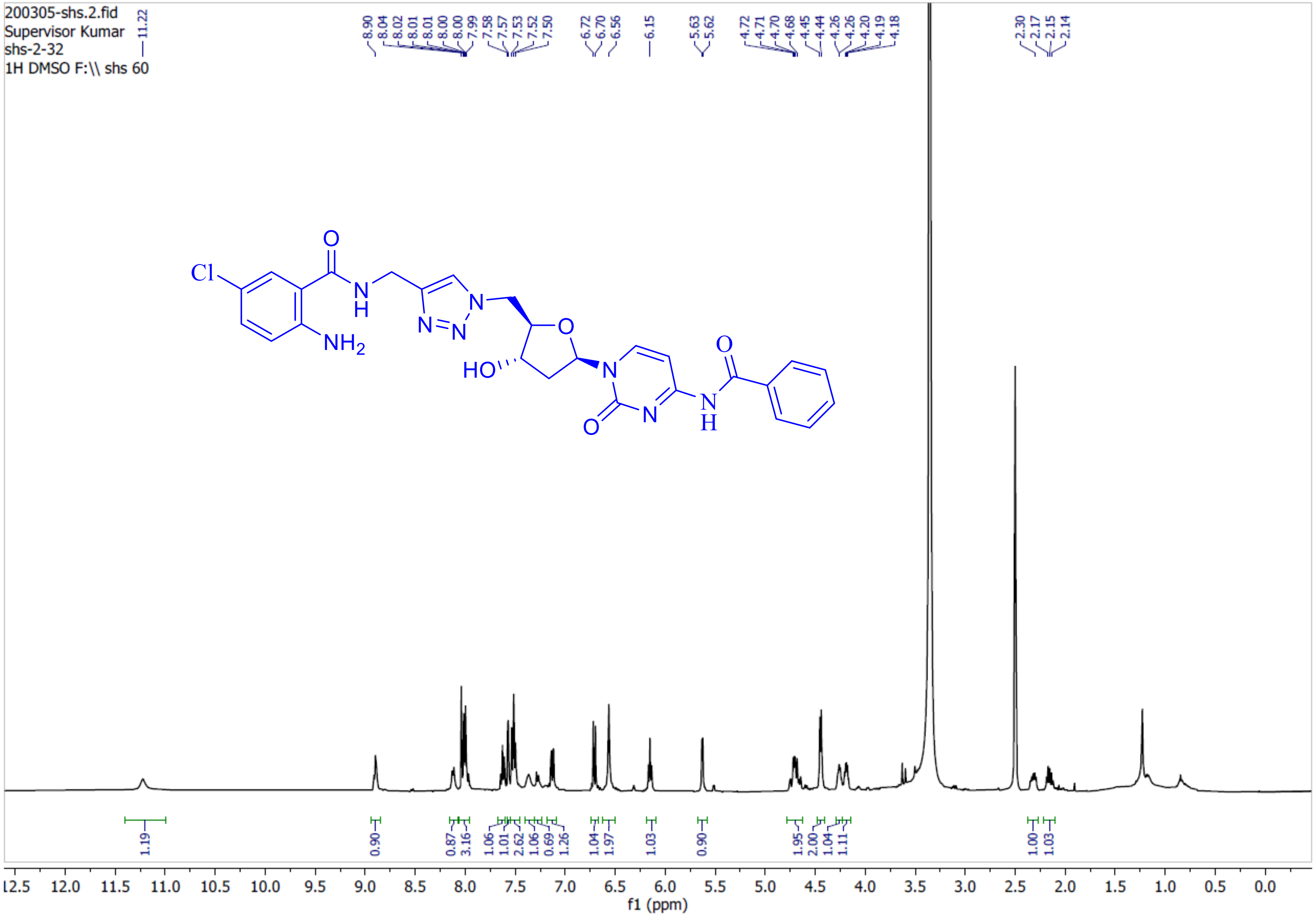

^{13}C NMR spectrum of compound 12d

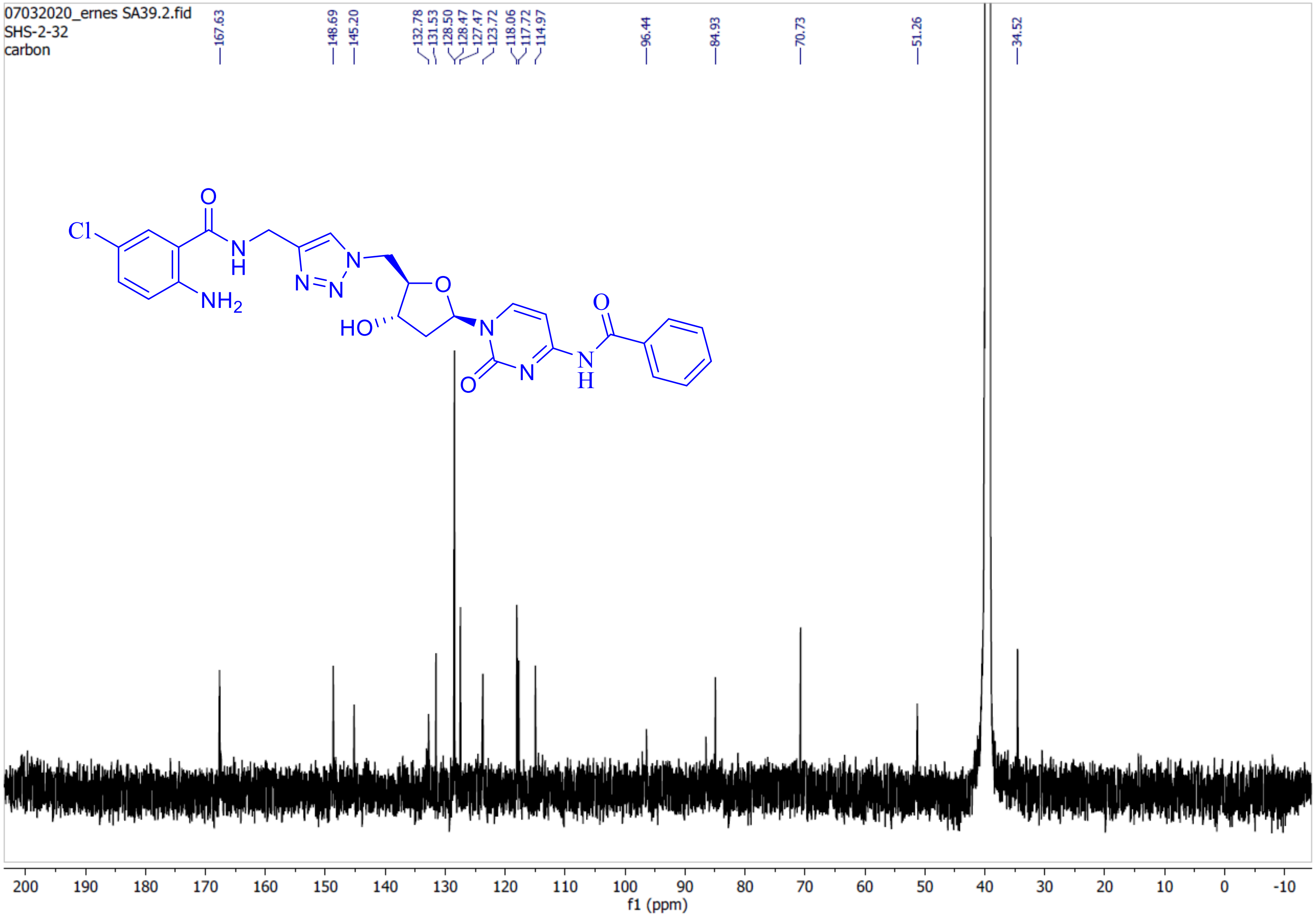

^{1}H NMR spectrum of compound 12e

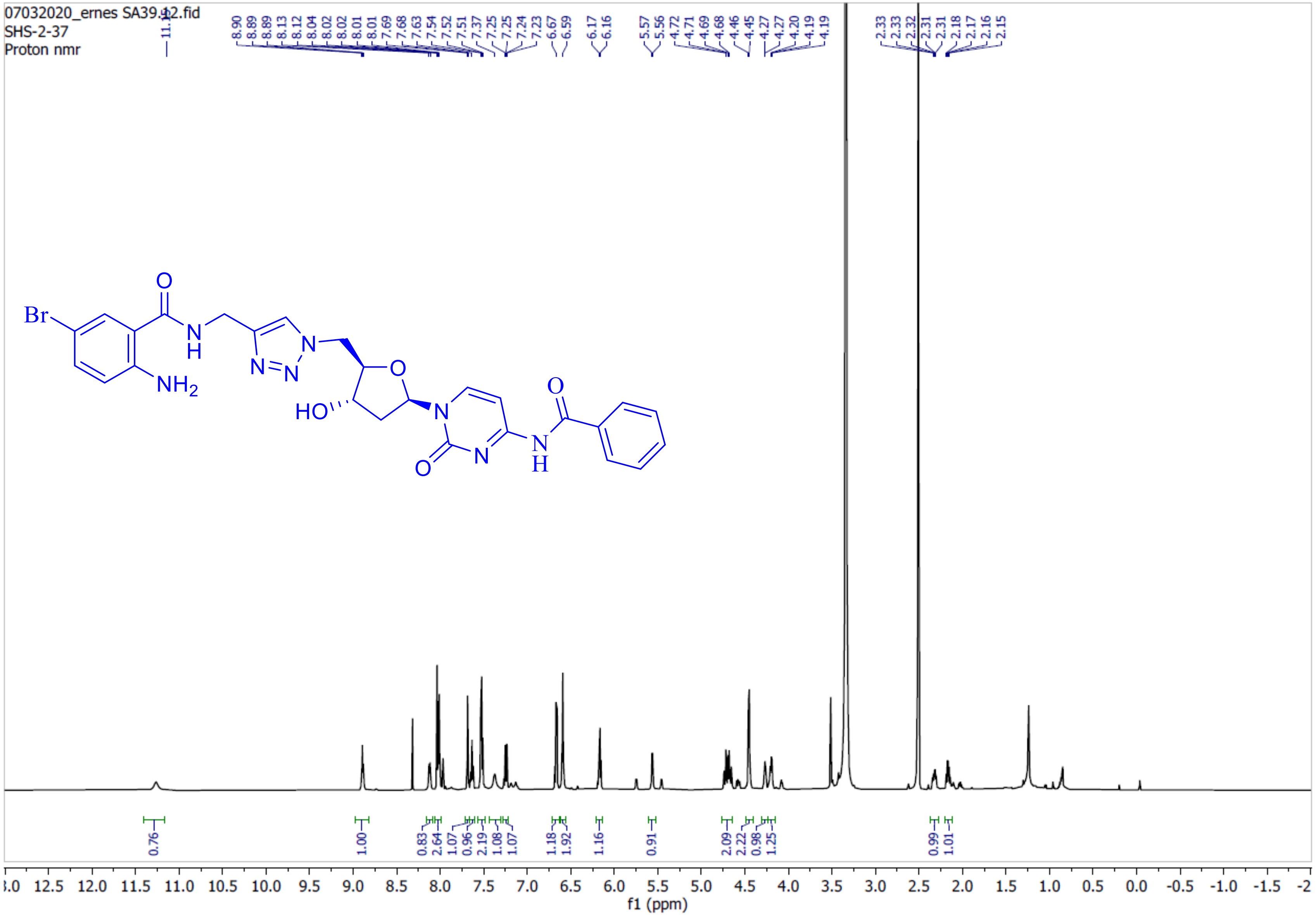

^{13}C NMR spectrum of compound 12e

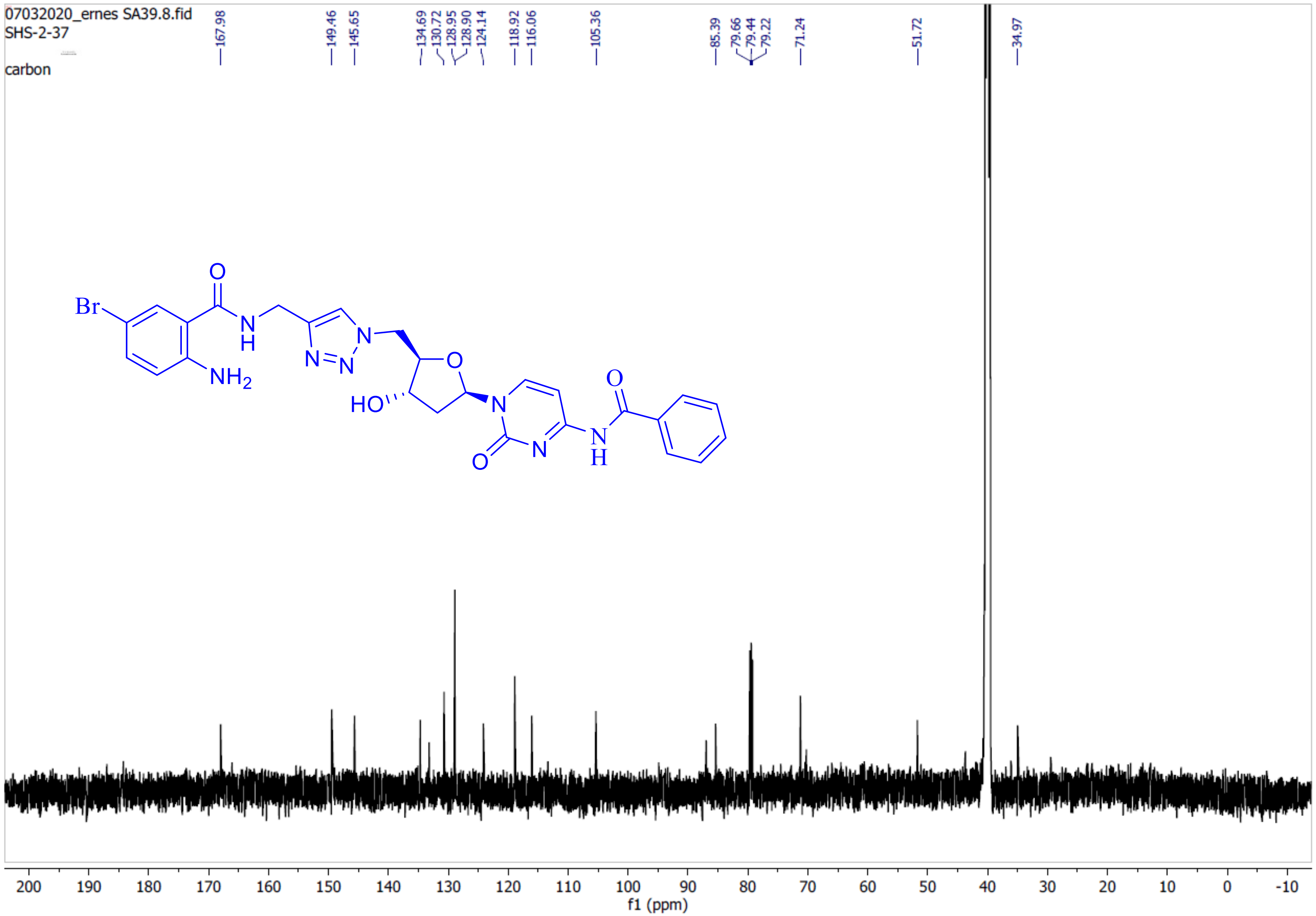

^{1}H NMR spectrum of compound 13a

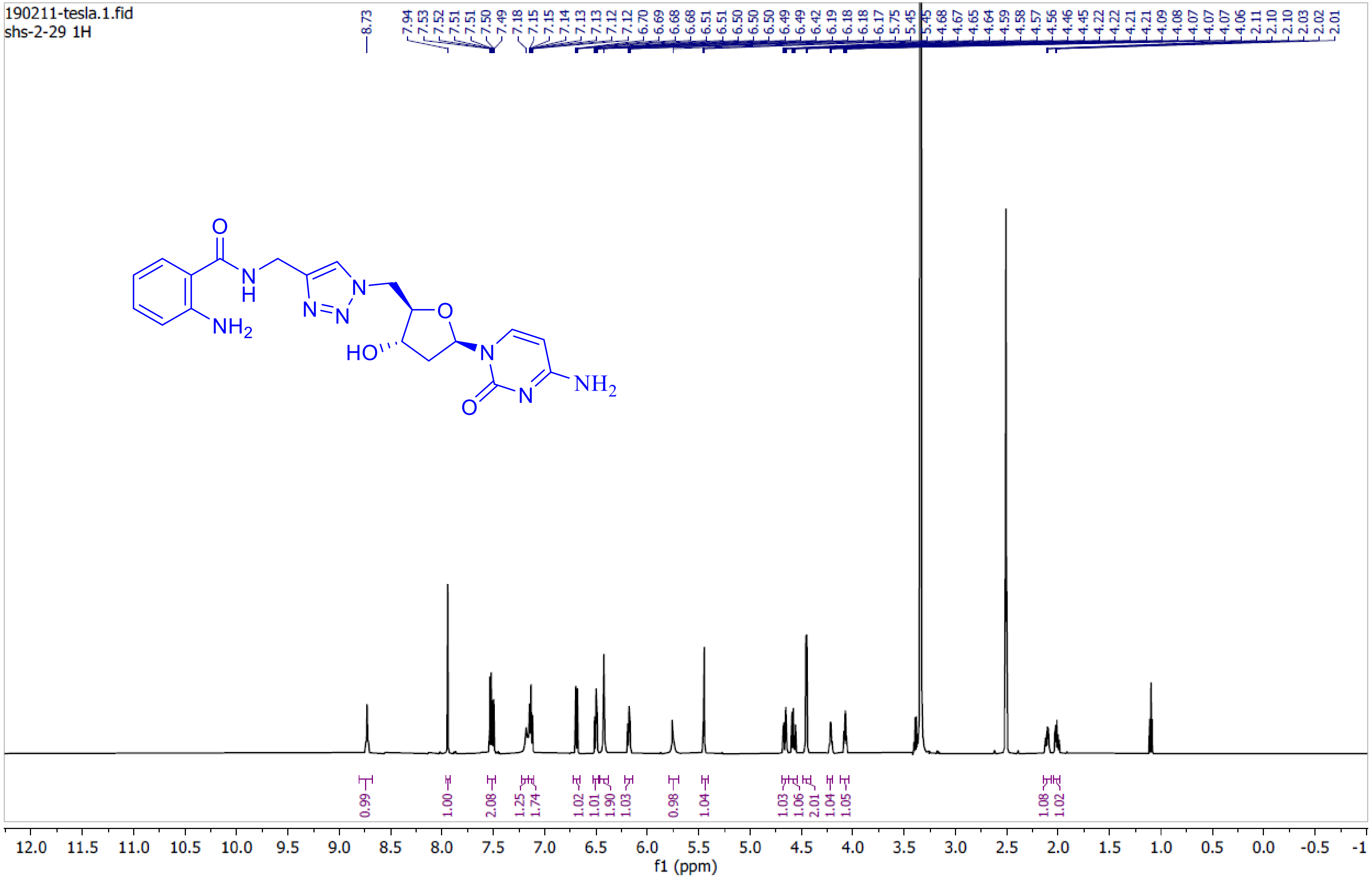

^{13}C NMR spectrum of compound 13a

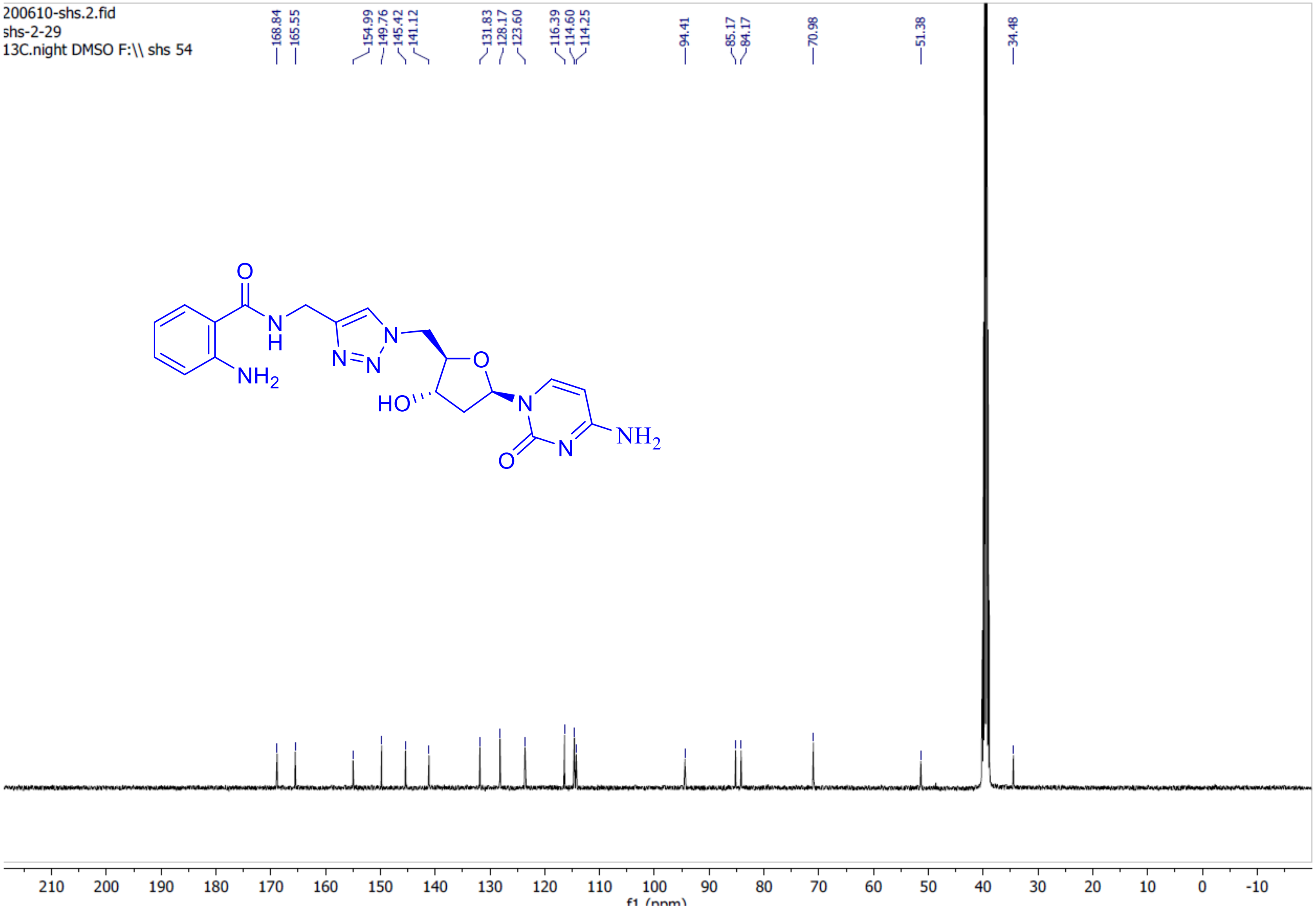

^{1}H NMR spectrum of compound 13b

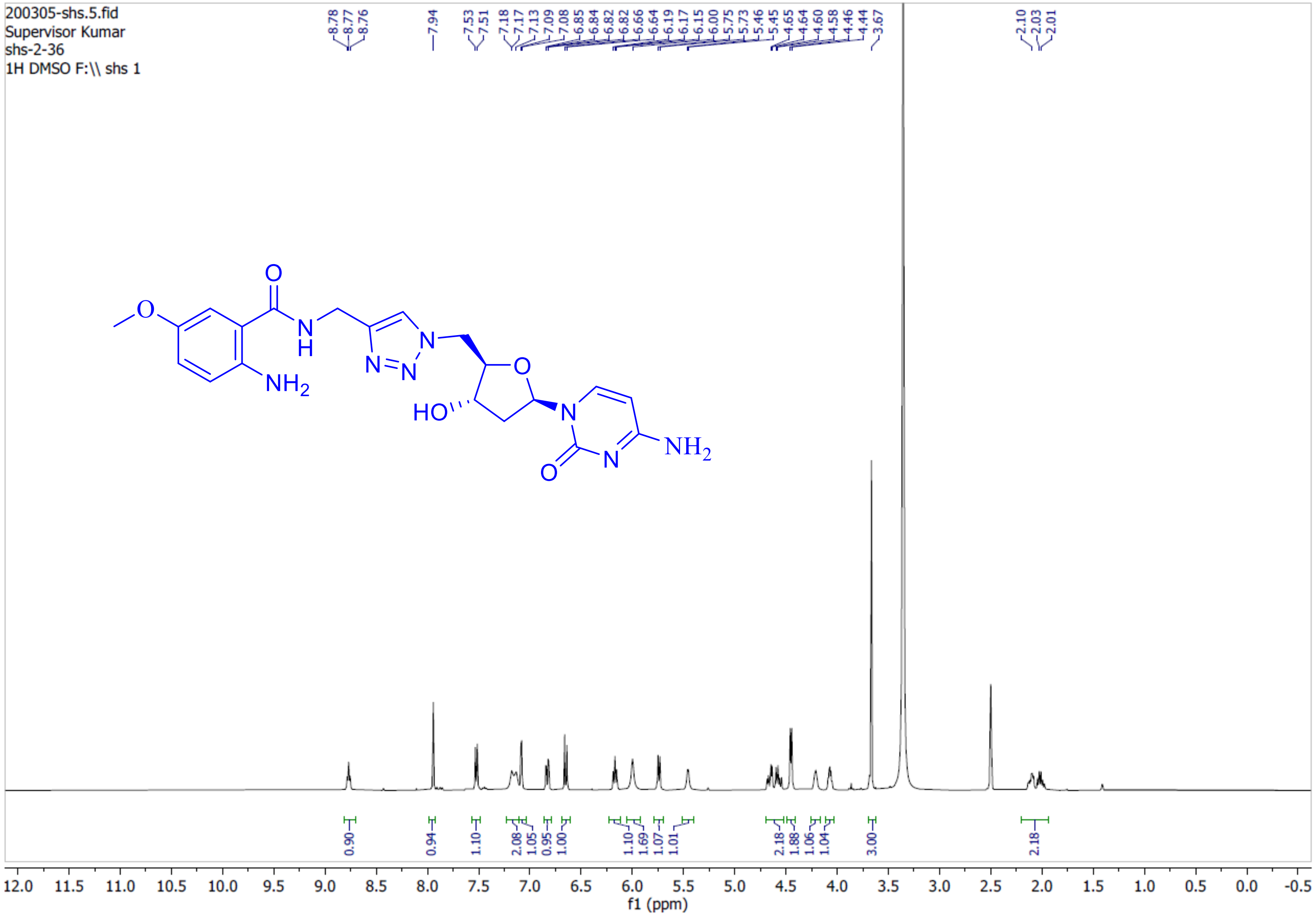

^{13}C NMR spectrum of compound 13b

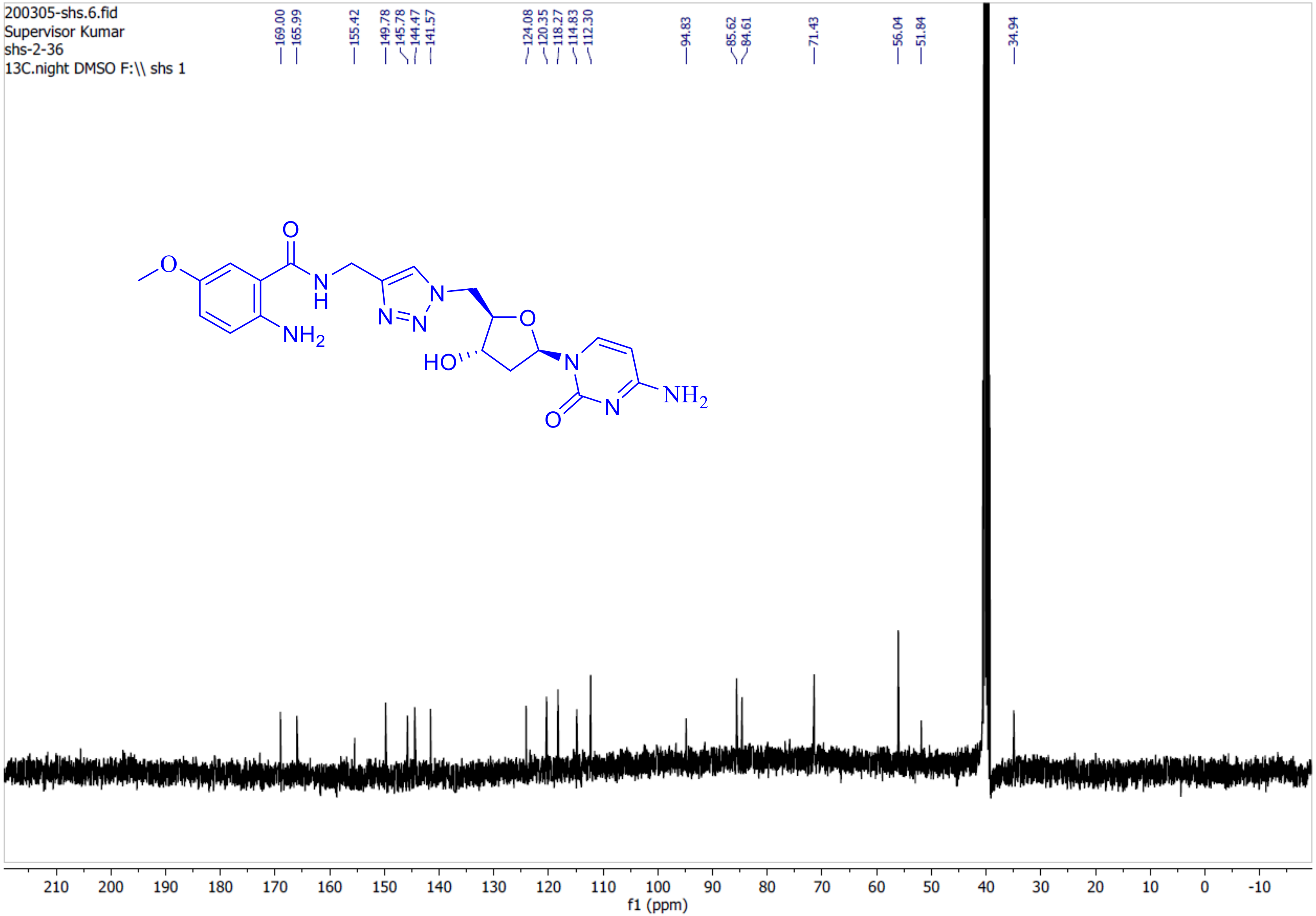

^{1}H NMR spectrum of compound 13c

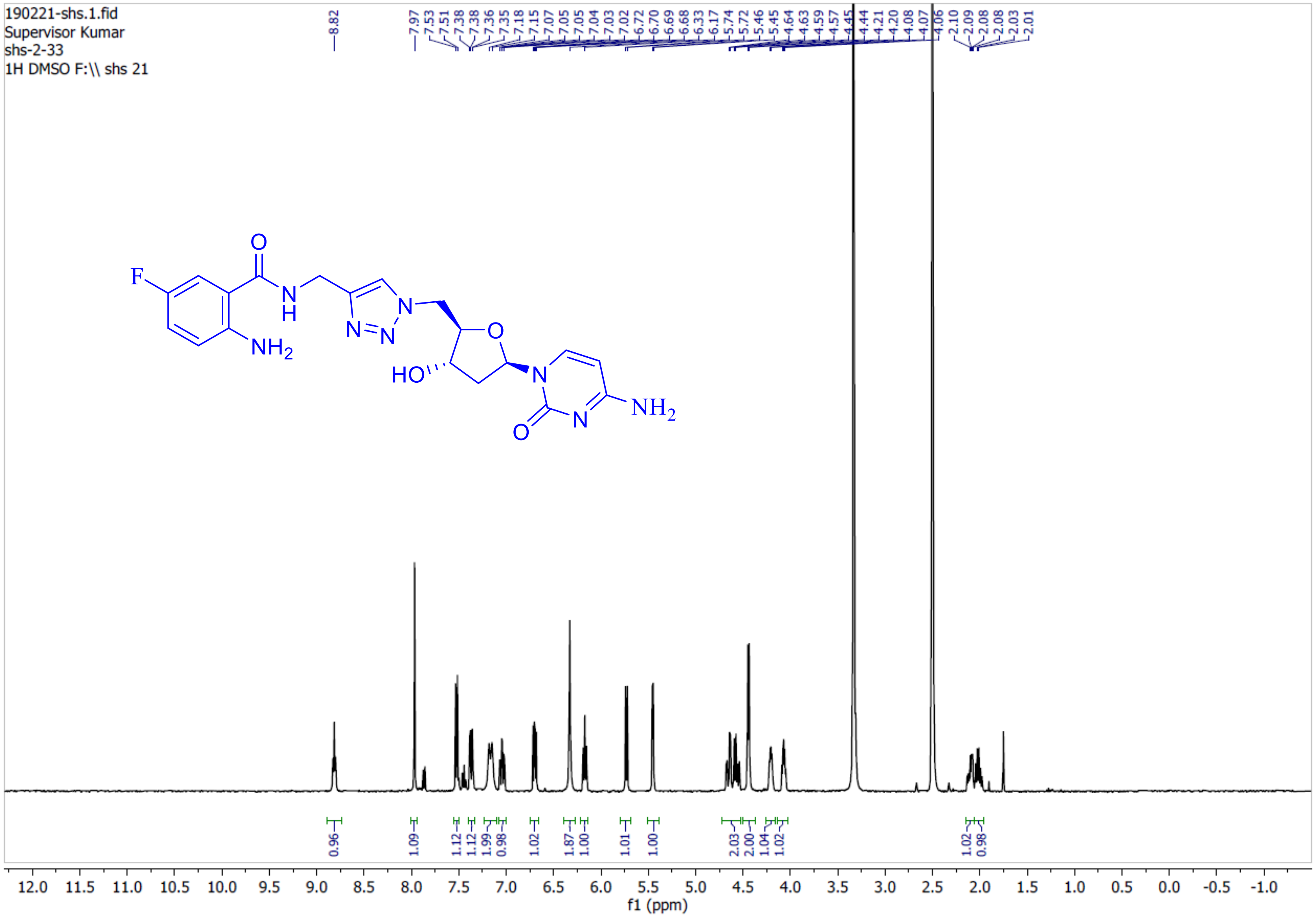

^{13}C NMR spectrum of compound 13c

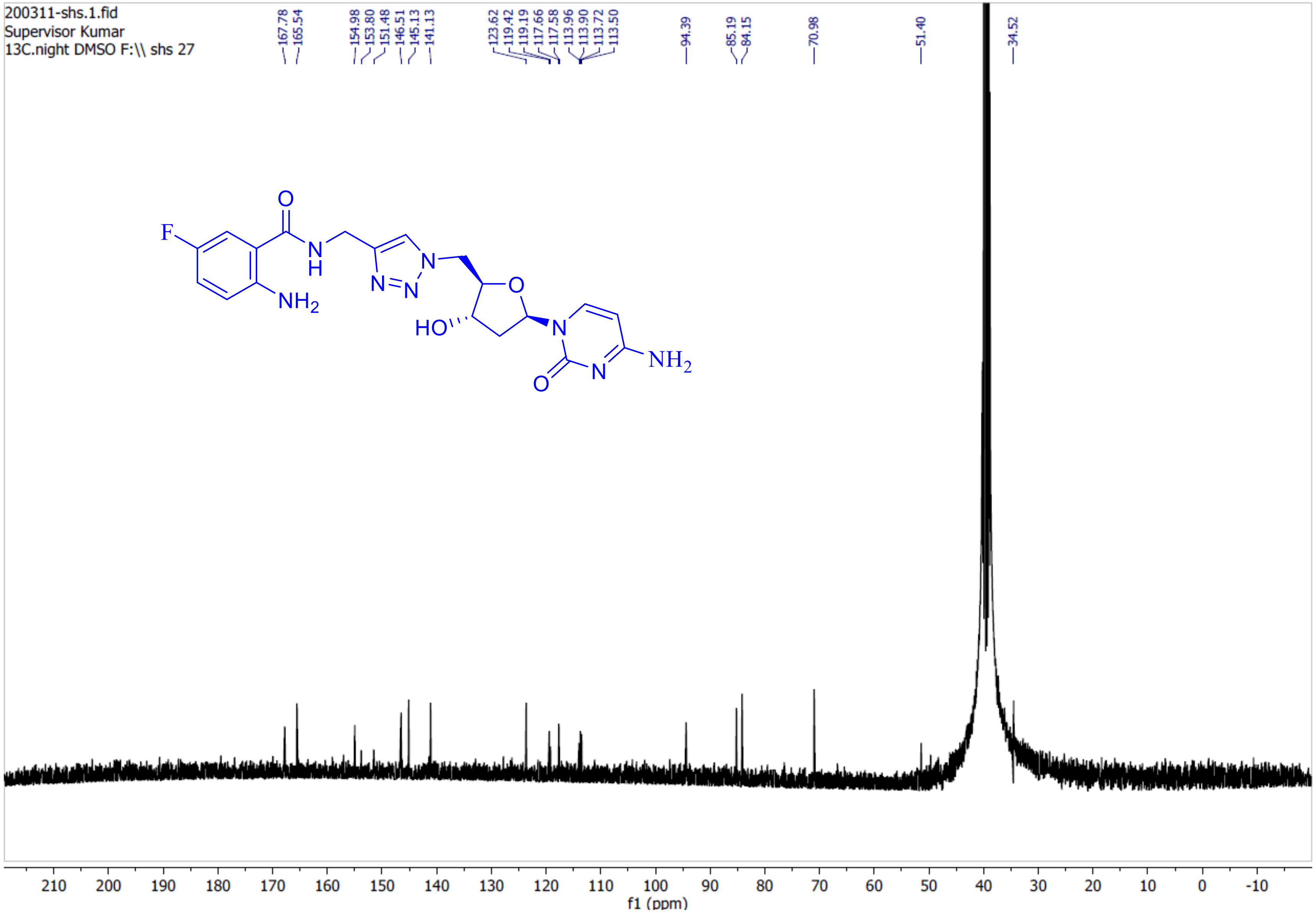

^{1}H NMR spectrum of compound 13d

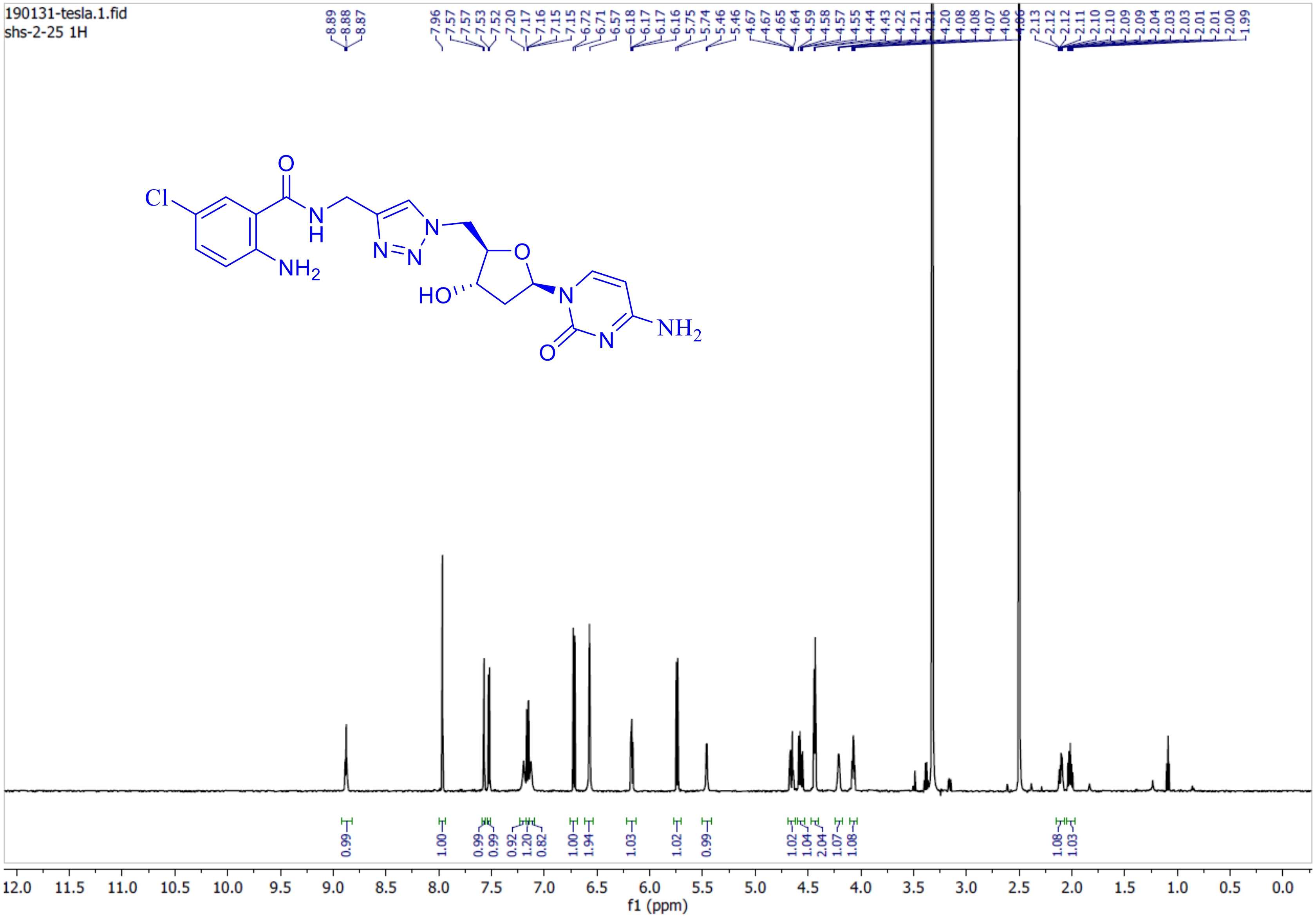

^{13}C NMR spectrum of compound 13d

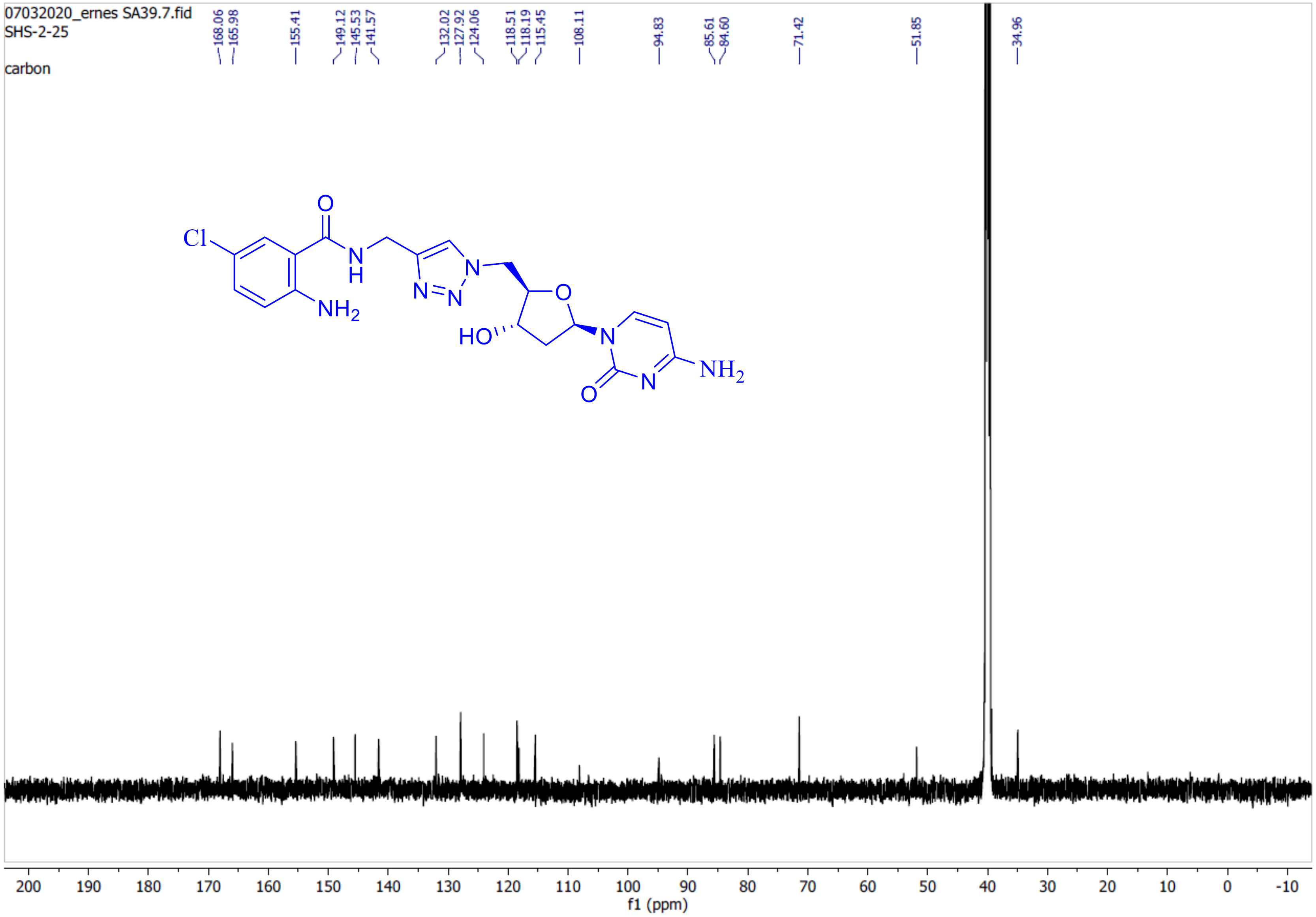

^{1}H NMR spectrum of compound 13e

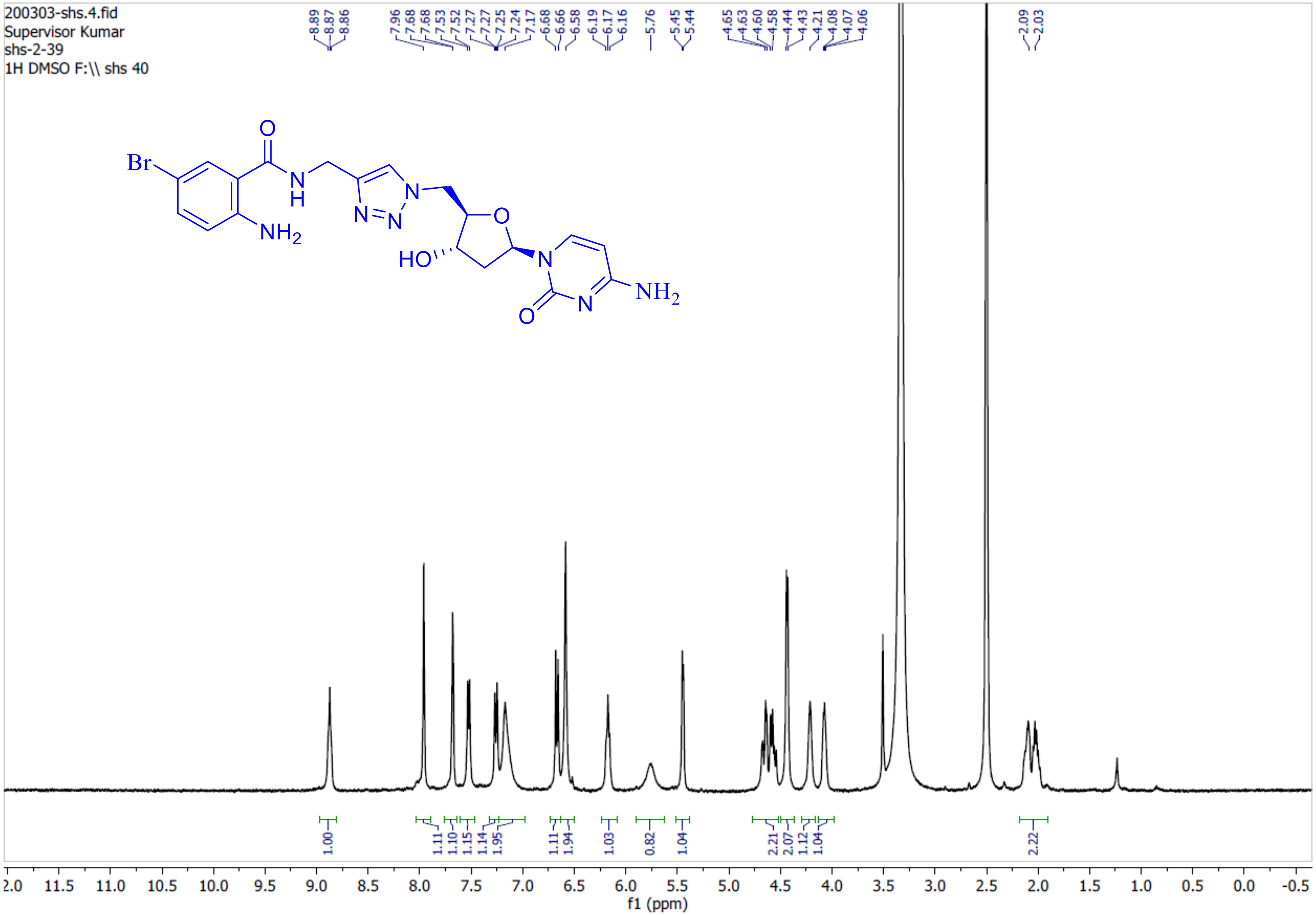

^{13}C NMR spectrum of compound 13e

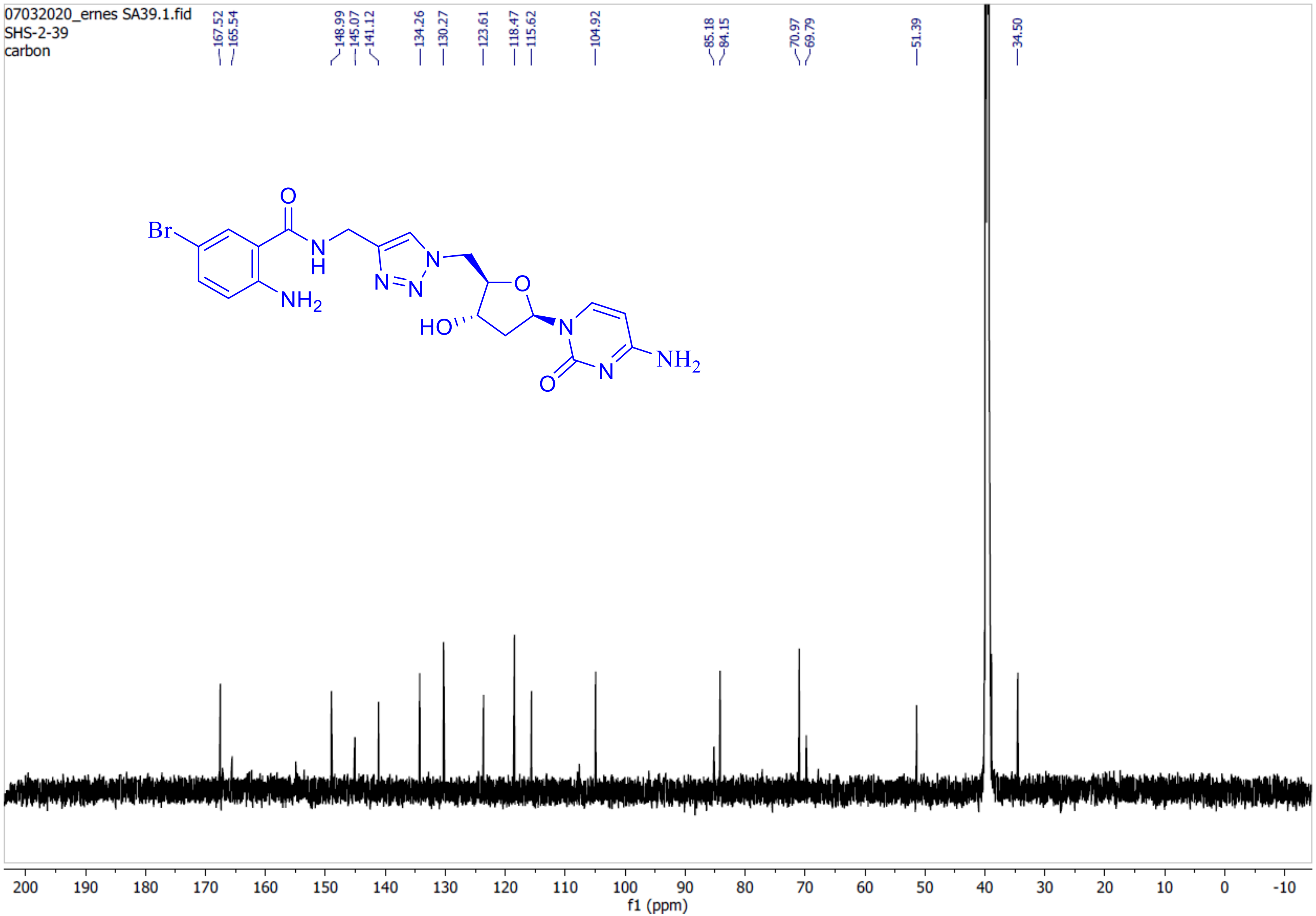

^{1}H- ^{1}H cosy NMR spectrum of compound 13a

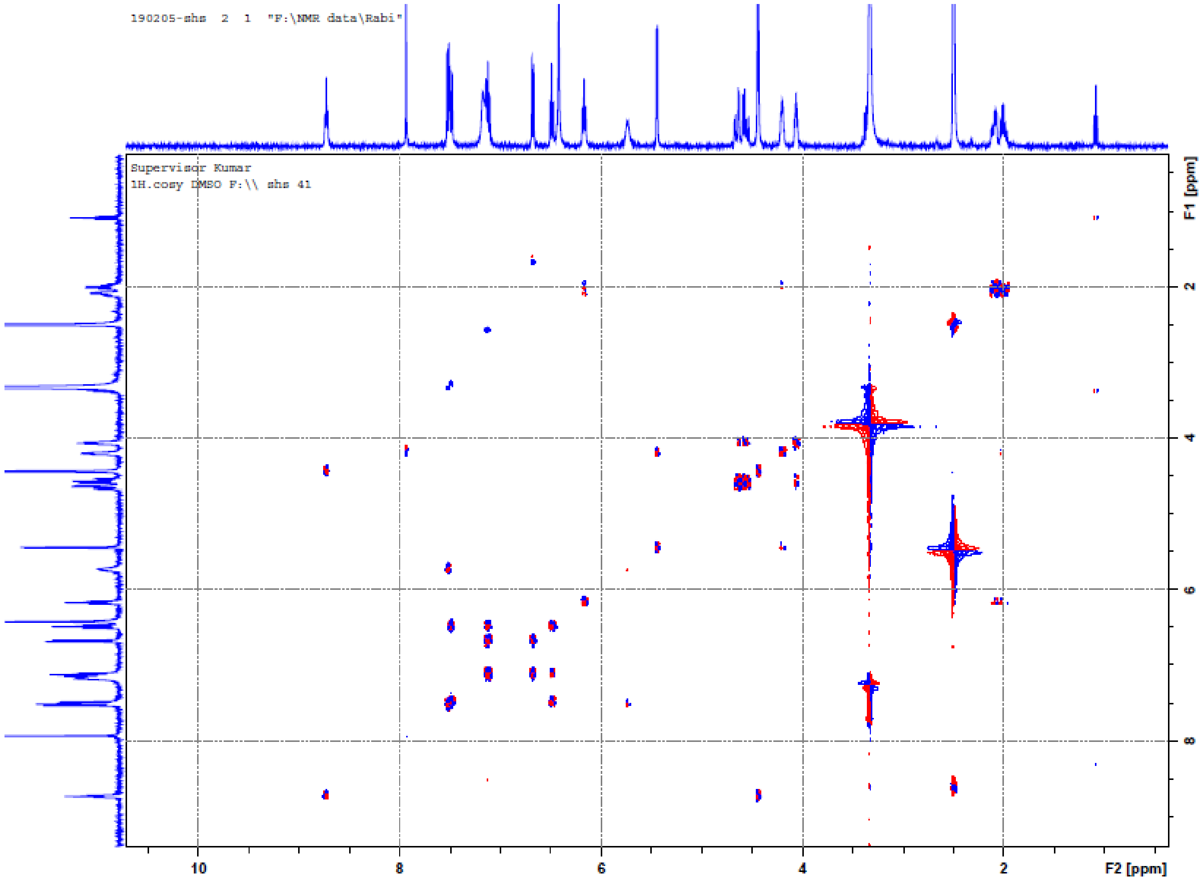

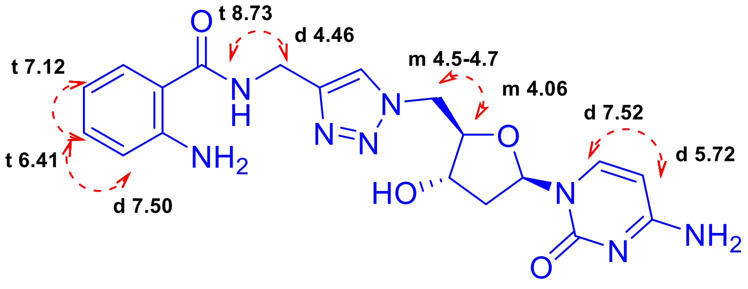

^1H-^{13}C HMBC NMR spectrum of compound 13a

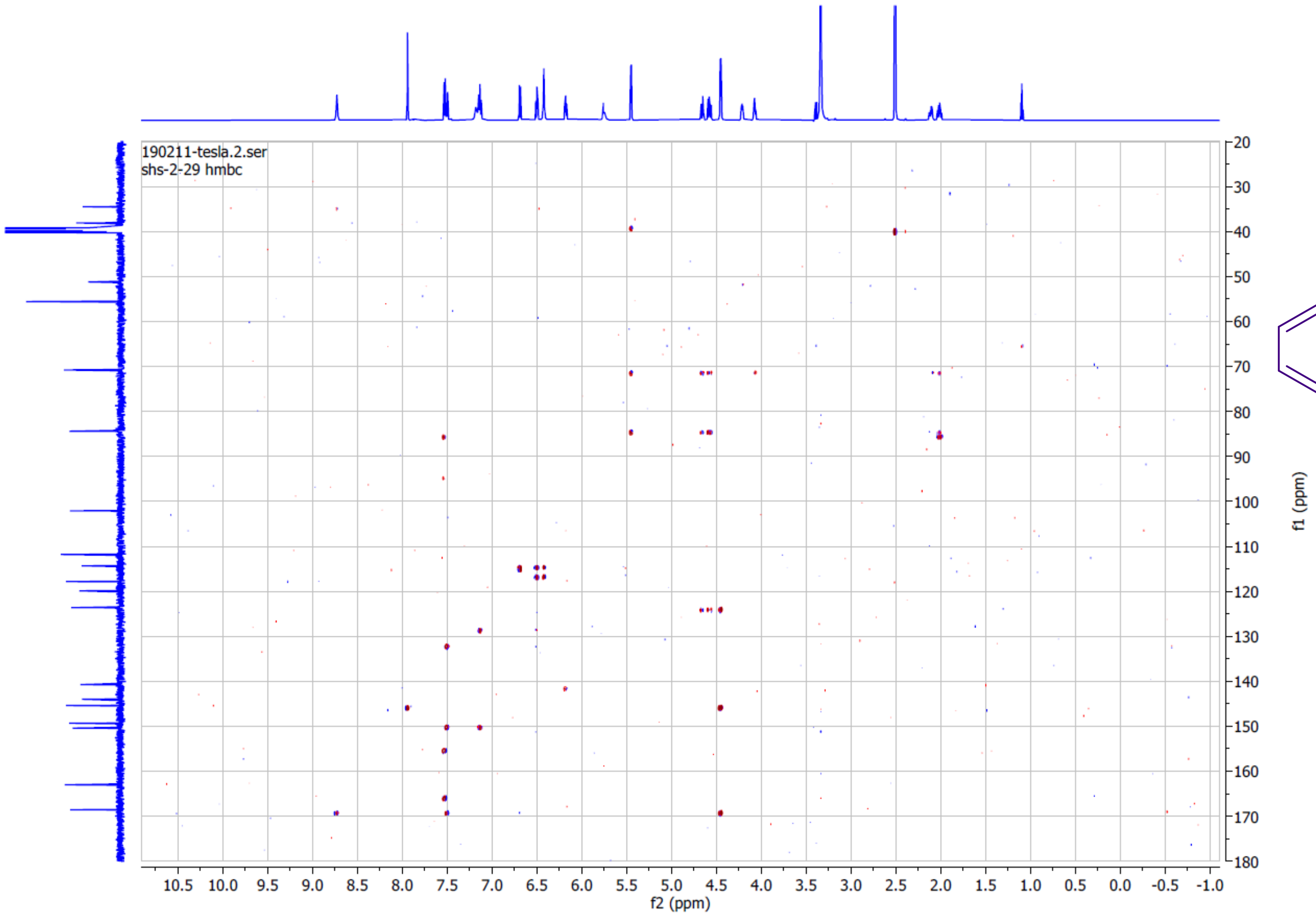

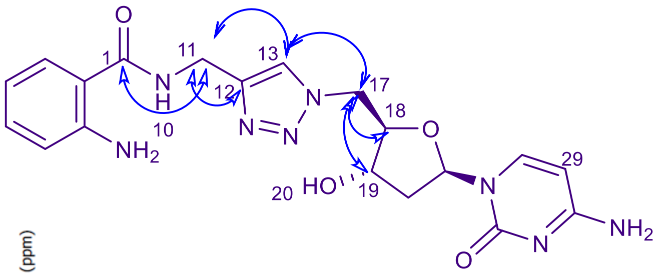

^{1}H- ^{13}C HSQC NMR spectrum of compound 13a

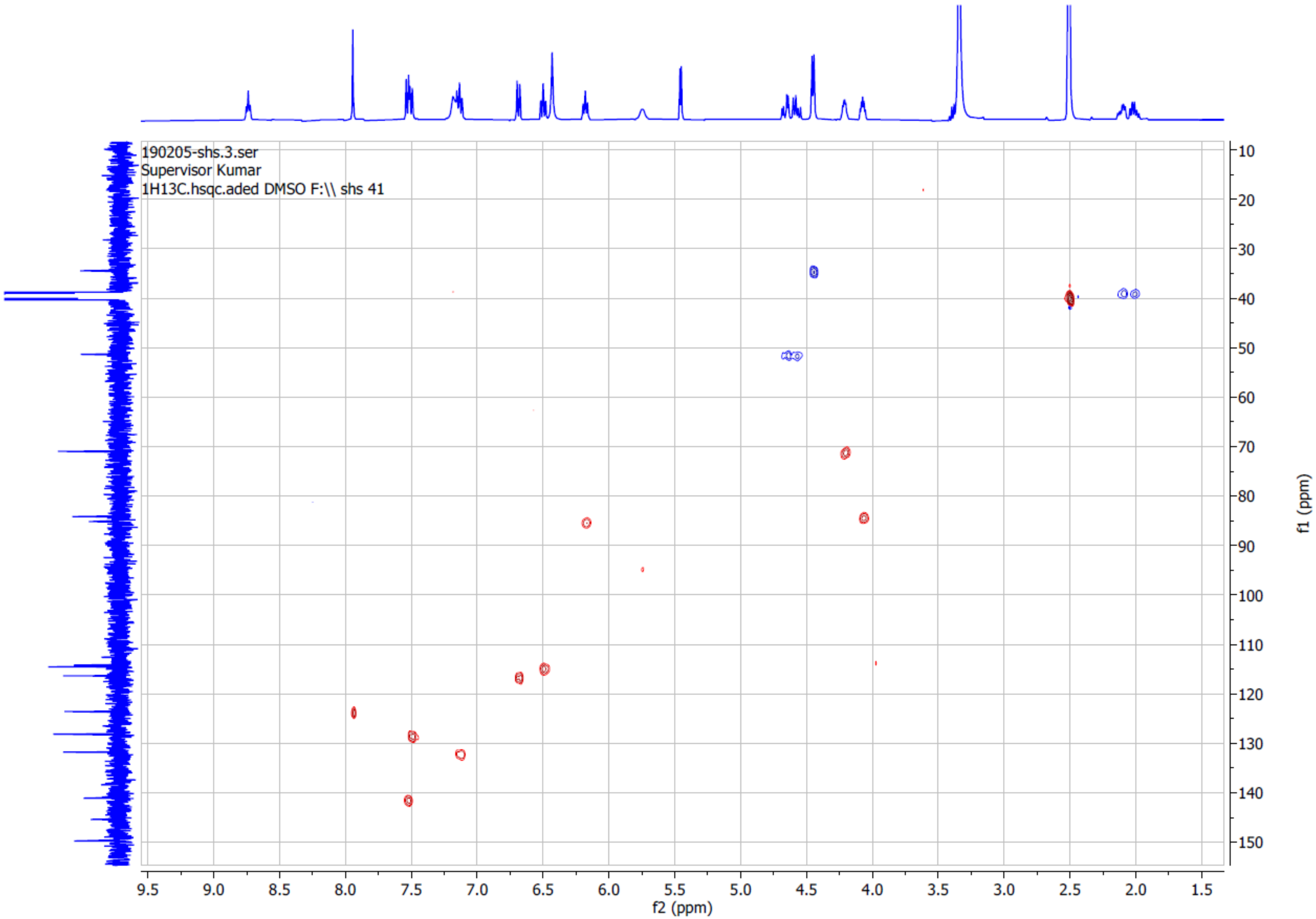

DEPT 135 NMR spectrum of compound 13a

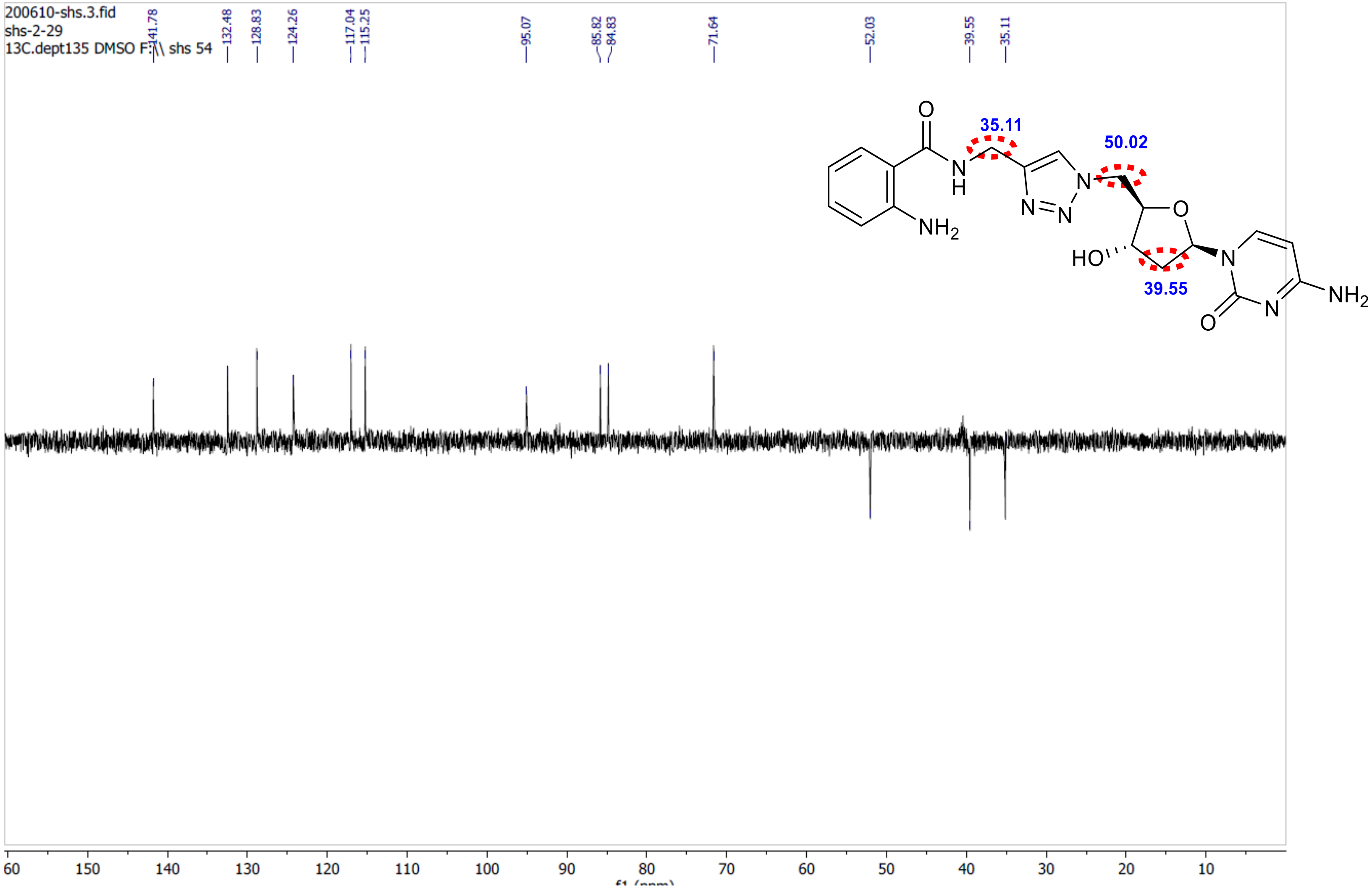

^{1}H NMR signal assignment of compound 7a

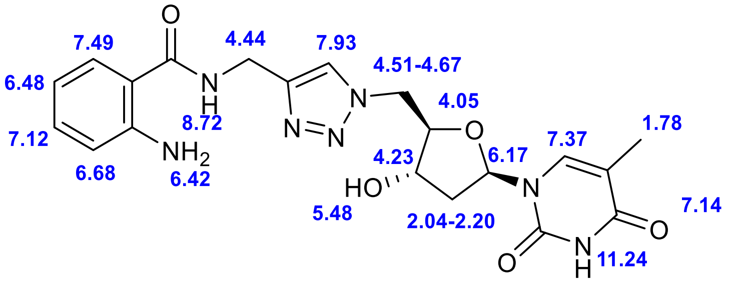

δ (ppm)	Peak Multiplicity	Peak Integration	Group
1.78	d	3H	CH_3
2.04-2.20	m	2H	CH_2
4.05-4.09	m	1H	CH
4.23-4.30	m	1H	CH
4.44	d	2H	CH_2
4.51-4.67	m	2H	CH_2
5.48	d	1H	OH
6.17	t	1H	CH
6.42	s	2H	NH_2
6.48	td	1H	Ar-H
6.68	dd	1H	Ar-H
7.12	td	1H	Ar-H
7.37	d	2H	NH_2
7.49	dd	1H	Ar-H
7.93	s	1H	Ar-H
8.72	t	1H	NH
11.24	bs	1H	NH

^{13}C NMR signal assignment of compound 7a

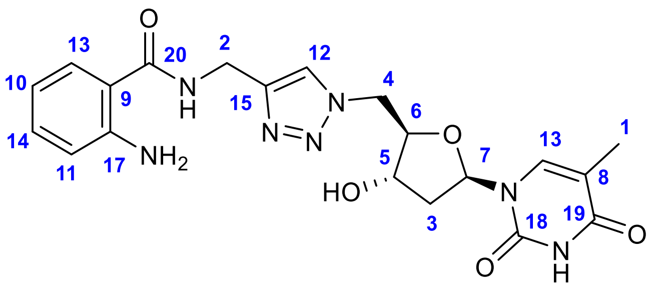

δ (ppm)	Carbon number
12.04	1
34.46	2
37.86	3
51.13	4
70.79	5
84.02	6
84.07	7
109.88	8
114.15	9
114.50	10
116.35	11
123.59	12
128.11	13
131.79	14
136.01	15
145.45	16
149.77	17
150.41	18
163.64	19
168.80	20

^{1}H NMR signal assignment of compound 9a

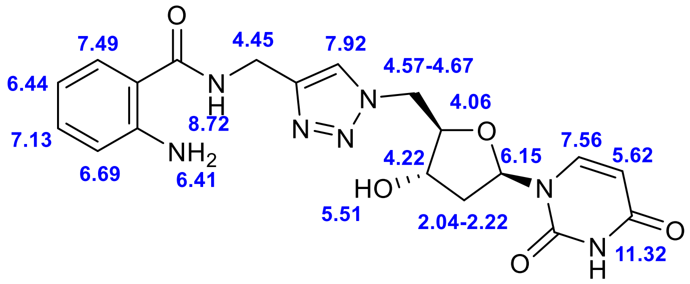

δ (ppm)	Peak Multiplicity	Peak Integration	Group
2.04-2.22	m	2H	CH_2
4.06-4.10	m	1H	CH
4.22-4.25	m	1H	CH
4.45	d	2H	CH_2
4.57	dd	1H	CH_2
4.67	dd	1H	CH_2
5.51	s	1H	OH
5.62	dd	1H	Ar-H
6.15	t	1H	CH
6.41	s	2H	NH_2
6.44-6.56	m	1H	Ar-H
6.69	dd	1H	Ar-H
7.13	td	1H	Ar-H
7.49	dd	1H	Ar-H
7.56	d	1H	Ar-H
7.92	s	1H	Ar-H
8.72	t	1H	NH
11.32	bs	1H	NH

^{13}C NMR signal assignment of compound 9a

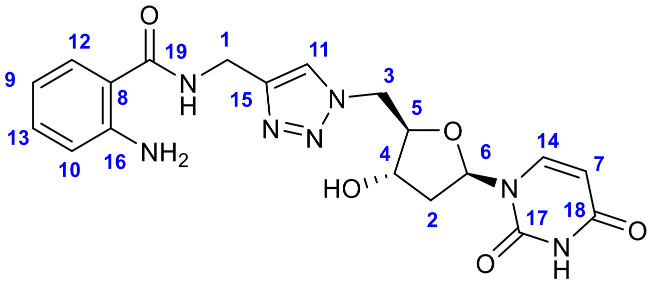

δ (ppm)	Carbon number
34.49	1
38.08	2
51.20	3
70.78	4
84.28	5
84.42	6
102.11	7
114.26	8
114.59	9
116.40	10
123.59	11
128.15	12
131.82	13
140.72	14
145.46	15
149.74	16
150.41	17
163.02	18
168.85	19

^{1}H NMR signal assignment of compound 13a

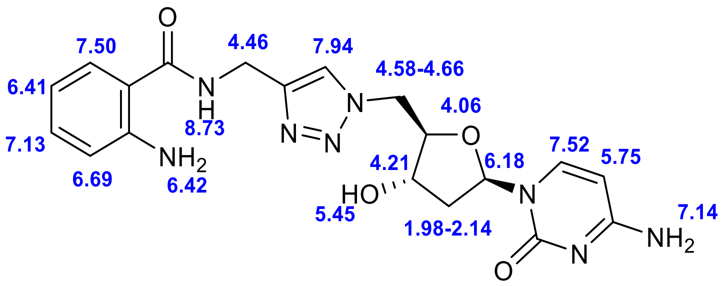

δ (ppm)	Peak Multiplicity	Peak Integration	Group
1.98-2.05	m	1H	CH_2
2.08-2.14	m	1H	CH_2
4.06-4.09	m	1H	CH
4.21-4.22	m	1H	CH
4.45	d	2H	CH_2
4.58	dd	1H	CH_2
4.66	dd	1H	CH_2
5.45	d	1H	OH
5.75	d	1H	Ar-H
6.18	t	1H	CH
6.42	s	2H	NH_2
6.50	td	1H	Ar-H
6.69	dd	1H	Ar-H
7.13	td	1H	Ar-H
7.14	s	2H	NH_2
7.50	dd	1H	Ar-H
7.52	d	1H	Ar-H
7.94	s	1H	Ar-H
8.73	t	1H	NH

^{13}C NMR signal assignment of compound 13a

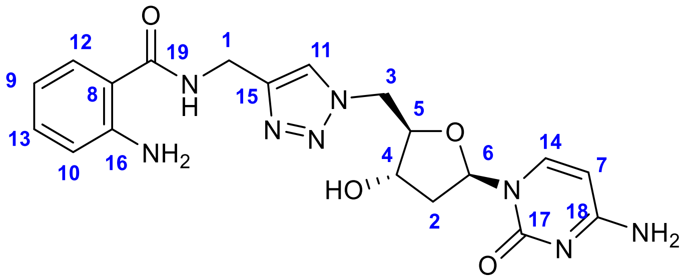

δ (ppm)	Carbon Number
34.48	1
39.55	2
51.38	3
70.98	4
84.17	5
85.17	6
94.41	7
114.25	8
114.06	9
116.39	10
123.60	11
128.17	12
131.83	13
141.12	14
145.42	15
149.76	16
154.99	17
165.55	18
168.84	19

pqs:gfp reporter assay

- MHB
- PAO1
- Compounds dissolved in DMSO to a concentration of 10 mg/mL and used at a maximum concentration of 125 μg/mL
- Experiment carried out once for each compound with 3 technical replicates per experiment
- Control experiment carried out with DMSO alone; no test compound added. GFP fluorescence is inhibited at 5% and 2.5% equivalent to 500 ug/ml and 250 ug/ml of any test compound. The legend shows corresponding concentration test compound (if present); At 1.25 % and 0.625% DMSO GFP fluorescence is not affected.
- Positive control is itaconimide derivative (18a)[1]

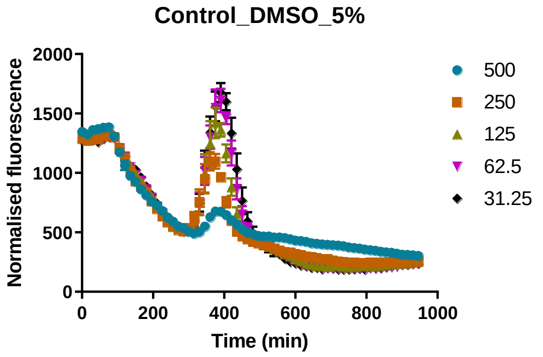

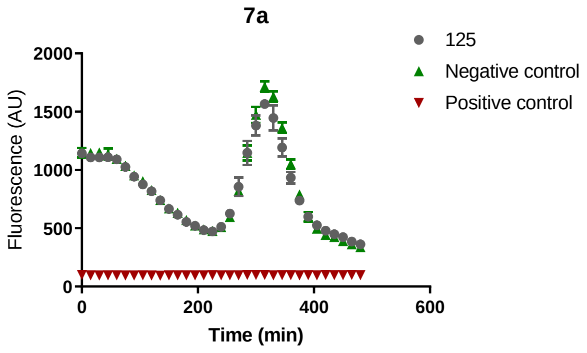

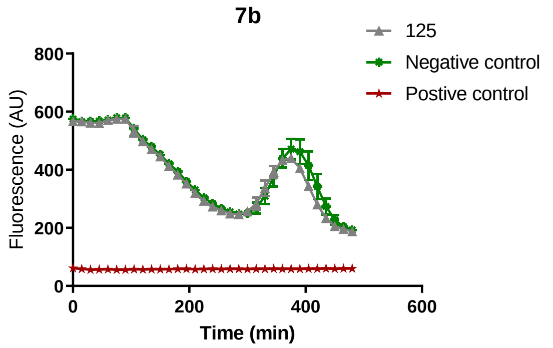

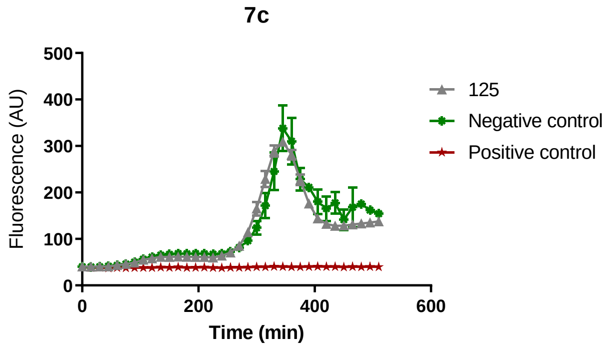

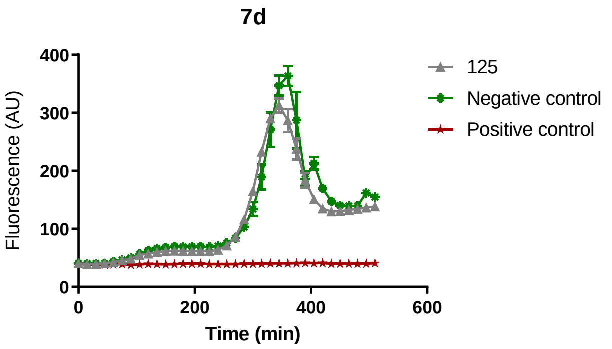

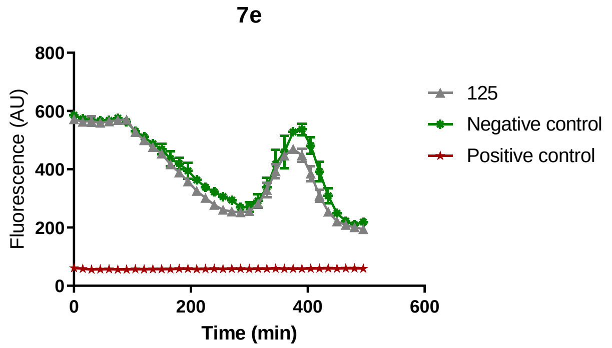

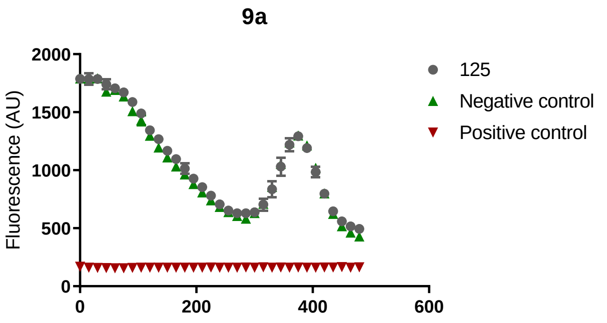
9a
Fluorescence (AU)
2000
1500
1000
500
0
0
200
400
600
125
Negative control
Positive control

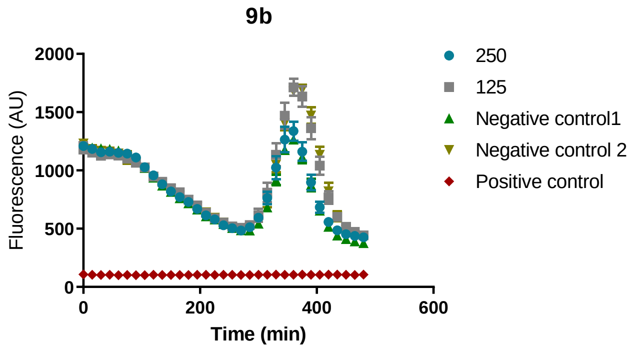
9b
Fluorescence (AU)
Time (min)
2000
1500
1000
500
0
0
200
400
600
250
125
Negative control1
Negative control 2
Positive control

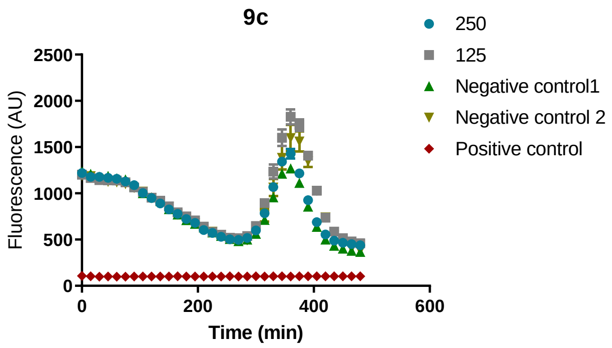
9c
Fluorescence (AU)
Time (min)
2500
2000
1500
1000
500
0
0
200
400
600
250
125
Negative control1
Negative control 2
Positive control

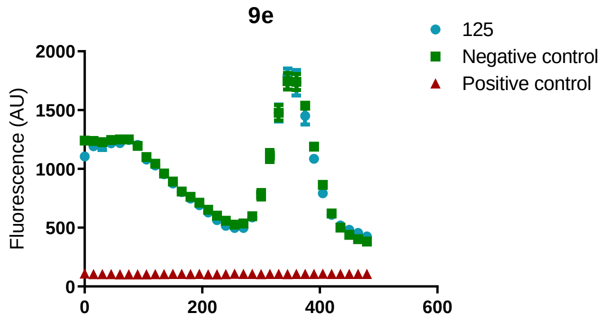
9e
Fluorescence (AU)
2000
1500
1000
500
0
0
200
400
600
125
Negative control
Positive control

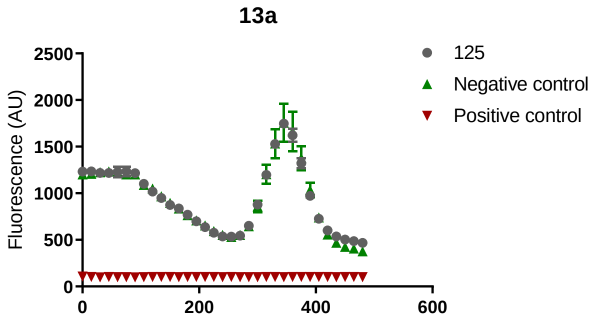
13a
Fluorescence (AU)
2500
2000
1500
1000
500
0
0
200
400
600
125
Negative control
Positive control

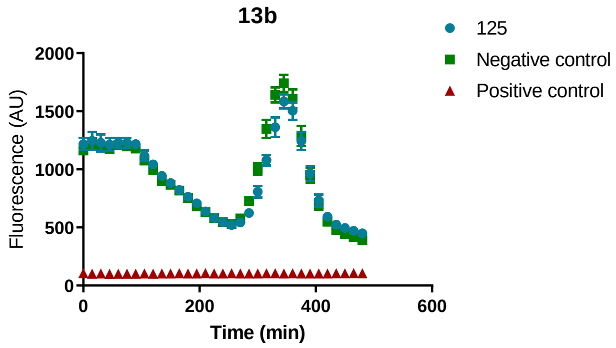
13b
Fluorescence (AU)
Time (min)
2000
1500
1000
500
0
0
200
400
600
125
Negative control
Positive control

1.

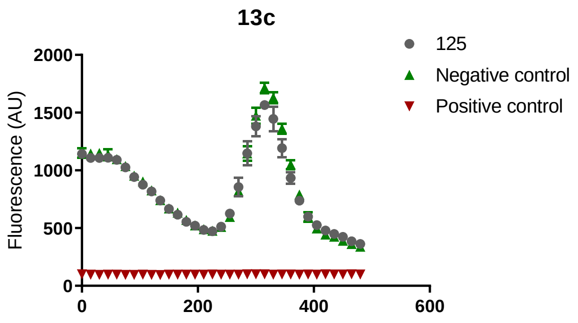

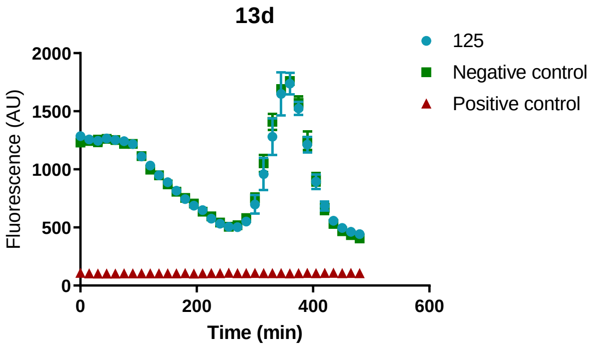

1. Fong, J.; Mortensen, K.T.; Nørskov, A.; Qvortrup, K.; Yang, L.; Tan, C.H.; Nielsen, T.E.; Givskov, M. Itaconimides as novel quorum sensing inhibitors of *Pseudomonas aeruginosa*. *Front Cell Infect Microbiol* **2019**, *8*, 443.

MIC of QS inhibitor compounds

- MHB
- PAO1 (~5 * 10^5 CFU/mL per well)
- Compounds dissolved in DMSO to a concentration of 10 mg/mL and used at a maximum concentration of 512 μg/mL
- Experiment carried out once for each compound with 3 technical replicates per experiment

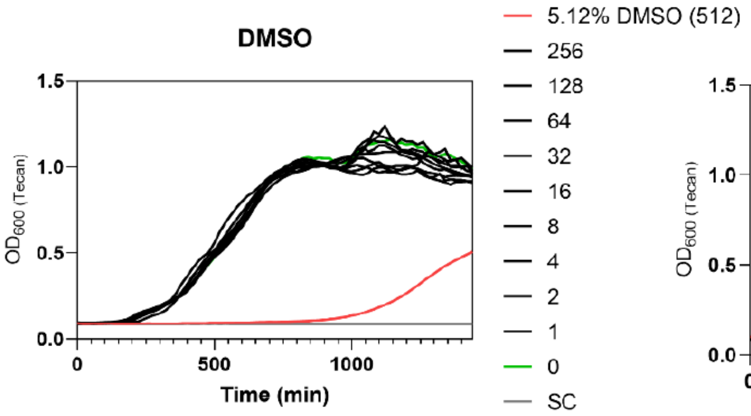

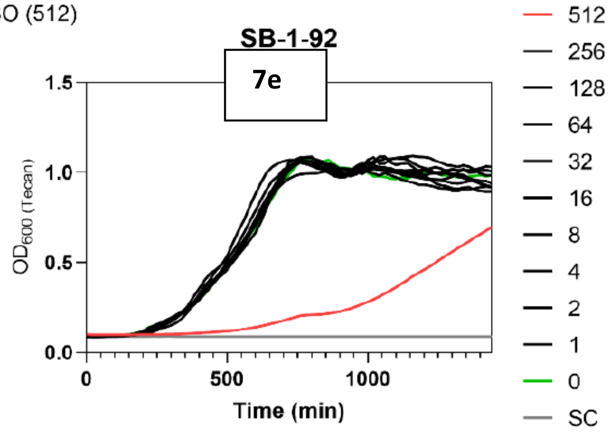

7a

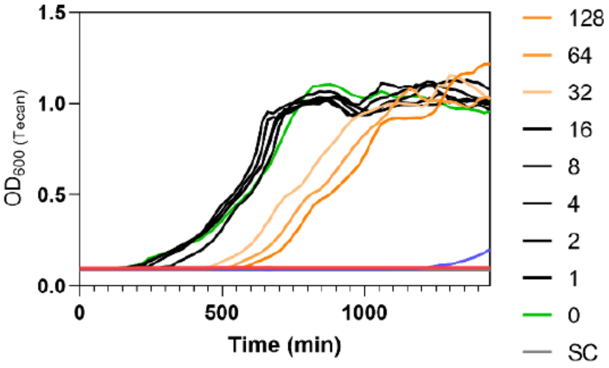
1.5
1.0
0.5
0.0
OD600 (Tecan)
0
500
1000
Time (min)
512
256
128
64
32
16
8
4
2
1
0
SC

7b

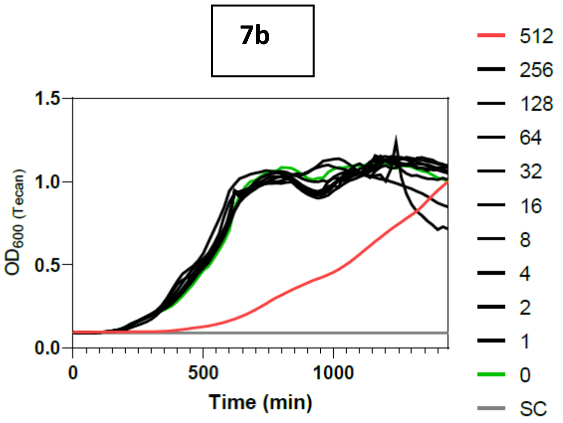
1.5
1.0
0.5
0.0
OD600 (Tecan)
0
500
1000
Time (min)
512
256
128
64
32
16
8
4
2
1
0
SC

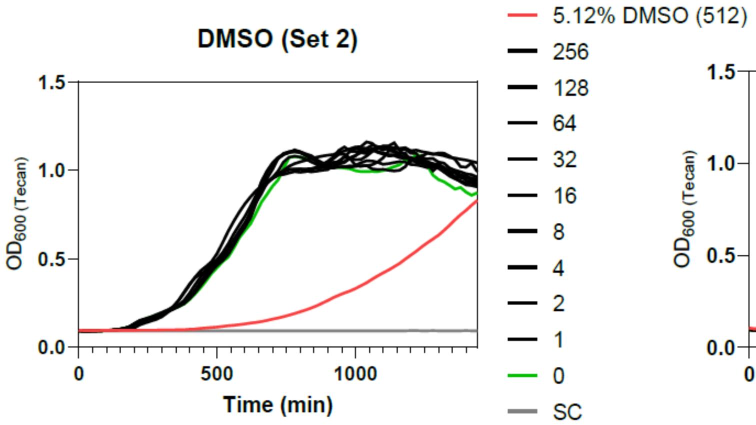
DMSO (Set 2)
1.5
1.0
0.5
0.0
OD600 (Tecan)
0
500
1000
Time (min)
5.12% DMSO (512)
256
128
64
32
16
8
4
2
1
0
SC

7c

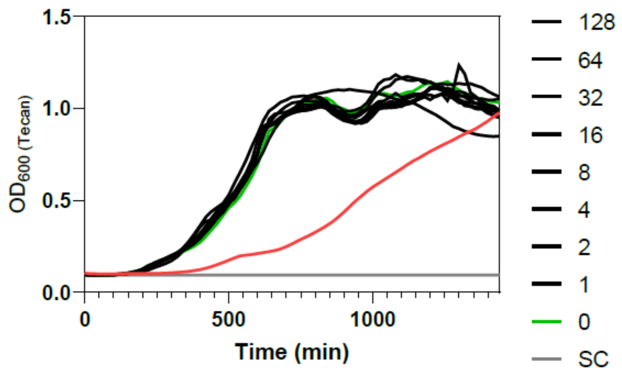
1.5
1.0
0.5
0.0
OD600 (Tecan)
0
500
1000
Time (min)
512
256
128
64
32
16
8
4
2
1
0
SC

7d

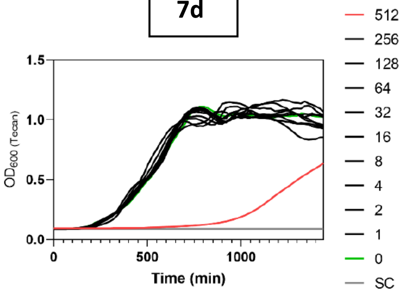
1.5
1.0
0.5
0.0
OD600 (Tecan)
0
500
1000
Time (min)
512
256
128
64
32
16
8
4
2
1
0
SC

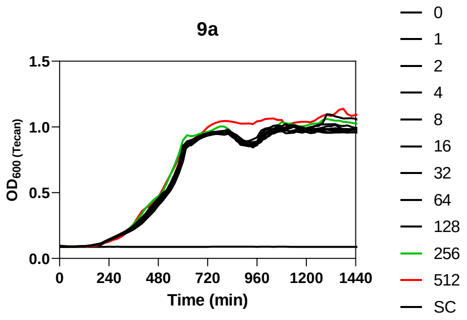
9a
1.5
1.0
0.5
0.0
OD600 (Tecan)
0
240
480
720
960
1200
1440
Time (min)
0
1
2
4
8
16
32
64
128
256
512
SC

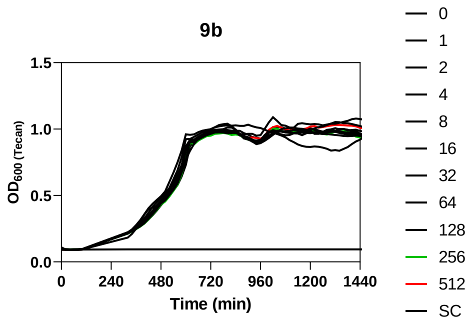
9b
1.5
1.0
0.5
0.0
OD600 (Tecan)
0
240
480
720
960
1200
1440
Time (min)
0
1
2
4
8
16
32
64
128
256
512
SC

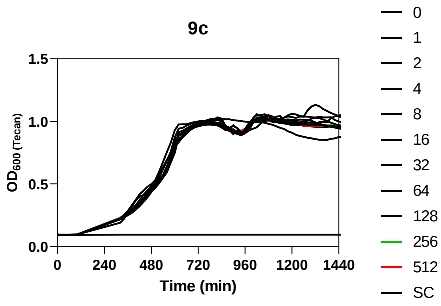
9c
1.5
1.0
0.5
0.0
OD600 (Tecan)
0
240
480
720
960
1200
1440
Time (min)
0
1
2
4
8
16
32
64
128
256
512
SC

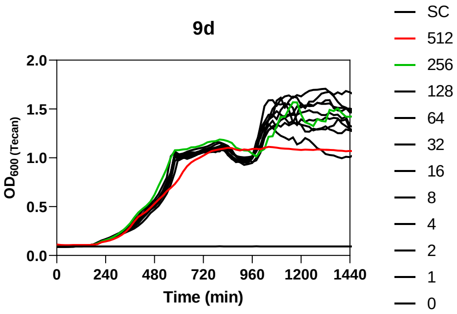
9d
OD600 (Tecan)
2.0
1.5
1.0
0.5
0.0
0
240
480
720
960
1200
1440
Time (min)
SC
512
256
128
64
32
16
8
4
2
1
0

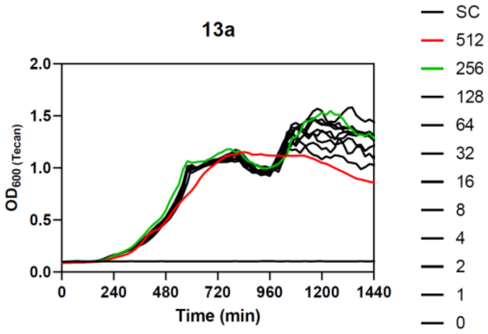
13a
OD600 (Tecan)
2.0
1.5
1.0
0.5
0.0
0
240
480
720
960
1200
1440
Time (min)
SC
512
256
128
64
32
16
8
4
2
1
0

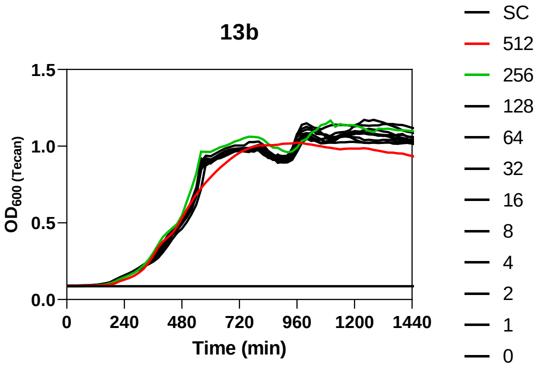
13b
OD600 (Tecan)
1.5
1.0
0.5
0.0
0
240
480
720
960
1200
1440
Time (min)
SC
512
256
128
64
32
16
8
4
2
1
0

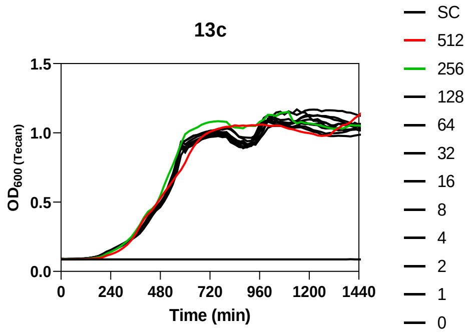
13c
OD600 (Tecan)
1.5
1.0
0.5
0.0
0
240
480
720
960
1200
1440
Time (min)
SC
512
256
128
64
32
16
8
4
2
1
0

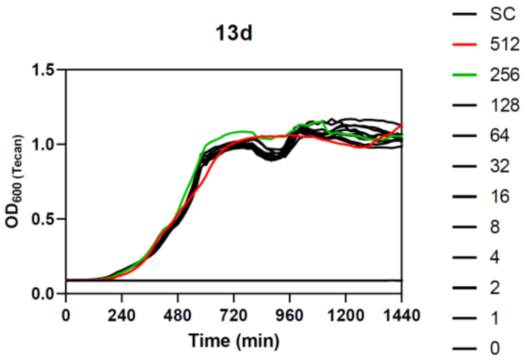
13d
OD600 (Tecan)
1.5
1.0
0.5
0.0
0
240
480
720
960
1200
1440
Time (min)
SC
512
256
128
64
32
16
8
4
2
1
0

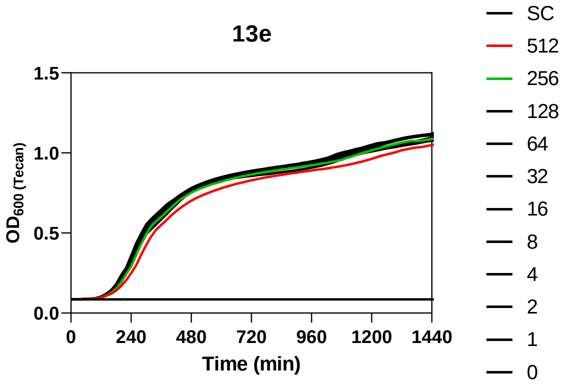
13e
OD600 (Tecan)
1.5
1.0
0.5
0.0
0
240
480
720
960
1200
1440
Time (min)
SC
512
256
128
64
32
16
8
4
2
1
0
